# Molecular and genetic organization of bands and interbands in the dot chromosome of *Drosophila melanogaster*

**DOI:** 10.1007/s00412-019-00703-x

**Published:** 2019-04-30

**Authors:** Darya S. Sidorenko, Ivan A. Sidorenko, Tatyana Yu. Zykova, Fedor P. Goncharov, Jan Larsson, Igor F. Zhimulev

**Affiliations:** 1 Institute of Molecular and Cellular Biology of the Siberian Branch of the Russian Academy of Sciences, Lavrentiev Ave. 8/2, Novosibirsk, Russia 630090; 2 Institute of Cytology and Genetics of the Siberian Branch of the Russian Academy of Sciences, Novosibirsk, Russia; 3 Department of Molecular Biology, Umeå University, Umeå, Sweden; 4 Laboratory of structural, functional and comparative genomics of the Novosibirsk State University, Novosibirsk, Russia

**Keywords:** *Drosophila*, Polytene chromosomes, Dot chromosome, Chromatin types, Bands, Interbands

## Abstract

The fourth chromosome smallest in the genome of *Drosophila melanogaster* differs from other chromosomes in many ways. It has high repeat density in conditions of a large number of active genes. Gray bands represent a significant part of this polytene chromosome. Specific proteins including HP1a, POF, and dSETDB1 establish the epigenetic state of this unique chromatin domain. In order to compare maps of localization of genes, bands, and chromatin types of the fourth chromosome, we performed FISH analysis of 38 probes chosen according to the model of four chromatin types. It allowed clarifying the dot chromosome cytological map consisting of 16 loose gray bands, 11 dense black bands, and 26 interbands. We described the relation between chromatin states and bands. Open aquamarine chromatin mostly corresponds to interbands and it contains 5′UTRs of housekeeping genes. Their coding parts are embedded in gray bands substantially composed of lazurite chromatin of intermediate compaction. Polygenic black bands contain most of dense ruby chromatin, and also some malachite and lazurite. Having an accurate map of the fourth chromosome bands and its correspondence to physical map, we found that DNase I hypersensitivity sites, ORC2 protein, and *P*–elements are mainly located in open aquamarine chromatin, while element *1360*, characteristic of the fourth chromosome, occupies band chromatin types. POF and HP1a proteins providing special organization of this chromosome are mostly located in aquamarine and lazurite chromatin. In general, band organization of the fourth chromosome shares the features of the whole *Drosophila* genome.

**Electronic supplementary material:**

The online version of this article (10.1007/s00412-019-00703-x) contains supplementary material, which is available to authorized users.

## Introduction

Genetic activity of interphase chromosomes depends on their structural organization, but the basis of this interconnection has not been studied in sufficient detail. Polytene chromosomes of *Drosophila melanogaster* are a convenient model for such studies. In contrast to the chromosomes of diploid cells, polytene chromosomes can be observed along their entire length in the interphase nucleus. These structures are formed due to multiple rounds of DNA replication without subsequent segregation of daughter chromatids. For this reason, the chromomeric pattern becomes represented in the form of alternating transverse stripes—black compact bands, less compact gray bands, and loose light-colored interbands. Due to the unique polytene chromosome banding pattern, C. Bridges performed detailed chromosome drawings with simple symbols (Bridges 1935a). These maps are actively used in research at the present time.

Despite obvious morphological features, polytene chromosomes demonstrate universal principle of interphase chromosome organization in all cell types, which has been shown in detail in our previous works (Zhimulev et al. 2014; Zykova et al. 2018). Giant size of polytene chromosomes and the banding pattern, unique for each region, allow connecting molecular and genetic characteristics of chromatin with morphological organization of interphase chromosomes with high resolution. To achieve this goal, it is necessary to localize the DNA sequence on the cytological map of polytene chromosomes.

Sequencing of the *D. melanogaster* genome has provided an opportunity to study structural and functional organization of chromosomes in more detail. So far, a large amount of genomic data on localization of proteins and regulatory elements on interphase chromosomes in cell cultures undergoing mitosis have been accumulated in the modENCODE project (model organism Encyclopedia of DNA Elements). Recently, researchers have faced the problem of transforming the obtained information into functional maps that characterize the processes of transcription, replication, splicing, and epigenetic changes regulation (The modENCODE Consortium et al. 2010). In recent studies, various types of *Drosophila* chromatin have been distinguished using computer modeling and the methods of DamID (DNA adenine methyltransferase identification), CHIP-on-chip (chromatin immunoprecipitation on chip), and the determination of the overall chromatin sensitivity to DNase I (Filion et al. 2010; Kharchenko et al. 2011; Milon et al. 2014). The DamID definition of five chromatin types is based on the distribution of 53 diverse proteins obtained in DamID experiments (Filion et al. 2010). The authors note that the fourth chromosome (and pericentric heterochromatin) is mainly enriched in GREEN chromatin that corresponds to classic heterochromatin marked by SU(VAR)3-9, HP1, and the HP1-interacting proteins LHR and HP6 (Filion et al. 2010). The modENCODE consortium identified the prevalent combinatorial patterns of 18 histone modifications (CHIP-on-chip data) and determined nine combinatorial chromatin states (Kharchenko et al. 2011). The fourth chromosome and pericentric heterochromatin are characterized by high levels of H3K9me2/me3 (state 7, dark blue) (Kharchenko et al. 2011). The 3CM model created by Milon et al. (2014) is based on general chromatin sensitivity to DNase I. This model revealed three states: open, closed, and neutral (Milon et al. 2014). According to 3CM, chromosome 4 and pericentric heterochromatin are enriched in neutral chromatin. Therefore, chromosome 4 appears to share similarities with both euchromatin and heterochromatin (Milon et al. 2014). However, the correlation of these chromatin types (Filion et al. 2010; Kharchenko et al. 2011; Milon et al. 2014) based on different input data with morphological structures of polytene chromosomes remains a topic of current interest.

In our laboratory, we developed a unique approach of determining interband localization in the genome of *Drosophila*. It is based on *P*–element–mediated transformation of DNA, which results in compaction of the construction inserted. If the insertion occurs in a band, there are no changes in polytene chromosome structure. In case of insertion localization in an interband, a new band is formed (Semeshin et al. 1986; Demakov et al. 1993; Vatolina et al. 2011b). Sequencing the DNA surrounding the insertions allowed us to determine the genomic position of 12 interbands. Using modENCODE data on the distribution of chromatin proteins in mitotically dividing cells, we defined a set of proteins that enrich DNA sequences corresponding to these interbands previously located on the physical map of the *Drosophila* genome (Demakov et al. 2011; Vatolina et al. 2011a). Next, an algorithm based on the distribution of these open chromatin proteins that allows determining the interband localization in the large arms of *D. melanogaster* polytene chromosomes was developed. Using this algorithm, four types of chromatin, conventionally named cyan, blue, magenta, and green, were identified (Zhimulev et al. 2014). Later, in order to avoid confusing caused by the intersection of names with other works, these chromatin types were renamed aquamarine, lazurite, ruby, and malachite, respectively (Khoroshko et al. 2016). The advantage of this model is a good correspondence of these chromatin types to a number of bands and interbands with already determined position on the molecular map. Aquamarine chromatin occupies about 13% of the genome and is enriched with open chromatin proteins. This type of chromatin represents interbands previously localized on the physical map of the genome. Ruby chromatin occupies almost half of the genome and is completely devoid of open chromatin proteins. It corresponds to black bands and intercalary heterochromatin bands. Malachite and lazurite do not demonstrate specificity about the “model” open chromatin proteins; they often correspond to gray bands (for more details about the model, see (Zhimulev et al. 2014; Boldyreva et al. 2017; Zykova et al. 2018)).

Thus, the model of four chromatin states allows determining the localization of interbands and bands on the physical map of the *D. melanogaster* genome, a problem that has not been solved using other chromatin state models. After matching cytological and physical maps, it is possible to investigate the molecular characteristics of these polytene chromosome morphological structures using whole genome data. In the present work, we focused on chromatin organization of the *D. melanogaster* fourth chromosome.

The fourth chromosome is the smallest one in the *Drosophila* genome. Most of the 4.2 Mb fourth chromosome is dense heterochromatin; the euchromatic arm of this chromosome occupies only 1.2 Mb (Locke and McDermid 1993). Therefore, this chromosome looks like a “dot” on the metaphase chromosome preparations (Gatti et al. 1976; Pimpinelli et al. 1976; Pokholkova et al. 1993). After numerous cycles of endoreplication, in the polyploid nuclei of salivary gland cells, large blocks of heterochromatin become underrepresented, and the small euchromatic arm of the dot chromosome becomes visible. C. Bridges divided the small fourth chromosome into 101 and 102 sections and letter subsections; however, the cytological map has not been completed, and the bands did not receive individual numbers. A large number of gray bands that have an intermediate degree of compaction and the tendency of its tip to conjugate with the chromocenter ectopically causes a certain complexity in the mapping of the polytene fourth chromosome. In the current investigation, for a better spreading of the polytene dot chromosome on the preparations, we used the fly stock with suppressed underreplication.

The fourth chromosome of *Drosophila* has an unusual organization. This chromosome shares the characteristics of both eu- and heterochromatin and a number of unique properties (Riddle and Elgin 2006; Riddle et al. 2009). Gene density in this chromosome corresponds to euchromatin. At the same time, there is no recombination in the dot chromosome (Sandler and Szauter 1978). The absence of crossing-over leads to accumulation of repeated sequences and mobile elements of the genome. Repeat density in the fourth chromosome is similar to that in pericentric heterochromatin (Slawson et al. 2006). The *1360* (Kholodilov et al. 1988) and *DINE-1* (Locke et al. 1999) elements are predominantly concentrated on this chromosome. The transgenic constructs based on *P*-transposons often demonstrate variegating phenotype of the reporter gene (Sun et al. 2000; Riddle et al. 2008; for more details about position effect variegation, see Elgin and Reuter 2013). In addition, such heterochromatin mark as methylation of H3K9, which is mainly introduced by specific for this chromosome histone methyltransferase dSETDB1 (Tzeng et al. 2007; Figueiredo et al. 2012), is associated with the fourth chromosome. Typical heterochromatin protein HP1a that recognizes H3K9 methylation binds the fourth chromosome (James et al. 1989), although its role in chromatin organization of this chromosome is still subject to discussion.

A unique feature of the fourth chromosome is POF (painting-of-fourth) protein presumably evolutionarily associated with the dose compensation system that *D. melanogaster* adapted to regulate the work of the fourth chromosome genes in heterochromatic environment (Larsson et al. 2001). POF can enhance transcription or RNA transport through nuclear pores by binding to nascent RNA (Johansson et al. 2012). Thus, an interactive network of epigenetic chromatin regulation, unique for this autosome, including dSETDB1, HP1a, and POF, is involved in maintaining the activity of the fourth chromosome genes in the heterochromatic environment (Seum et al. 2007; Tzeng et al. 2007; Brower-Toland et al. 2009) (for more details about the fourth chromosome organization, see Riddle and Elgin (2018)). We were interested to know whether the band organization of the fourth chromosome is influenced by the combination of characteristics mentioned above that distinguish the chromosome under investigation from other *Drosophila* autosomes.

In our work, we applied a combined approach of mapping the entire sections of polytene chromosomes using computer modeling, modENCODE data, electron microscopy, fluorescent in situ hybridization (FISH), and light microscopy to determine the genomic coordinates of the fourth chromosome bands and interbands, and clarified its cytological map. This allowed us to study in detail the genetic organization, the distribution of chromatin proteins, mobile genetic elements, and other elements of the genome in the morphological structures of this unique *D. melanogaster* chromatin domain using whole genome databases.

## Materials and methods

### Map of chromatin types

Four basic chromatin types (aquamarine, lazurite, malachite, and ruby) used in the current study were defined earlier in five large chromosome arms 2L, 2R, 3L, 3R, and X (Zhimulev et al. 2014; Boldyreva et al. 2017; Zykova et al. 2018). Aquamarine chromatin mostly matches interbands, ruby chromatin vice versa corresponds to dense black bands, and lazurite and malachite chromatin have an intermediate level of compactness and coincide with gray bands (Zhimulev et al. 2014; Zhimulev et al. 2016; Boldyreva et al. 2017). In this study, the same four chromatin types were identified in the fourth chromosome. Each chromatin type was composed of 200-bp-long non-overlapping fragments belonging to the euchromatic portion of the fourth chromosome. There are also some regions named gaps where the model failed to return a specific value. The model was smoothed: we combined the intervals of identical states if gaps between them did not exceed 400 bp. Using this four-chromatin-state model, the chromatin composition of the fourth chromosome morphological structures was determined.

### Fly stock

In the present work we used a fly stock with $\widehat{XY}\widehat{XY}$/$\widehat{XY}$; *y w; SuUR*^*ES*^*, Su(var)3-9*^*06*^ genotype because of suppressed underreplication of chromosome regions for better spreading of *Drosophila* fourth polytene chromosome on preparations (Andreyeva et al. 2007; Demakova et al. 2007). Flies were raised on standard cornmeal–yeast–agar–molasses medium (Semeshin et al. 2004). The fly stock was kindly provided by E. S. Belyaeva.

### Fluorescent in situ hybridization

Salivary glands were dissected in PBS solution and then fixed in a 3∶1 mixture of ethanol and acetic acid for 30 min at − 20 °C, squashed in 45% acetic acid, snap-frozen in liquid nitrogen, and stored in 70% ethanol at − 20 °C. Squashed polytene chromosome preparations together with electron microscopy images were used to refine the fourth chromosome cytological map. FISH on polytene chromosomes was performed as described (Ashburner et al. 2005). Thirty-eight DNA probes were selected according to the four chromatin states and obtained by standard PCR. Random-primed labeling of DNA probes with TAMRA or Fluorescein (Biosan) was done using Klenow enzyme. All the probes used in this study are described in Table S1 (probes from bands predicted by the four-chromatin-state model) and in Table S2 (probes from open chromatin predicted by the four-chromatin-state model). FISH preparations were analyzed using fluorescent microscopy and the probes were plotted on the cytological map by C. Bridges.

### Immunostaining of polytene chromosomes

The preparations were made according to Czermin et al. (2002). Immunostaining was performed using anti-POF antibodies (1:100; Larsson et al. 2001), anti-CHRIZ antibodies (1:600; Gortchakov et al. 2005), and fluorescent secondary antibodies. Images were taken with fluorescent microscope.

### Genetic organization of the fourth chromosome bands and interbands

The genetic content of the fourth chromosome morphological structures was analyzed using FlyBase release 5.50. Gene expression was determined according to FlyAtlas as the number of tissues where the particular gene is expressed (Chintapalli et al. 2007).

### Analysis of protein distribution in the fourth chromosome

In our work, we used the ChIP-chip and ChIP-seq data on protein mapping from the “Chromosomal Proteins” and “Regulatory Elements in *Drosophila*” projects by the modENCODE consortium (release 33).

Protein enrichment in the fourth chromosome chromatin types was compared to that in the whole genome. All analysis was performed in the R statistical environment version 3.3 and Bioconductor packages (Lawrence et al. 2009; Lawrence et al. 2013; R Core Team 2017). All statistical tests were two-sided and statistical significance was determined if *p* value was less than 0.001. Comparisons were made using the unpaired Wilcoxon test.

The distribution of ORC2 (modENCODE data) in different chromatin types and morphological structures of the fourth chromosome was analyzed in more detail using the algorithm we wrote in C++.

The program algorithm was as follows. First, we sorted all genes, and selected those that fell into the region under investigation. From the start of the 5′UTRs of these genes, we divided the region under investigation into fragments of 200 bp towards the gene and the intergenic spacer. Next, we counted the number of ORC2 binding sites in each fragment and normalized it to the number of gene transcripts, wherein we took into account different variants of gene localization in the region under investigation, including “head-to-head” orientation.

HP1a and POF distribution in S2 cells (modENCODE data) was studied using the UCSC Table Browser intersection tool (http://bit.ly/TableBrowserTool). Intersection was performed with chromatin types and morphological structures of the fourth chromosome.

POF (Lundberg et al. 2013a; Johansson and Larsson 2014) and H3K27me3 (Sher et al. 2012) distribution in larval salivary glands was studied using Galaxy tools (https://usegalaxy.org/).

SUUR distribution at the cytological level was studied using microscopy images kindly provided by Tatyana D. Kolesnikova.

### Analysis of transposable element insertion site distribution in the fourth chromosome

To analyze the distribution of *P*–element insertions within the euchromatic part of the fourth chromosome, we used insertion coordinates tagged “transposable_element_insertion_site” from FlyBase release 5.50 (107 transposable element insertion sites in the chromosome 4). Its distribution was analyzed in the four chromatin types and morphological structures of the fourth chromosome. The algorithm was the same as in the case of ORC2.

The amount of different chromatin types and morphological structures of the fourth chromosome occupied by the element *1360* and its remnants (59 items, modENCODE data) was determined using the UCSC Table Browser intersection tool (http://bit.ly/TableBrowserTool).

### Analysis of DNaseI hypersensitive sites distribution in the fourth chromosome

The distribution of DNaseI hypersensitivity sites (DHS) was analyzed in more detail using the algorithm we wrote in C++. The algorithm was the same as in the case of ORC2.

#### Data availability

The datasets generated during and/or analyzed during the current study are available from the corresponding author on reasonable request.

## Results

### Cytological maps of the fourth polytene chromosome

**Fig. 1:**
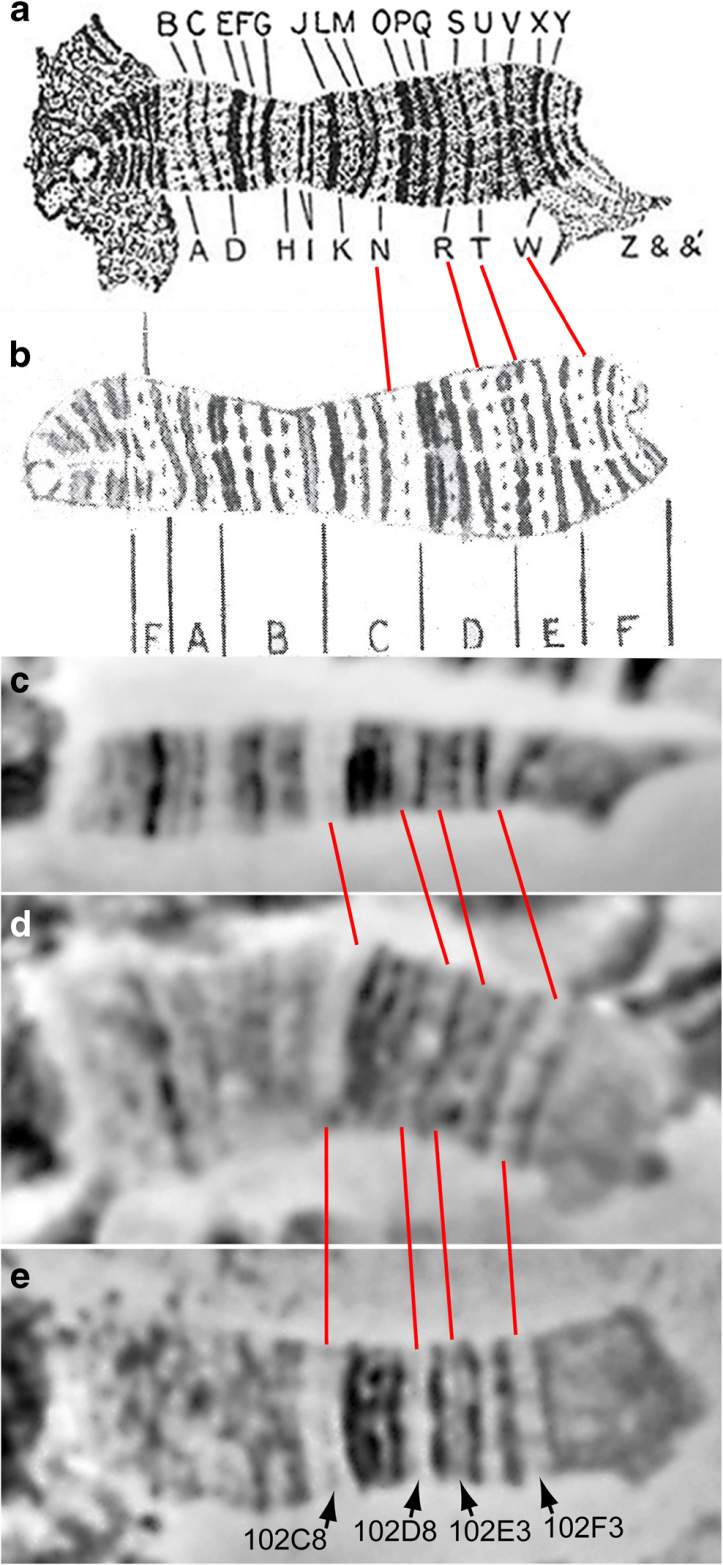
Thin gray bands in some regions of the fourth chromosome. **a** Illustration drawn by C. Bridges (Morgan et al. 1934). The drawing is practically the same as in the work by Bridges (1935b). **b** Drawing of C. Bridges (1935a). **c**–**e** Light microscopy of the fourth polytene chromosome. Arrowheads and red lines indicate faint gray bands 102C8, 102D8, 102E3, and 102F3

At the moment, there is no complete detailed cytological map of the fourth chromosome. Small size of the fourth chromosome and bending of its tip, which often tends to bind to the chromocenter ectopically, hinder its spreading on squashed chromosome preparations and, therefore, complicate analysis of its banding pattern. Initially, C. Bridges marked the fourth chromosome bands in capital letters A–Z and &, &′ (Morgan et al. 1934; Fig. 1a). On this map, up to 41 bands can be found taking into account thin dotted bands. On the second variant of his map, the polytenized part of the fourth chromosome is divided into 101 and 102 sections and letter subsections (Bridges 1935a; Fig. 1b). Only the section 101F-102F is polytenized (Fig. S1a). Researchers distinguished different numbers of bands of the dot polytene chromosome: from 35, if we count the bands of 101E subsection (Bridges 1935a), to 137 (Slizynski 1944). On polytene chromosome preparations, in fact, you can see much less bands than 137 (Fig. S1b, Fig. 1c–e); therefore, this number is considered to be unjustifiedly overestimated (Saura et al. 2002). In addition, according to Hochman and Slizynski, the fourth chromosome has a polytenized left arm (King 1975; Slizynski 1944); however, it is unclear whether this structure is indeed a 4L chromosome or an ectopically attached portion of another chromosome (Saura et al. 2002). The map by C. Bridges (Bridges 1935a; Fig.1b) depicts 37 bands of the polytene dot chromosome, if we consider all doublets to be single bands. However, C. Bridges did not complete the creation of a detailed fourth chromosome cytological map; the bands within the subsections did not receive individual numbers. On squashed preparations (Fig. S1b, Fig. 1c–e), one can see within 20–30 bands in the fourth polytene chromosome, depending on the extent of chromosome spreading and the microscopy resolution. Occasionally, the thinnest bands (marked with arrows in Fig. 1c–e) are seen on well-stretched chromosomes. In the present work, we focused on mapping the polytenized part of the fourth chromosome right arm 101E–102F, and compared our results with the maps by C. Bridges (Fig. 1a, b), electron-microscopic photograph of V. Semeshin (Fig. S1b), and band names from Saura et al. (2002). We made the resulting cytological map of the fourth chromosome based on the map by C. Bridges (1935a), band numbers by Saura et al. (2002), and our mapping efforts (Fig. S1a).

### Comparison of the *Drosophila* fourth chromosome cytological and physical maps

**Fig. 2:**
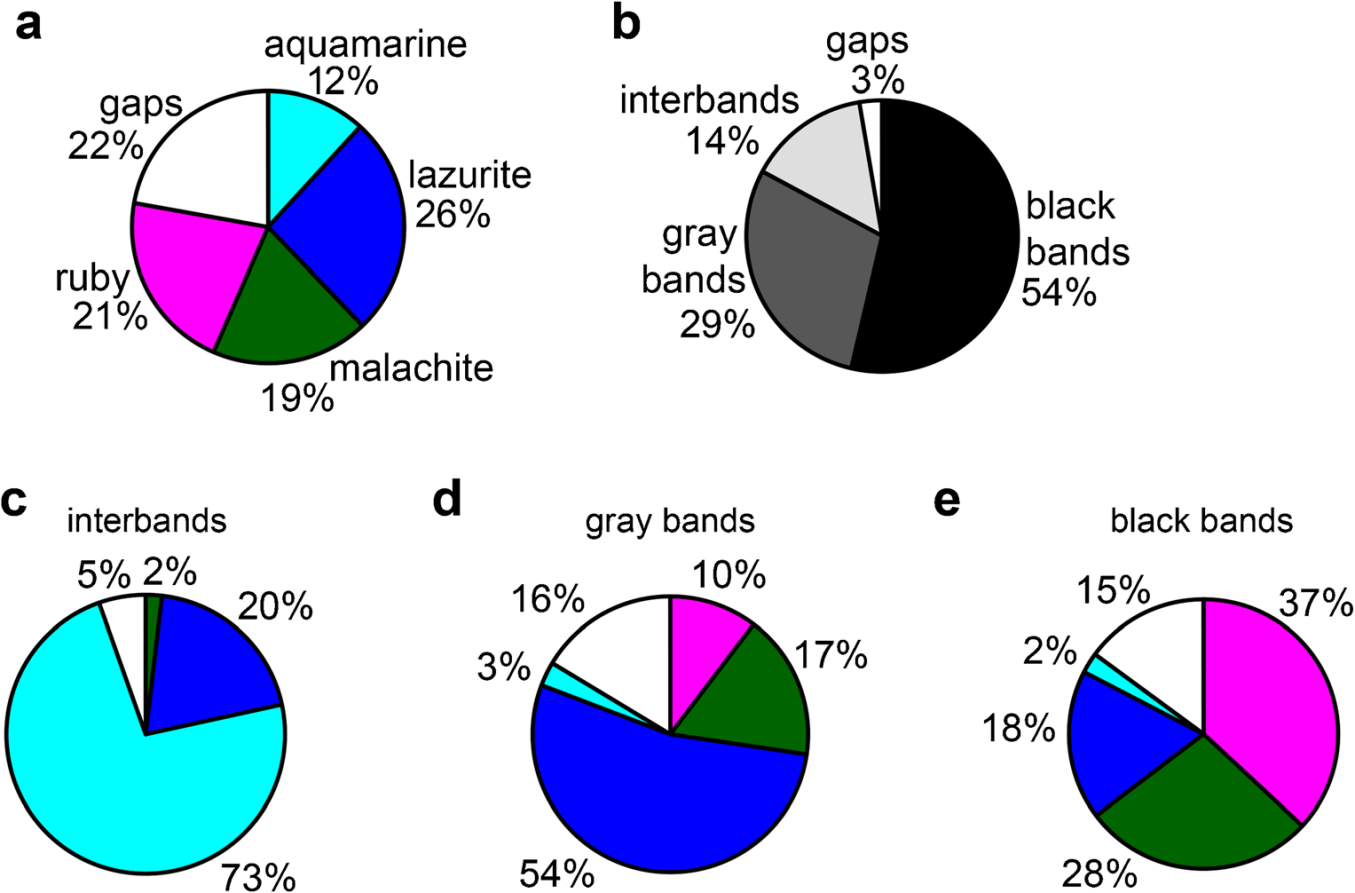
Chromatin and morphological composition of the fourth chromosome. **a** The percentage of the four chromatin types in the dot chromosome. Here and further, colors on the diagram correspond to chromatin types and white section corresponds to gaps, where the four-chromatin-state model failed to return a specific value. **b** The percentage of bands and interbands in the dot chromosome. Here and further, colors on the diagram correspond to morphological structures and white section corresponds to gaps, which were not included in bands or interbands. **c** Chromatin composition of the fourth chromosome interbands. **d** Chromatin composition of the fourth chromosome gray bands. **e** Chromatin composition of the fourth chromosome black bands

In this work, we constructed a four-chromatin-state model (ruby, malachite, lazurite, aquamarine) based on the distribution of open chromatin proteins colocalized with CHRIZ for the fourth chromosome of *Drosophila*. The program algorithm and these chromatin types defined for the remaining chromosomes have been described earlier (Zhimulev et al. 2014; Boldyreva et al. 2017; Materials and methods). Our choice of chromatin state model is substantiated in the Fig. S2 and the Supplementary text 1. It turned out that, in comparison with the large chromosome arms (Zhimulev et al. 2014), the portion of dense ruby chromatin in the fourth chromosome is two times smaller, but malachite and lazurite chromatin types with an intermediate degree of compaction are largely represented (Fig. 2a). This fact is in good agreement with the complex morphological organization of the fourth chromosome with a large number of gray bands.

### FISH probe localization on the polytene fourth chromosome

**Fig. 3:**
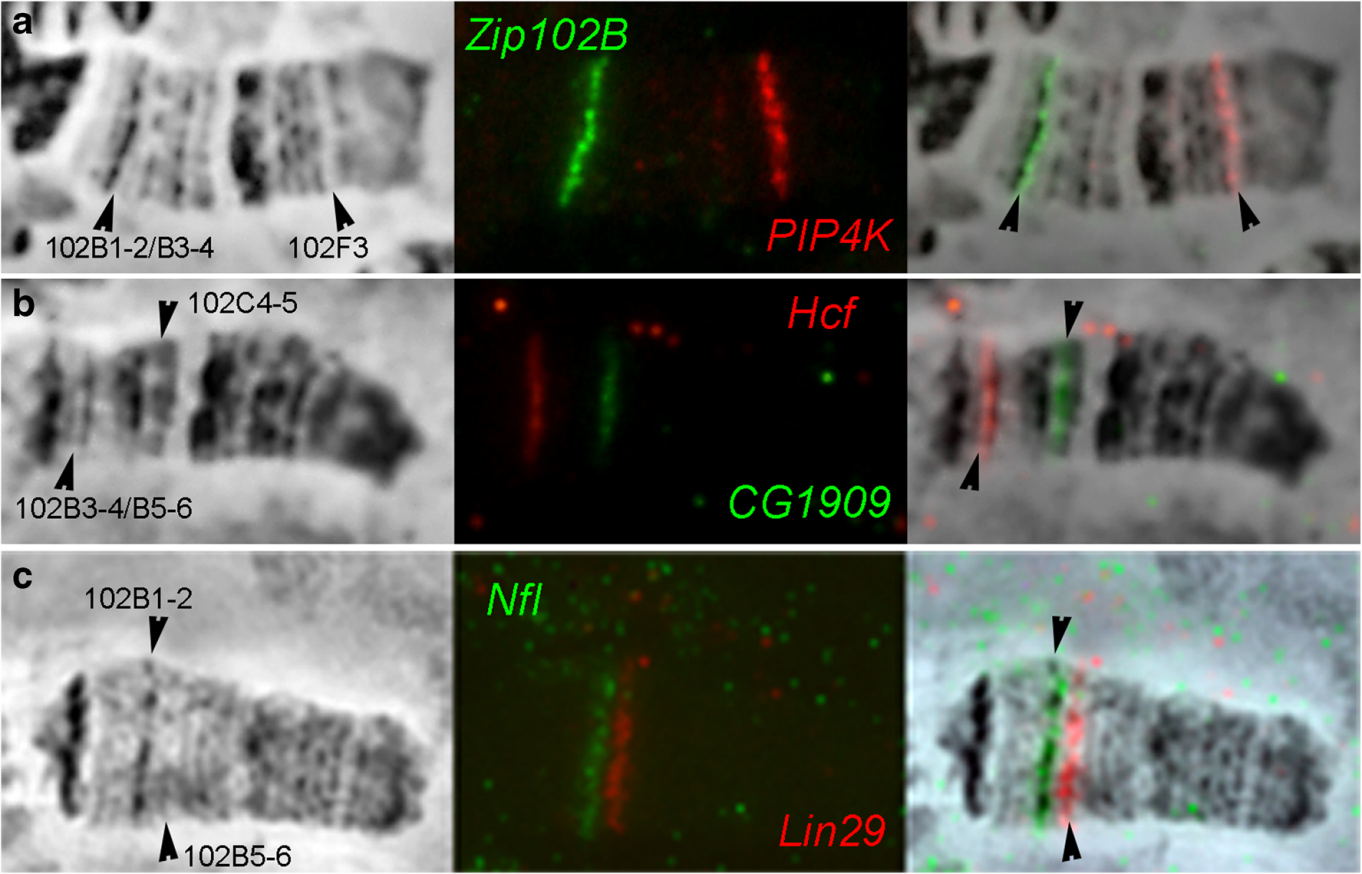
FISH on the polytene fourth chromosome. From left to right: the phase-contrast microphotograph of the fourth chromosome, combined FISH signals, and their superposition. The red signals correspond to probes labeled with TAMRA fluorochrome, the green ones correspond to probes labeled with fluorescein. The arrowheads indicate the following: **a***Zip102B* probe in the 102B1-2/B3-4 interband, *PIP4K* probe in the 102F3 band; **b***Hcf* probe in the 102B3-4/B5-6 interband, *CG1909* probe in the 102C4-5 band; **c***NfI* probe in the 102B1-2 band, *Lin29* (*Dati*) probe in the 102B5-6 band.

**Fig. 4:**
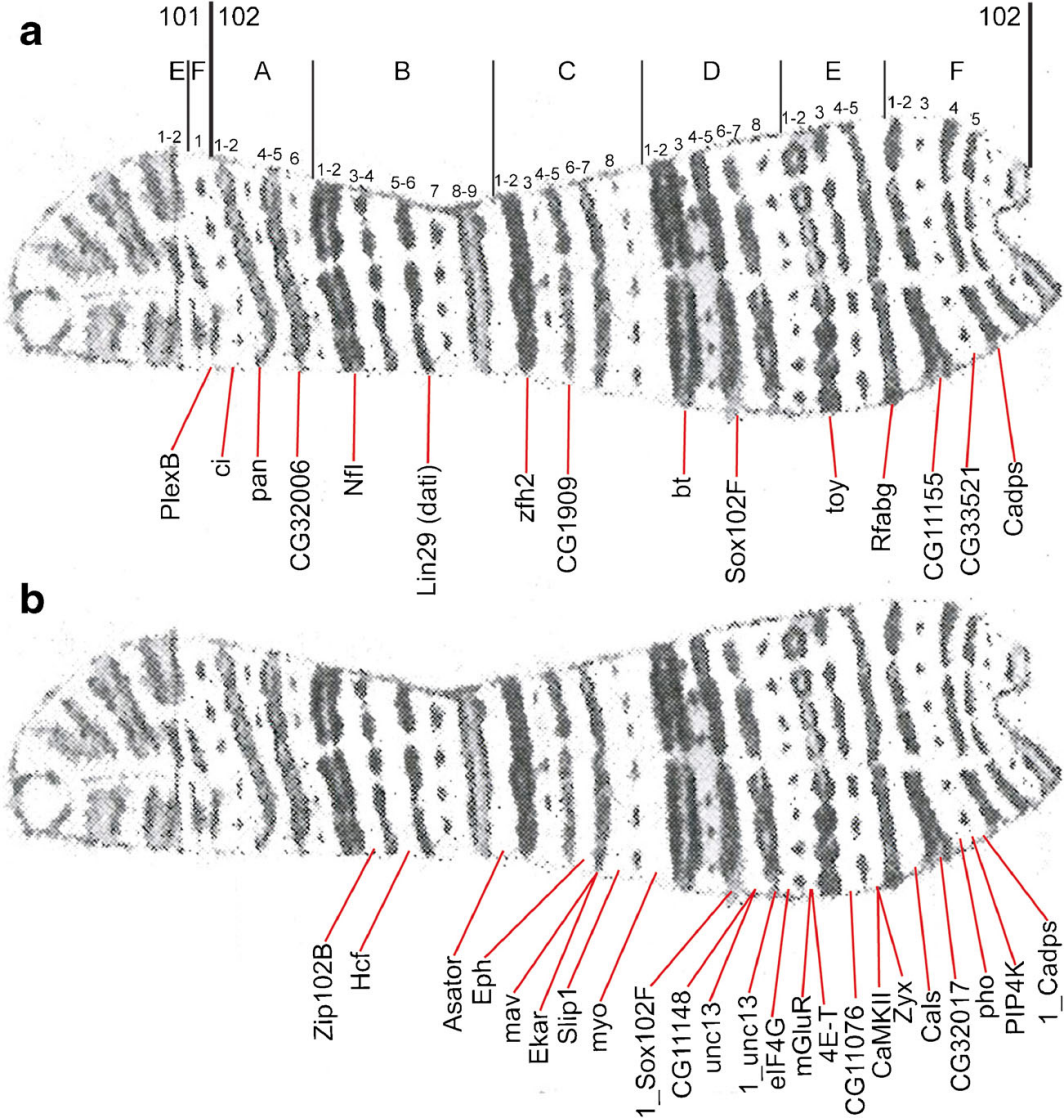
The scheme of probe localization in the polytene fourth chromosome, plotted on the map by C. Bridges (Bridges 1935a). **a** The probes from the bands predicted by the four-chromatin-state model (Zhimulev et al. 2014). **b** The probes from the interbands predicted by the four-chromatin-state model (Zhimulev et al. 2014).

In order to conduct a fine mapping of *D. melanogaster* fourth chromosome and connect all its bands and interbands with the physical map of the genome, FISH analysis of 38 DNA probes on polytene chromosomes was carried out (e.g., see Fig. 3). Fifteen of them were selected from the bands predicted by the model of four chromatin states (Zhimulev et al. 2014; Materials and methods), with ruby chromatin corresponding most closely to dense bands (Table S1, Fig. S3, Fig. S4). The initial comparison of the cytological and genomic maps was carried out using “band” samples, as they give a better resolution on FISH preparations. To clarify the boundaries of bands and interbands and to have markers of open chromatin state, 23 additional probes presumably localized in interbands were chosen (Table S2, Fig. S5, Fig. S6, Fig. S7). Mostly, two probes are presented on each preparation. Localization of one of them has already been established in this work, and another one is being mapped on this preparation. Thus, most of the morphological structures of the chromosome studied were covered with the probes. The overall probe hybridization results are plotted on the map by C. Bridges (Fig. 4). The results of these probes localization in the fourth polytene chromosome structures allowed us to relate the fourth chromosome cytological map to definite fragments of the molecular and genetic map (Table S3). A detailed description of the molecular and genetic characteristics of all the fourth chromosome morphological structures is given in the Figures S8–S16 and in the Supplementary text 2.

### Chromatin state mapping in the morphological structures of the fourth chromosome

#### Molecular characteristics of the 102В3-4–102В5-6 site

**Fig. 5:**
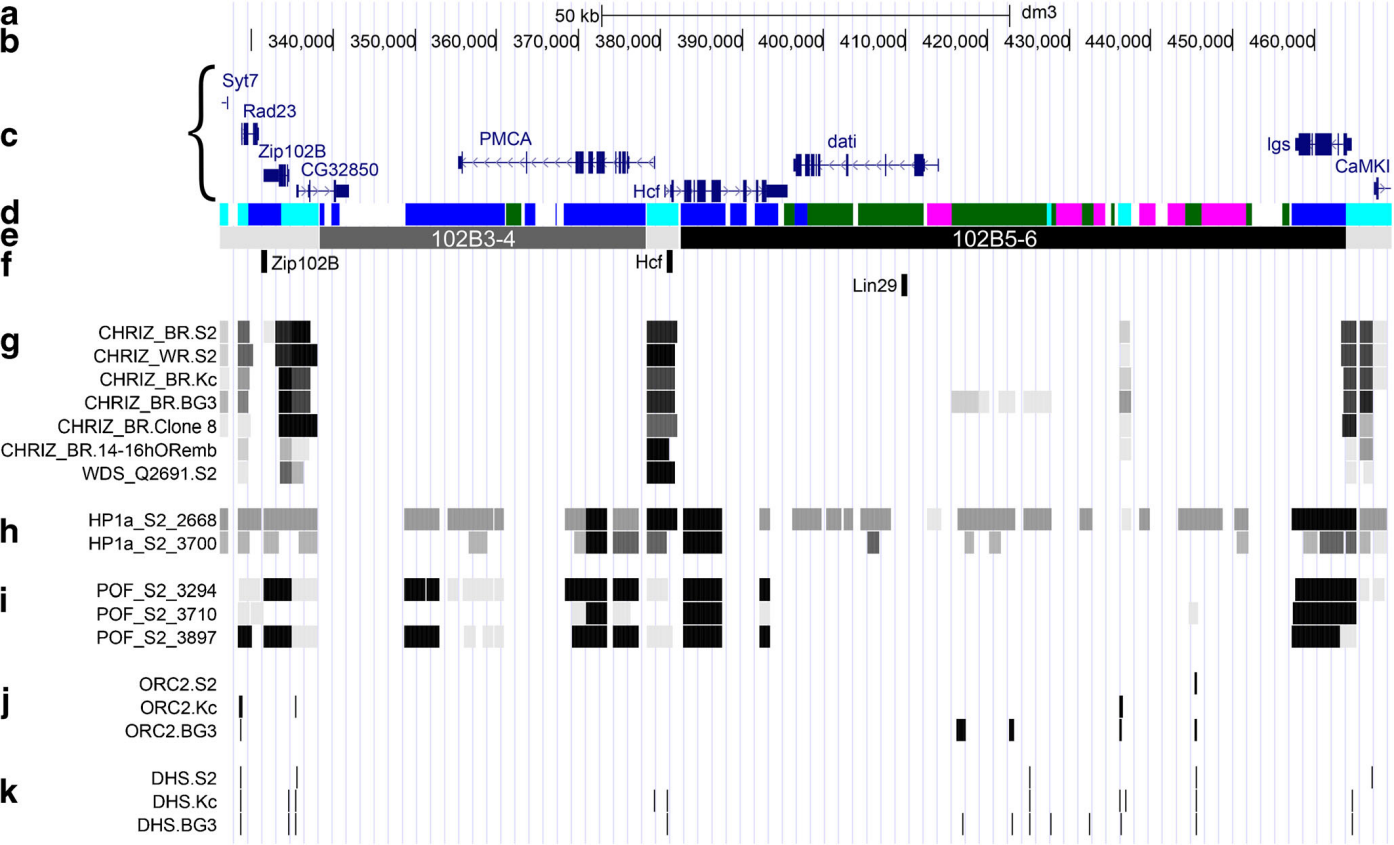
Molecular, genetic, and cytological organization of the fourth chromosome 102В3-4–102В5-6 site: **a** the scale (kb); **b** the genomic coordinates (bp); **c** the genes (denoted by a curly bracket); **d** the four-chromatin-state model; the colors correspond to the type names (Zhimulev et al. 2014; Boldyreva et al. 2017); **e** the scheme of band and interband localization relative to the genomic coordinates. The band names are denoted. The black ruby-containing bands are shown in black rectangles, the gray bands are shown in dark gray rectangles, and the interbands are shown in light gray rectangles. **f** FISH probes; **g** CHRIZ and WDS open chromatin protein localization in different cell types (modENCODE data); **h** HP1a protein localization in S2 cells (modENCODE data); **i** POF protein localization in S2 cells (modENCODE data); **j** ORC2 protein localization in different cell types (Eaton et al. 2011); **k** DNaseI hypersensitive sites (DHS) localization (Kharchenko et al. 2011)

For example, we show the 102В3-4–102В5-6 region of the fourth chromosome (Fig. 5).

The proximal part of the 102B3-4 band is confined by the open chromatin region of ~ 12.3 kb, represented equally by two aquamarine chromatin fragments surrounding a lazurite fragment. Aquamarine fragments contain the 5′ ends of the genes *Syt7*, *Rad23*, *Zip102B*, and *CG32850*, arranged “head-to-head” in pairs, and CHRIZ and WDS proteins (Fig. 5c, d, g). The size of all three fragments is so small that at the cytological level the entire area is perceived as a single 102B1-2/B3-4 interband (Fig. 5e). The size of the lazurite chromatin inclusion in the interband is ~ 4 kb, while the smallest fragment size that can form a band detected at the electron microscopy level is ~ 5 kb (Semeshin et al. 1989). The FISH probe from the *Zip102B* gene (lazurite chromatin) marks this interband (Fig. 5e–f, Fig. S5a, Fig. 4b). The 102B3-4 band (Fig. S1) is mainly represented by lazurite chromatin, its total size is ~ 40 kb, and it has a small malachite chromatin inclusion in the region of one of the PMCA introns. It is completely occupied by structural parts of *CG32850* and *PMCA* genes, with their 5′ ends located in flanking interbands (Fig. 5c–e). After the 102B3-4 band, there is the 102B3-4/B5-6 interband (Fig. S1), which is labeled with a FISH probe from the *Hcf* gene (Fig. 5e–f, Fig. S5b, Fig. 4b). On the molecular map, this interband is represented by ~ 4-kb aquamarine chromatin domain, which contains CHRIZ and WDS proteins and 5′ ends of *PMCA* and *Hcf* genes located “head-to-head” (Fig. 5c, d, g).

The large 102B5-6 band (Fig. S1) is labeled with a FISH probe from the *Lin29* gene (Fig. 5e–f, Fig. S3e, Fig. 4a). This band of ~ 81-kb size is mainly represented by dense malachite and ruby chromatin. It contains small aquamarine chromatin inclusions of 0.6 and 1.6 kb that do not contain genes or their parts and clear signals of CHRIZ and WDS proteins (Fig. 5c, d, g). It has been shown that similar inclusions of aquamarine chromatin in ruby large compact intercalary heterochromatin bands contain enhancers and insulators (Khoroshko et al. 2016). The band contains three genes, two of which start in neighboring interbands (Fig. 5c, e). Lazurite chromatin domains containing structural parts of ubiquitously active genes *Hcf* and *Igs* are attached to the band edges (Fig. 5c–e). The band size significantly increases because of combining the band middle dense area consisting of ruby and malachite chromatin, with bordering lazurite chromatin, which usually corresponds to gray bands. Such bands are described in more details in the article by Khoroshko et al. (2018). The distal edge of the 102B5-6 band is flanked by the 102B5-6/B7 interband, the aquamarine chromatin region of ~ 6 kb in size with 5′ ends of genes *Igs* and *CaMKI* located “head-to-head” (Fig. 5c–e).

It is clear that HP1a and POF proteins, specific for the fourth chromosome, are predominantly localized in chromatin types corresponding to interbands and the 102B3-4 gray band and are almost absent in the 102B5-6 black band (Fig. 5d, e, h, i). The binding sites of ORC2 (protein of the replication initiation complex) and the sites of hypersensitivity to DNase I are mainly located in interbands (Fig. 5d, e, j, k).

All other fragments of the fourth chromosome are shown on the Figures S3–S16 and described in detail in the Supplementary text 2; in general, these data correspond to the data described above.

#### Band and interband chromatin composition of chromosome 4

Based on the results of mapping, the number of bands (27 pieces, 83% of the polytenized part length) and interbands (26 pieces, 14% of the polytenized part length) of the fourth chromosome has been calculated (Fig. 2b). The band size varies from 6.6 to 89 kb with an average size of 38.41 kb. The interbands are mainly represented by aquamarine chromatin (73%; Fig. 2c). The number of dense black bands (11 pieces, 54% of the polytenized part length, Fig. 2b) containing most of the ruby chromatin (occupies 37% of black bands, Fig. 2e), and more loose gray bands (16 pieces, 29% of the polytenized part length, Fig. 2b), represented by malachite (17%) and, to a large extent, lazurite chromatin (54%, Fig. 2d), is identified. The size of black ruby bands varies from 35.8 to 89 kb with an average size of 61.04 kb. The size of gray bands varies from 6.6 to 56.8 kb with an average size of 22.86 kb. Black ruby bands are as follows: 101E1-2, 102B1-2, 102B5-6, 102C1-2, 102D1-2, 102D4-5, 102D6-7, 102E1-2, 102E4-5, 102F1-2, and 102F4,5. Gray bands are as follows: 101F1, 102A1-2, 102A4-5, 102A6, 102A6′, 102A6″, 102B3-4, 102B7, 102B8-9, 102C3, 102C4-5, 102C6-7, 102C8, 102D8, 102E3, and 102F3.

#### Exceptions in the chromatin composition of bands and interbands

A part of the interbands (8 of 26) has a complex structure according to the model of four chromatin types. In addition to aquamarine chromatin, they contain small, invisible even with electron microscopy in the form of band insertions of lazurite (seven inclusions) and malachite chromatin (two inclusions). Inclusions of lazurite chromatin correspond to structural parts of ubiquitously active genes (according to FlyBase). One of the malachite chromatin inclusions corresponds to the gene intron, and the other corresponds to the intergenic interval. The size of the lazurite inclusions varies from 2.8 to 7.2 kb, and the average value is 5.3 kb. Malachite inclusion measurements are 1.6 and 1.8 kb. All these inclusions are small in size. According to V. Semeshin, at the limit of electron microscopy resolution, in some cases, it is possible to see structures of at least 5 kb in size as individual bands (Semeshin et al. 1989).

A part of the bands (13 of 27, including 8 black bands: 102B5-6, 102C1-2, 102D1-2, 102D4-5, 102D6-7, 102E1-2, 102E4-5, 102F1-2, and 5 gray bands: 102A1-2, 102A4-5, 102C6-7, 102C8, и 102F3) has a complex structure and contains small inclusions of aquamarine chromatin according to the model of four chromatin types. In total, we found 21 inclusions, the size of which varies from 0.4 to 3.8 kb with an average value of 1.2 kb. Ten aquamarine inclusions in the bands contain 5′ ends of the genes that, basically, do not work in larval salivary glands. The exception is the fragment of aquamarine in the 102F3 band at the distal end of the fourth chromosome, which has all the features of the interband; however, we assume that, according to the map by C. Bridges, electron and light microscopy, this area contains only one gray band, 102F3. The identification of the interband-specific aquamarine state within a band by the model of four chromatin types, based on cell culture data, may be related to the genome plasticity, i.e., the presence of genes that are expressed and form an active interband in diploid tissues, but do not work in salivary gland polytene chromosomes and do not form an interband there. Five aquamarine inclusions in the bands contain intergenic spacers, and six ones contain 3′ ends of genes or their coding parts. It has been shown earlier that small aquamarine chromatin inclusions in large ruby-containing bands are enriched in enhancers and insulators (Khoroshko et al. 2016).

It was shown that a part of large black intercalary heterochromatin bands containing ruby chromatin has lazurite chromatin at the borders, which usually corresponds to gray bands containing structural parts of ubiquitously active genes. The band size is substantially increased due to the integration of the middle dense band region of ruby and malachite chromatin with bordering lazurite chromatin. Such bands are described in more detail by Khoroshko et al. (2018). In the fourth chromosome, one or both borders of 10 out of 13 ruby-containing bands are represented by lazurite chromatin.

A detailed description of all bands and interbands is provided in the Supplementary text 2.

### Genetic content of the fourth chromosome bands and interbands

**Fig. 6:**
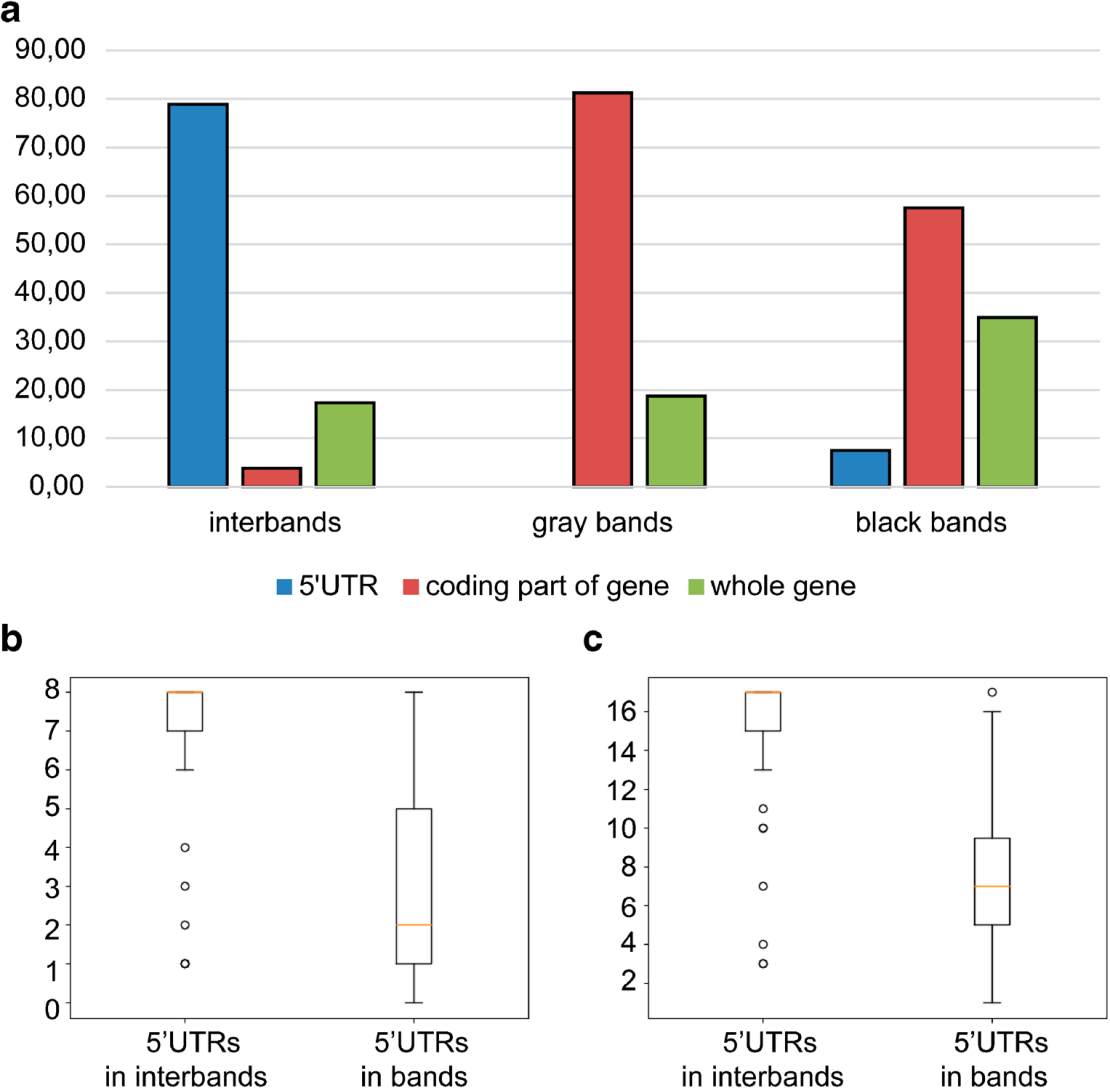
Genetic organization of the fourth chromosome domains and gene expression. **a** Enrichment of various parts of genes in the structures of the fourth chromosome. The *Y*-axis indicates the percentage (%) of each category. **b** The number of larval tissues (on the *Y*-axis) in which genes of the fourth chromosome are expressed (Chintapalli et al. 2007). **c** The number of adult fly tissues (on the *Y*-axis) in which genes of the fourth chromosome are expressed (Chintapalli et al. 2007).

We determined the genetic content of the fourth chromosome cytological structures (Table S3). It turned out that all interbands contain 5′untranslated regions of genes, with some interbands (6 pieces) containing genes completely localized in these open chromatin domains (Fig. 6a). Gray bands mainly contain the structural parts of genes with 5′regulatory regions located in flanking interbands (11 of 16 bands). The exceptions are the 102D8 band, which contains the whole gene, and the 102A4-5, 102C4-5, 102C6-7, and 102F3 polygenic bands (Table S3, Fig. 6a). Black bands are mainly polygenic (8 of 11 bands). The exceptions are the 102D6-7 band and the first (101E1-2) and the last (102F4,5) bands containing genes starting in the flanking interbands (Table S3, Fig. 6a). Black bands are enriched with coding parts of genes due to lazurite chromatin attachment to the edges of black bands. Such structures are described in more detail in the Supplementary text 2 and in the work by Khoroshko et al. (2018).

We compared the expression of genes whose 5′ regulatory regions are located in cytologically mapped interbands, with genes localized in gray and black bands of the fourth chromosome. It turned out that genes that begin in the open chromatin of interbands are on average expressed in more tissues of larvae (Fig. 6b) and adult flies (Fig. 6c) compared to genes, 5′UTRs of which are located in gray and black bands. Similar data have been obtained previously for the complete *Drosophila* genome (Zhimulev et al. 2014).

### Aquamarine and lazurite chromatin types of the fourth chromosome are enriched with POF and HP1a proteins

A statistical analysis of the differences in the distribution of proteins in the four chromatin types of the fourth chromosome and the rest of the *Drosophila* genome was performed using the Mann–Whitney *U* test. Figure S17 shows all proteins whose proportion in certain chromatin types of the fourth chromosome significantly differs from this value for the complete genome. Table 1 indicates proteins that have a significant difference between the medians of the proportions occupied by them in the corresponding type of chromatin of the fourth chromosome and the rest of the *Drosophila* genome.

**Table 1:** Enrichment and depletion of chromatin types of the fourth chromosome with proteins and histone modifications (modENCODE data, S2 cells) in comparison with the chromatin types of the rest of the *Drosophila* genome

Chromatin type	Enrichment with proteins and histone modifications in the fourth chromosome	Depletion with proteins and histone modifications in the fourth chromosome
Aquamarine	HP1a, HP2, POF	H2Bubiq, H3K4me1, H3K4me2, H3K79me2, H3K9ac, H4K16ac, H4K8ac, ISWI, MOF, MRG15, RNA-pol II, WDS
Lazurite	HP1a, HP2, POF, SU(VAR)3-9	H2AV, H2Bubiq, H3K27me1, H3K4me1, H3K4me2, H3K79me1, H3K79me2, H3K79me3, H3K9ac, H4K16ac, CHRIZ, JIL1, MRG15, RNA-pol II
Malachite	H2AV, HP1a	
Ruby	H3K9me2, SU(VAR)3-9	

Aquamarine and lazurite active chromatin types of the fourth chromosome that correspond to the regulatory regions and the bodies of housekeeping genes in the *Drosophila* genome, the interbands and loose gray bands respectively (Zhimulev et al. 2014), are enriched with POF protein unique for this chromosome (Larsson et al. 2001). In addition, these two types of chromatin are enriched with the heterochromatin protein HP1a, and the HP2 protein colocalized with it. Lazurite chromatin of the fourth chromosome relative to the genome lazurite chromatin is enriched with the SU(VAR)3-9 histone methyltransferase, which marks inactive chromatin with H3K9 methylation. Aquamarine and lazurite chromatin of the fourth chromosome are depleted with a large number of histone modifications associated with active transcription, and with open chromatin proteins. Aquamarine chromatin of the fourth chromosome relative to the genome aquamarine chromatin is also depleted with the ISWI remodeling protein associated with a higher order chromatin structure involving H1 histone (Deuring et al. 2000; Corona et al. 2007).

Malachite chromatin of the fourth chromosome in comparison with the malachite chromatin of the rest of the genome is enriched with the heterochromatin protein HP1a and the histone variant H2AV. The phosphorylated form of H2AV (γH2AV) is associated with double-stranded DNA breaks, possibly caused by underreplication (Madigan et al. 2002; Mehrotra and McKim 2006; Andreyeva et al. 2008). As it has been shown earlier, this chromatin type mostly corresponds to intergenic intervals and introns of genes, and also it is a part of active gene long introns forming thin gray bands of polytene chromosomes (Zhimulev et al. 2016; Boldyreva et al. 2017).

Transcriptionally inactive ruby chromatin of the fourth chromosome is enriched with H3K9me2 and SU(VAR)3-9 marks of repressed chromatin relative to the ruby chromatin of the rest of the genome.

### Distribution of ORC2, HP1a, POF, CHRIZ, H3K27me3, and SUUR proteins in the fourth chromosome of *D. melanogaster*

**Fig. 7:**
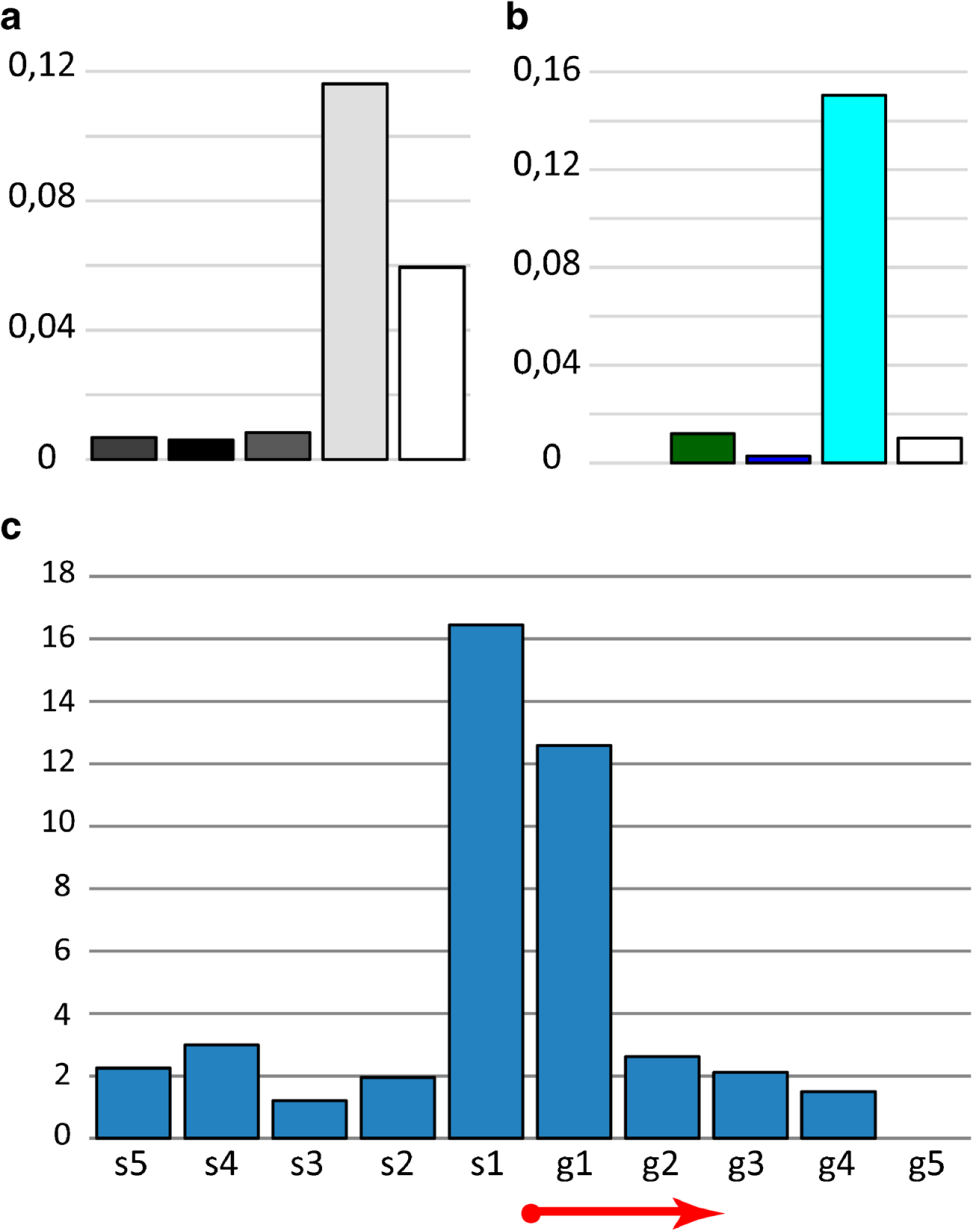
ORC2 protein distribution (modENCODE data): **a** in the cytological structures of the fourth chromosome, with the first column corresponding to all the bands of the fourth chromosome, and further — as shown in Fig. 2b; **b** in the four chromatin types of the fourth chromosome. The ordinate shows the density in pcs/kb. **с** ORC2 protein distribution (modENCODE data) in S2 cells relative to genes beginning in the interbands of the fourth chromosome. The interbands are divided into fragments of 200 bp from the beginning of the gene towards the intergenic spacer (s1–s5) and towards the structural part of the gene (g1–g5); the genes are aligned with respect to the beginning. The graph shows five fragments in each direction. The ordinate shows the total number of sites in these fragments, normalized by the number of the gene transcripts. The red arrow shows the start and direction of the genes.

We studied the distribution of the replication complex protein ORC2 (modENCODE data). The results suggest that the ORC2 binding sites in the fourth chromosome are mainly localized in aquamarine chromatin, which corresponds to the interbands (Fig. 7a, b). This is in good agreement with the whole genome data (Zhimulev et al. 2014). The ORC2 distribution density in each morphological structure of the fourth chromosome is shown in Figure S18. A more detailed analysis showed that ORC2 in the fourth chromosome has a binding peak within 400 bp around the beginning of genes, whose 5′UTRs lie in the interbands (Fig. 7c).

**Fig. 8:**
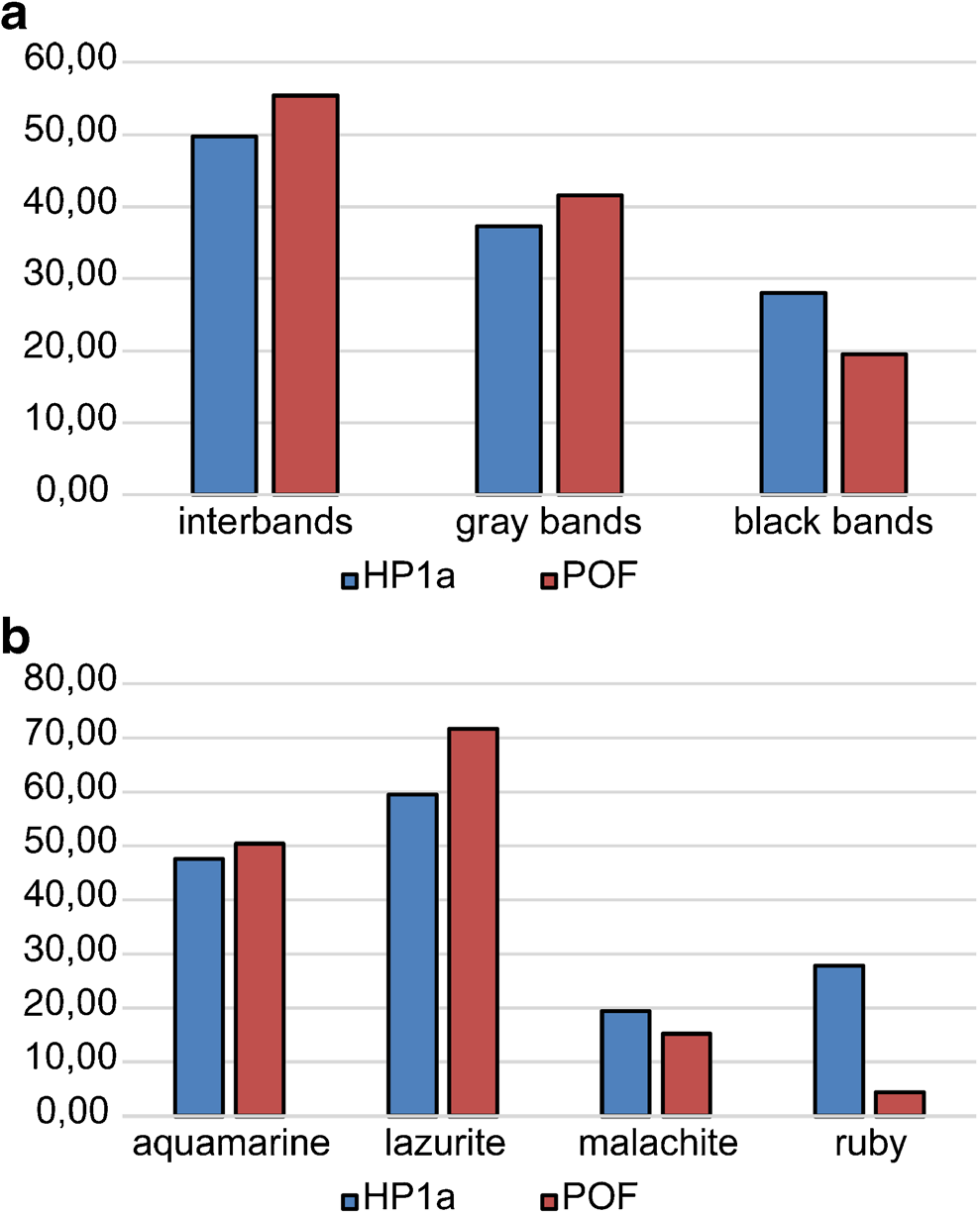
Distribution of HP1a and POF proteins (modENCODE data) in the fourth chromosome in S2 cells. The diagram on the *Y*-axis shows the proportions (%) of the total length of morphological structures (**a**) and chromatin domains (**b**) occupied by HP1a and POF.

The distribution of HP1a and POF proteins (modENCODE data), which are responsible for the special epigenetic status of the fourth chromosome, was studied in more detail. Analysis of the chromatin immunoprecipitation data on the studied proteins in S2 cells showed that they are located predominantly in domains corresponding to interbands and gray bands of the fourth chromosome (Fig. 8a). Relative to the model of four chromatin types, these proteins are localized in lazurite chromatin (Fig. 8b), which corresponds to the coding parts of genes (Fig. 6a) and, generally, gray bands (Fig. 2d), and in aquamarine chromatin (Fig. 8b), which corresponds to promoters of ubiquitously active genes (Fig. 6a) and interbands (Fig. 2c).

**Fig. 9:**
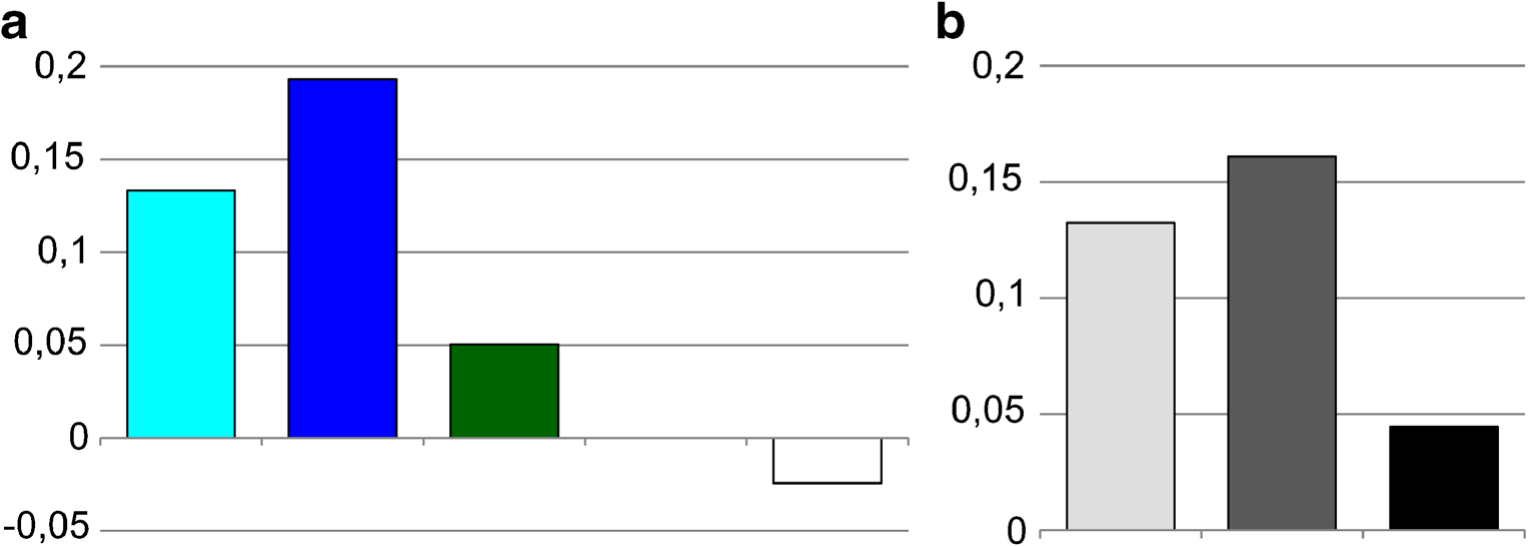
Distribution of POF protein in the fourth chromosome in the salivary gland cells of *Drosophila* larvae (Lundberg et al. 2013a; Johansson and Larsson 2014) **a** relative to the bands and interbands and **b** relative to the chromatin domains of four types (Zhimulev et al. 2014; Materials and methods). The value of the median of the ChIP profile peaks for each type of domains is shown on the *Y*-axis.

In addition, we studied the distribution of POF protein in salivary gland cells of *Drosophila* larvae. The analysis of chromatin immunoprecipitation results of this protein specific for the fourth chromosome (Lundberg et al. 2013a; Johansson and Larsson 2014) showed that, as in S2 cells, POF predominantly localizes in lazurite and aquamarine chromatin types (Fig. 9a) and in the corresponding gray bands and interbands of the fourth chromosome (Fig. 9b). POF is completely absent in the highly compact ruby chromatin. Localization of POF in black bands is explained by the fact that these bands in the fourth chromosome contain not only ruby chromatin but also a rather large proportion of malachite and lazurite chromatin (Fig. 2e).

**Fig. 10:**
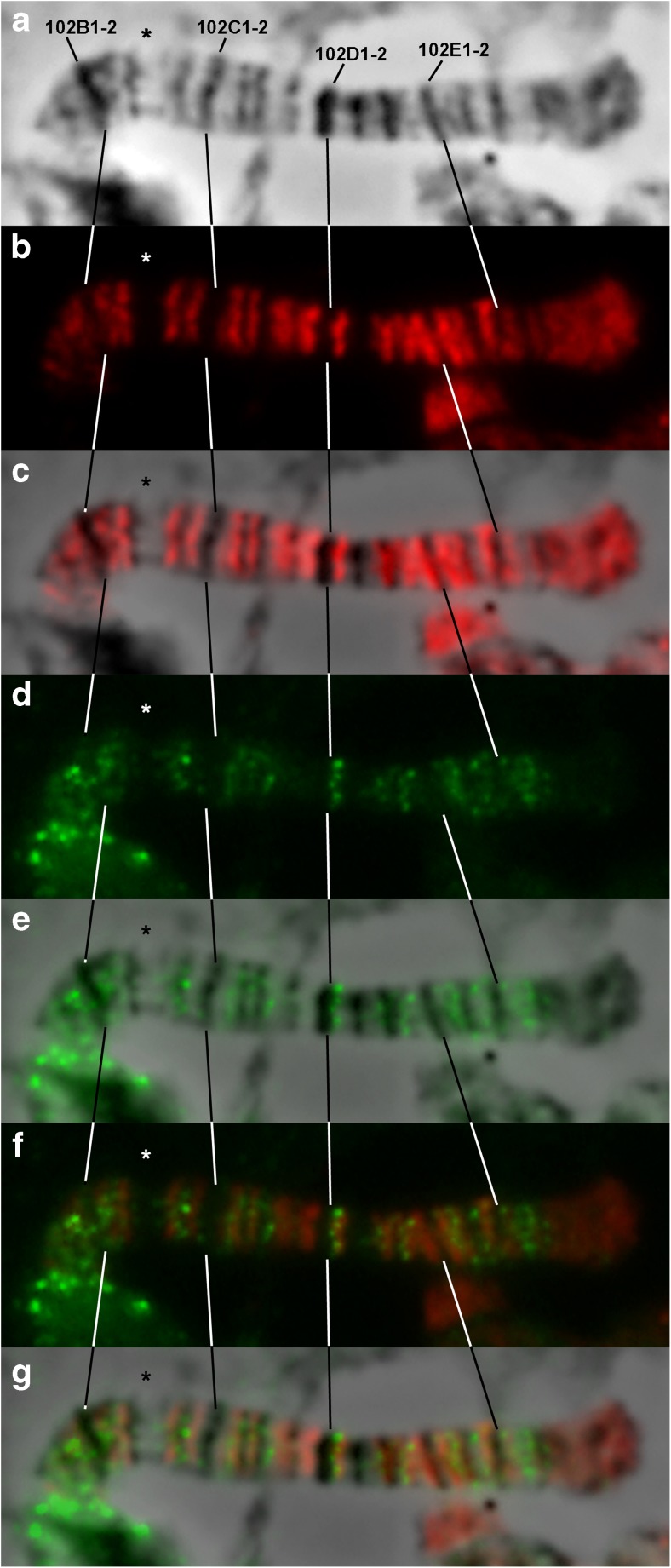
POF and CHRIZ localization on the polytene fourth chromosome of *D. melanogaster*. **a** Phase contrast image of the fourth chromosome. **b** CHRIZ staining. Antibodies are the same as in Gortchakov et al. (2005). **c** Merged phase contrast and CHRIZ staining. **d** POF staining. Antibodies are the same as in Larsson et al. (2001). **e** Merged phase contrast and POF staining. **f** Merged CHRIZ and POF staining. **g** Merged phase contrast, CHRIZ, and POF staining. The arrow indicates the 102B5-6/B7 interband where no POF and CHRIZ binding was observed.

Thus, protein POF is localized in some sites of open chromatin of the fourth chromosome. Another open chromatin protein is CHRIZ, which specifically decorates the interbands of polytene chromosomes (Gortchakov et al. 2005). We aimed to find out how are these proteins located relative to each other. To achieve this goal, we performed co-immunostaining of *Drosophila* polytene chromosomes with POF and CHRIZ antibodies (Fig. 10). We obtained a very stretched well-spread polytene fourth chromosome using *SuUR*^*ES*^*Su(var)3-9*^*06*^ double mutant flies with suppressed underreplication (Fig. 10a). The protein CHRIZ was expected to occupy all interbands of the fourth chromosome, but it appeared to occupy almost all of them except one (Fig. 10b, c). The 102B5-6/B7 interband remains empty (marked with an asterisk in Fig. 10). In addition, CHRIZ antibodies also paint the telomeric loose gray tip of the fourth chromosome. The protein POF is localized in some interbands and gray bands of the fourth chromosome, and never in black bands (17 signals in total; Fig. 10d, e). Interestingly, the 102B5-6/B7 interband also lacks POF binding. However, this issue is yet to be addressed. The protein POF only partially colocalizes with CHRIZ (Fig. 10f, g). Both of the studied proteins are absent from pericentric part of the fourth chromosome. Thus, at the cytological level, the proteins POF and CHRIZ occupy open chromatin sites of the fourth chromosome and only partially colocalize. This observation is supported by the molecular data, since it has been shown that POF specifically binds to genes with a strong exon preference (Johansson et al. 2007b), and the exons of active genes correspond to lazurite chromatin at the whole genome level (Zykova et al. 2018), whereas CHRIZ is generally associated with the promoters of housekeeping genes in aquamarine chromatin (Zhimulev et al. 2014; Zykova et al. 2018).

**Fig. 11:**
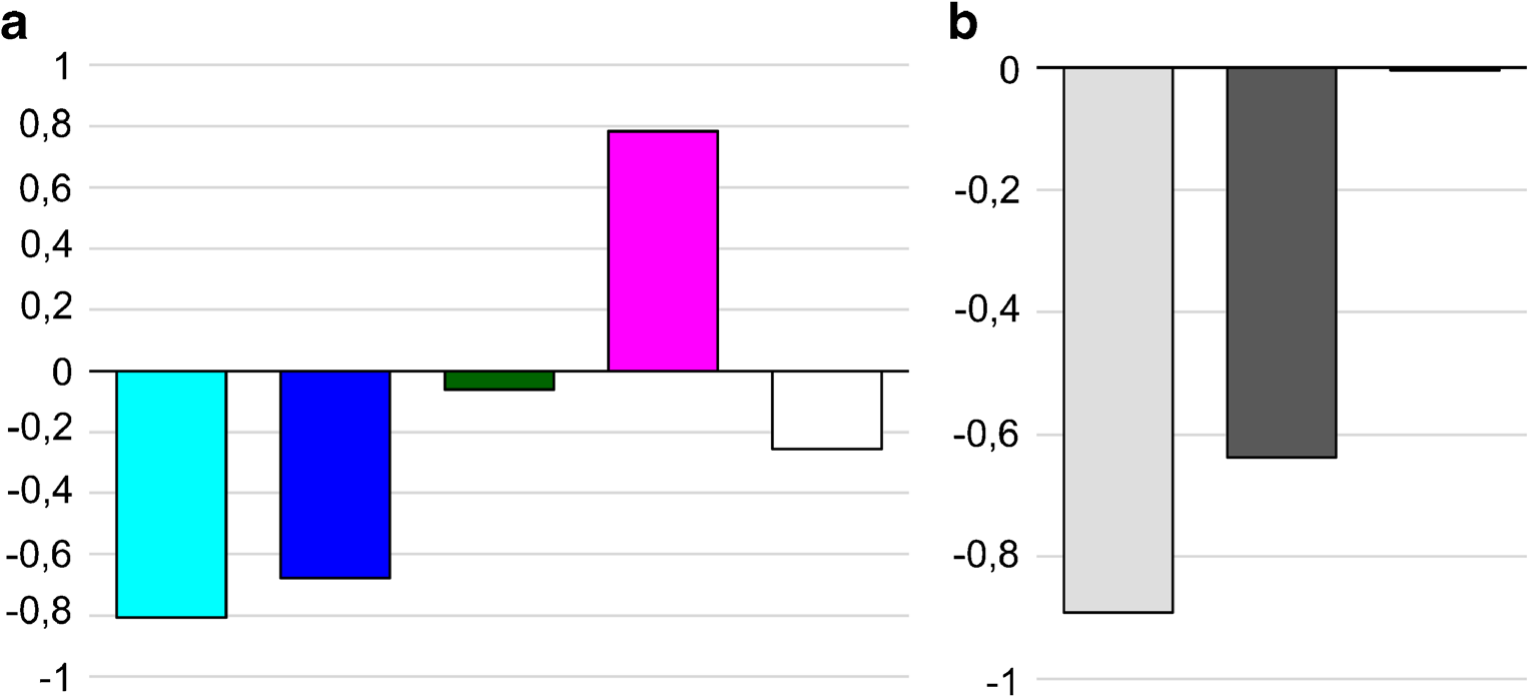
The distribution of H3K27me3 in the *Drosophila* larvae salivary gland cells (Sher et al. 2012). **a** in the four chromatin types of the fourth chromosome, and **b** in the bands and interbands of the fourth chromosome. The median value of the distribution profile peaks is shown on the *Y*-axis

Trimethylated histone H3 at the 27th lysine is the epigenetic mark of repressed chromatin. It is associated with Polycomb-dependent heterochromatin. We analyzed the distribution of this H3K27 modification obtained in the DamID experiment in *Drosophila* salivary gland cells (Sher et al. 2012; Fig. S19i) relative to the model of four chromatin types (Zhimulev et al. 2014; Materials and methods) and in the morphological structures of the fourth chromosome. It turned out that H3K27me3 is enriched only in dense ruby chromatin (Fig. 11a), which generally corresponds to black bands. When considering the distribution of this modification in the morphological structures, it is shown that in black bands, the median value is maximal in comparison with the remaining morphological structures; however, it is about 0 (Fig. 11b). This is due to the fact that ruby chromatin is only about a third of the total length of black bands, and the median value of H3K27me3 enrichment is affected by all other types of chromatin in the composition of black bands (Fig. 2e).

**Fig. 12:**
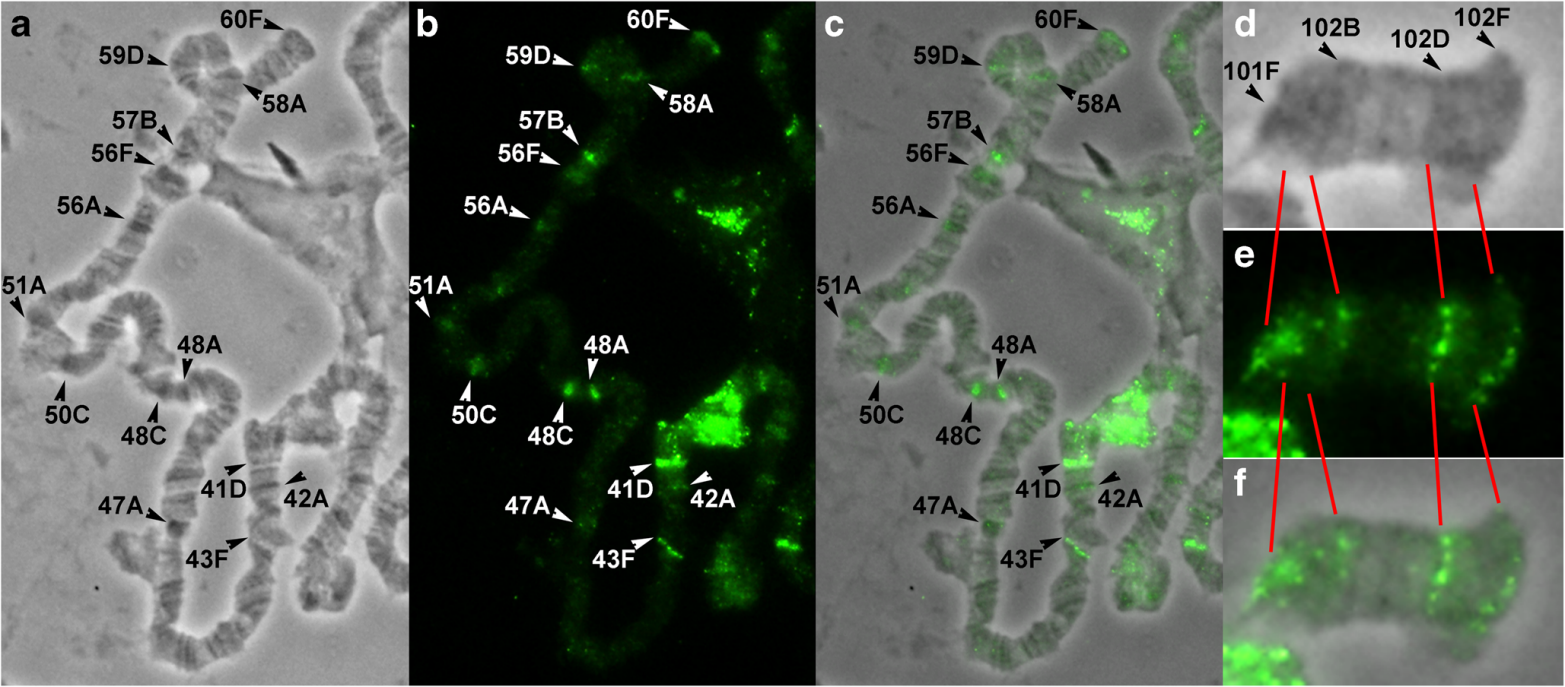
SUUR localization in polytene chromosomes. The picture is kindly provided by T. D. Kolesnikova. **a** Phase contrast of the 2R chromosome. **b** SUUR localization in 2R chromosome. **c** Merged image of phase contrast and SUUR localization in the 2R chromosome as control of proper SUUR localization in the dot chromosome. **d** Phase contrast of the fourth chromosome. **e** SUUR localization in the fourth chromosome **f** merged image of phase contrast and SUUR localization in the fourth chromosome

Analyzing the protein composition of the fourth chromosome, we found that the data on the distribution of the Supressor of Underreplication protein (SUUR) in the fourth chromosome of *D. melanogaster*, obtained earlier by using the DamID method in larval salivary glands (Maksimov et al. 2014; Posukh et al. 2017), in cell cultures (Filion et al. 2010; Maksimov et al. 2014), embryos, and brain (Maksimov et al. 2014) do not correspond to the immunolocalization data on polytene chromosomes (Fig. S19k-p, Fig. 12). The data on salivary gland chromosomes (Posukh et al. 2017) reveal almost continuous signal of SUUR binding on the fourth chromosome (Fig. S19l). Although SUUR binding in *Drosophila* chromosomes is dynamic and varies depending on the stage of replication (Kolesnikova et al. 2013), none of the stages shows continuous painting of the fourth polytene chromosome. The immunostaining reveals up to four discrete signals of this protein binding: near the centromere and telomere regions, and two bands in 102B and 102D regions (Fig. 12), as it has been reported earlier (Zhimulev et al. 2003).

### Specificity of mobile element distribution in the fourth chromosome

#### *P*–element distribution

**Fig. 13:**
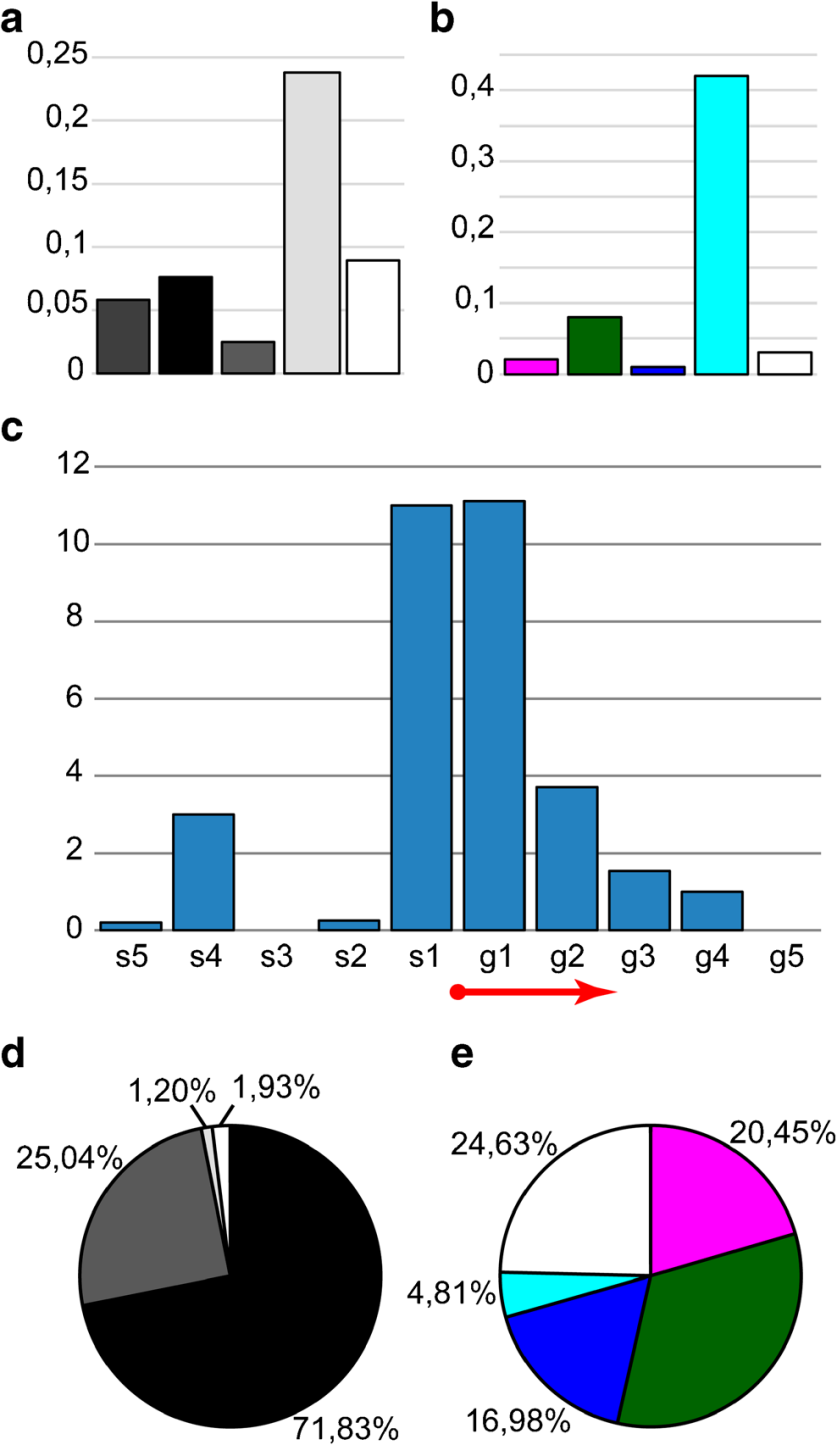
Mobile element distribution in the fourth chromosome. *Р–*element distribution: **a** in the cytological structures of the fourth chromosome, with the first column corresponding to all the bands of the fourth chromosome, and further—as shown in Fig. 2b; **b** in the four chromatin types of the fourth chromosome. The ordinate shows the density in pcs/kb. **с***Р*-element distribution relative to genes beginning in the interbands of the fourth chromosome. The interbands are divided into fragments of 200 bp from the beginning of the gene towards the intergenic spacer (s1–s5) and towards the structural part of the gene (g1–g5); the genes are aligned with respect to the beginning. The graph shows five fragments in each direction. The ordinate shows the total number of sites in these fragments, normalized by the number of the gene transcripts. The red arrow shows the start and direction of the genes. The distribution of the *1360* element: **d** in the bands and interbands; **e** in the four chromatin types of the fourth chromosome.

The distribution of *P*-transposon insertions into morphological structures and various chromatin types of the fourth chromosome was studied (107 insertions in total). These mobile genetic elements are distributed not randomly. In the fourth chromosome, *P–* elements are predominantly embedded in interbands and aquamarine chromatin maximally corresponding to these structures (Fig. 13a, b) as it has previously been shown for the whole *Drosophila* genome (Zhimulev et al. 2014). The density of the insertions in each structure of the fourth chromosome is shown in Figure S20. A more detailed analysis showed that the maximum number of *P*-transposons in the fourth chromosome is located within 400 bp around the 5′UTRs of genes in interbands and aquamarine chromatin corresponding to them (Fig. 13c).

#### *1360* distribution

In addition, the specificity of the *1360* element distribution in the fourth chromosome of *D. melanogaster* was determined. The *1360* element exhibits strain-specific location in the euchromatic parts of the chromosomes, but constant heavy labeling of the 12E1-2, 42B1-3, 52A1-2, 62A1-2, 75B, and 82C1-3 regions, chromosome bases, the chromocenter, and numerous sites of the fourth chromosome (Kholodilov et al. 1988). Sequence analysis revealed 37-bp terminal inverted repeats, flanked by 6-bp direct repeats, and two inverted repeats of 15 bp in the central part, capable of hairpin formation. This fragment called “element *1360*” has high A-T content and significant number of short nucleotide tracts. It has sequences with homology to the *FB–* and *P–*elements (as a rule, poly(A) and poly(T) tracts, characteristic of these transposable elements). The *1360* element belongs to the *Hoppel* family (Reiss et al. 2003) and is a particular class of these dispersed and deleted sequences (Kholodilov et al. 1988; Kurenova et al. 1990). The *1360* element is entirely encompassed within the 3.4-kb *Hoppel* element (Reiss et al. 2003).

In total, there are 59 remnants of the *1360* element (modENCODE data) in the fourth chromosome; four of them were excluded from the analysis, since they completely overlapped with other remnants. The total length of *1360* remnants in the *Drosophila* fourth chromosome is 49,974 nucleotides. It is shown that the *1360* element is distributed in the fourth chromosome of *Drosophila* not in a random manner. In contrast to *P–*elements that tend to integrate into open chromatin, *1360* element is predominantly located in the fourth chromosome bands and ruby, malachite, and lazurite chromatin corresponding to bands. The distribution of *1360* element in the cytological structures and different types of chromatin of the fourth chromosome is shown (Fig. 13d, e).

### Distribution of the DNaseI hypersensitivity sites

**Fig. 14:**
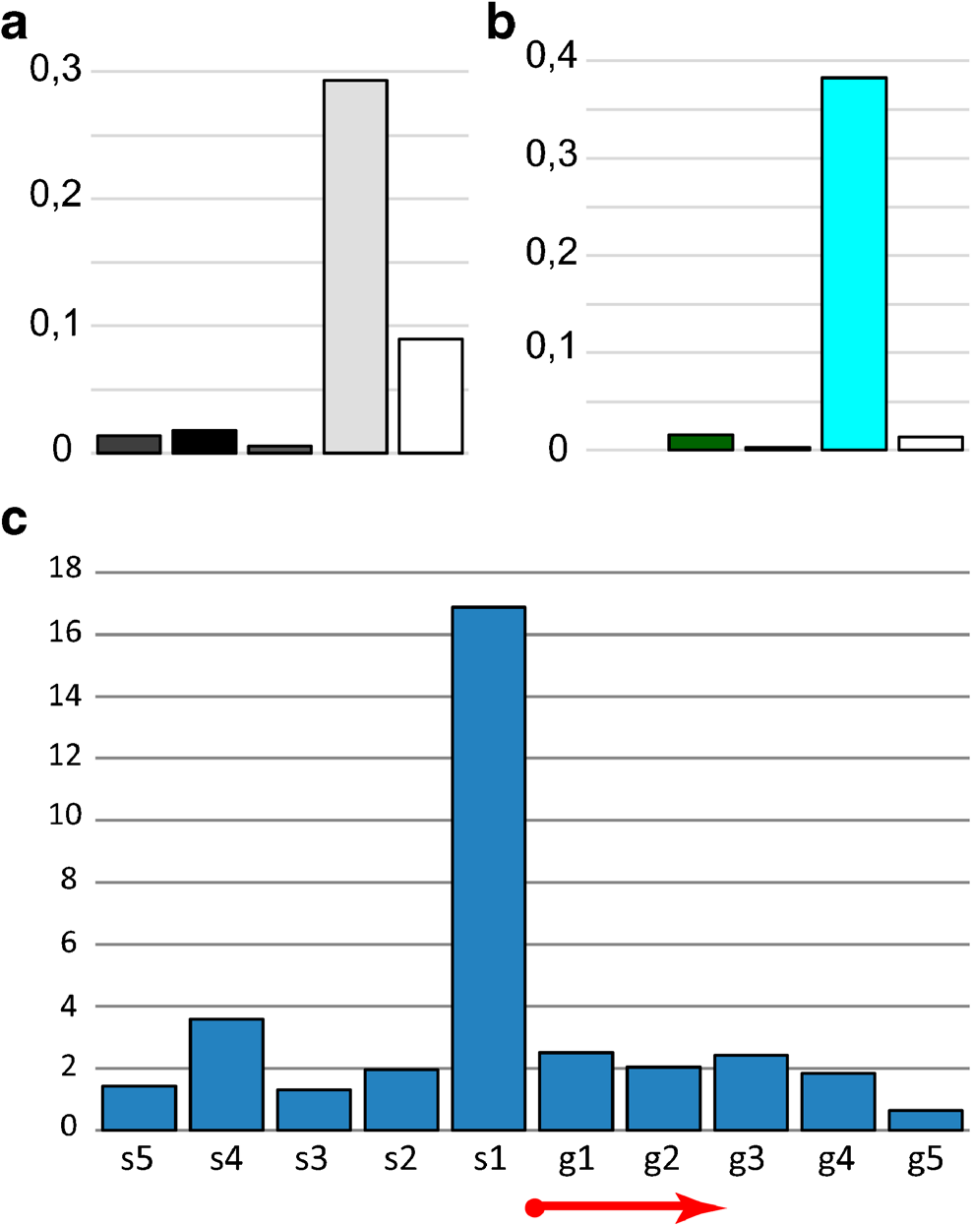
Distribution of the DNaseI hypersensitivity sites (DHS): **a** in the cytological structures of the fourth chromosome, with the first column corresponding to all the bands of the fourth chromosome, and further—as shown in Fig. 2b; **b** in the four chromatin types of the fourth chromosome. The ordinate shows the density in pcs/kb. **с** DHS distribution (modENCODE data) in S2 cells relative to genes beginning in the interbands of the fourth chromosome. The interbands are divided into fragments of 200 bp from the beginning of the gene towards the intergenic spacer (s1–s5) and towards the structural part of the gene (g1–g5); the genes are aligned with respect to the beginning. The graph shows five fragments in each direction. The ordinate shows the total number of sites in these fragments, normalized by the number of the gene transcripts. The red arrow shows the start and direction of the genes.

We show that the DHS in the fourth chromosome predominantly localize in aquamarine chromatin corresponding to interbands, which agrees with the data obtained for the rest chromosomes (Fig. 14a, b; Zhimulev et al. 2014). The density of DHS in each morphological structure of the fourth chromosome is shown (Fig. S21). A more detailed analysis showed that DHS in the fourth chromosome are predominantly located within 200 bp upstream the 5′UTRs of genes in the interbands (Fig. 14c).

## Discussion

The correlation of the *Drosophila* genome physical map and the large amount of data on gene localization, histone modifications, chromatin proteins, and various regulatory sites with detailed cytological maps of polytene chromosomes reflecting the structural organization of the genome in the interphase nucleus allows us to understand the patterns of genome functioning and their relationship to chromosome morphology. This work was devoted to such investigation of the *Drosophila* fourth chromosome.

The fourth chromosome was chosen as the object of study, since it has a number of unique properties. It combines the properties of eu- and heterochromatin. High density of active genes corresponding to euchromatin is mixed with the absence of recombination and, as a consequence, high density of repeated DNA. In particular, the fourth chromosome comprises the *1360* element, which, in addition, is present only in several genome sites (Kholodilov et al. 1988). Chromatin of the fourth chromosome is characterized by H3K9 methylation and a typical heterochromatin protein HP1 recognizing this epigenetic mark. Herewith, methylation of H3K9 is introduced by dSETDB1, histone methyltransferase specific for this chromosome. Moreover, POF protein specifically stains the fourth chromosome on squashed preparations. In addition, small size, a large number of gray bands, and frequent ectopic contacts of its distal tip with the chromocenter complicate microscopic analysis of the fourth polytene chromosome, so at this time there is no detailed, universally recognized cytological map.

In this work, we refined the cytological map of the fourth chromosome and superposed its bands and interbands with the genomic coordinates using the FISH analysis and the four-chromatin-state model (Zhimulev et al. 2014; Materials and methods). This allowed us to determine some molecular characteristics of the cytological structures of the studied chromosome using open access databases.

The unique feature of the four-chromatin-state model is that it reveals the aquamarine chromatin type that is enriched with interband-specific proteins, and it is this chromatin type which allows matching cytological and molecular chromosome maps (Zhimulev et al. 2014; Boldyreva et al. 2017). The fact is that the interbands correspond to the boundaries of topologically associated domains (Stadler et al. 2017). Other chromatin state models that yield epigenetic domains based on clustering chromatin proteins do not distinguish a separate “interband” chromatin type (Kolesnikova 2018; Kolesnikova et al. 2018). Thus, the four-chromatin-state model designates the boundaries of physical but not epigenetic domains that not always coincide (Hou et al. 2012).

We have shown that in the fourth chromosome the portion of dense ruby chromatin corresponding to black polygenic bands is 21% (Fig. 1a), which is about two times smaller than the ruby portion in the full genome of *Drosophila* (Zhimulev et al. 2014). The fraction of open aquamarine chromatin in the fourth chromosome is 12% (Fig. 1a), and it basically corresponds to interbands (Fig. 1c) and contains 5′UTRs of housekeeping genes (Fig. 6a, Table S3), as in the whole genome. Coding parts of these genes are embedded in gray bands (Fig. 6a, Table S3), largely consisting of lazurite and malachite chromatin (Fig. 2d). These two types of chromatin at the genome level are supposed to be of intermediate compaction, since they are moderately enriched in certain molecular characteristics of both active and repressive chromatin (Boldyreva et al. 2017). In addition, malachite chromatin located at the borders of the intercalary heterochromatin bands forms a transition zone between these compact black bands and more loose areas (Khoroshko et al. 2016).

The gaps (areas where the model does not output certain chromatin type) in the fourth chromosome occupy more than 20% (Fig. 1a), whereas in the whole genome, these areas of uncertainty are only about 5% (Boldyreva et al. 2017). Perhaps this is due to the fact that the protein distribution data has a smaller coverage for the fourth chromosome.

We showed that, according to the whole genome project data, four chromatin types of the fourth chromosome are enriched in marks of closed chromatin associated with gene repression, and depleted in many proteins and histone modifications of open chromatin relative to the rest of the genome (Table 1). Among other things, relative to the rest of the genome, aquamarine and lazurite chromatin of the fourth chromosome are enriched in the POF protein unique for this chromosome, and the heterochromatin protein HP1a (Table 1).

Previously, it has been shown that HP1a protein is localized not only in such typical heterochromatin regions as telomeres and pericentric heterochromatin, but also in some euchromatic loci and in the fourth chromosome of *Drosophila*, and that in the fourth polytene chromosome, this protein shows banded pattern (James et al. 1989). POF localization in the fourth chromosome has been shown to be approximately complementary to DAPI bands, i.e., it predominantly corresponds to interbands, and it is absent in the centromere of this chromosome (Larsson et al. 2001). Using chromatin immunoprecipitation, it has been shown that POF binds primarily to genes (Johansson et al. 2007a). In the same paper, it has been demonstrated that POF and HP1 are colocalized at the cytological level. A finer study using high-resolution ChIP experiment has revealed the distribution profiles of these two proteins. This study has shown that POF specifically binds to genes with a strong exon preference, and HP1a has a similar profile with a higher basal level of enrichment in the intergenic spacers and an additional peak in the promoter regions of occupied genes (Johansson et al. 2007b). The presence of the RNA-binding domain in the POF protein agrees with the RNA-seq data that indicate the cotranscriptional binding of this protein to the fourth chromosome nascent RNA and spliced RNA (Johansson et al. 2012). Improved polytene chromosome staining technique has revealed three additional POF binding sites: 2L:31 in in both males and females (Lundberg et al. 2013b), and PoX1 and PoX2 on the X chromosome in females (Lundberg et al. 2013a). Whole genome data on POF distribution in salivary gland polytene chromosomes obtained by ChIP-chip experiment demonstrate these three peaks (Lundberg et al. 2013a; Johansson and Larsson 2014). Recently, an optimal autonomous POF target has been reported to comprise a gene and a block of X chromosome-linked satellites 1.688 downstream this gene; thereby, the fourth chromosome seems to bear a cooperative effect of a large number of suboptimal combination of genes with repeats (Kim et al. 2018).

For a long time, HP1a has been considered as a heterochromatin protein (James and Elgin 1986). However, it plays an unusual role in the functioning of the fourth chromosome chromatin. On the one hand, this protein is necessary for the repression of transposons that are inserted in the fourth chromosome, and mutations in the *Su(var)2-5* gene lead to loss of silencing of the reporter gene in such construction (Sun et al. 2000; Riddle and Elgin 2006). On the other hand, it has been shown that HP1a binds to promoters and within the bodies of the active genes of the fourth chromosome (Johansson et al. 2007b; Figueiredo et al. 2012). HP1a is an indispensable factor for maintaining open chromatin state in promoter regions of the dot chromosome genes (Cryderman et al. 2011). Gene promoters usually located in repressive environment are held in open conformation if only they are packaged in HP1a-containing chromatin, and the transcription of such genes is not canceled by H3K9me2 (Cryderman et al. 2011). It has been shown that there are two mechanisms for HP1a attracting to the fourth chromosome: POF-dependent recruiting of HP1a in the active gene areas, and POF-independent binding mechanism in the silent gene domains and clusters of repeats (Riddle et al. 2012). The specific high enrichment of actively transcribed gene coding parts in HP1a on the fourth chromosome positively regulates gene expression, promoting the transcription elongation and suppressing RNA polymerase pausing at the 5′ ends of genes (Riddle et al. 2012).

Linking of the cytological map of the *D. melanogaster* fourth polytene chromosome to the genomic coordinates enabled us to clarify the localization of HP1a and POF proteins with respect to the bands and interbands of the chromosome studied. Our data show that these proteins are mainly located in the interbands and gray bands of the dot chromosome (Figs. 8 and 9). According to the model of four chromatin states (Zhimulev et al. 2014; current investigation), HP1a and POF are most abundant in lazurite and aquamarine chromatin, which correspond to gray bands and interbands, respectively (Figs. 8 and 9). This result agrees with the previously published data that POF and HP1a are colocalized with the fourth chromosome genes, and the more active the transcription is, the stronger the binding is (Johansson et al. 2007b), since aquamarine chromatin corresponds to the promoters of ubiquitously active genes, and lazurite chromatin corresponds to the coding parts of these genes (Fig. 6a).

Recently, Kolesnikova and coworkers have determined the genomic coordinates for all black ruby bands of polytene chromosome 2R and have shown that the distribution of repressive chromatin marks H3K27me3 and SUUR is a good criterion for determining the boundaries of black bands (Kolesnikova et al. 2018). Using ChIP data on H3K27me3 localization in *Drosophila* larval salivary glands published earlier (Sher et al. 2012; GEO: GSE31897; Fig. S19i), we determined that in the fourth chromosome, only black bands containing large blocks of ruby chromatin are enriched with H3K27me3 (Fig. 11; Fig. S19c, d, i). The only exception is the first high peak of H3K27me3 in the interval between black bands proximal to the centromere (Fig. S19c, d, i). As for SUUR protein, we faced with a contradiction between the DamID data published earlier (Filion et al. 2010; Maksimov et al. 2014; Posukh et al. 2017; Fig. S19j-p) and immunolocalization of this protein on polytene chromosome squashes (Fig. 12). The DamID data on salivary glands (Posukh et al. 2017) show SUUR binding nearly to the whole fourth polytene chromosome (Fig. S19l), while immunostaining of polytene chromosome squashed preparations detects up to four discrete signals of this protein: near the centromere and telomere regions, and two bands in 102B and 102D regions (Fig. 12). We assume that such DamID results may be due to short-term interaction of SUUR with HP1a covering the fourth chromosome (Pindyurin et al. 2008). However, this issue is yet to be addressed.

*P–*elements (Fig. 13a–c), DNase I hypersensitivity sites (Fig. 14) and ORC2 protein of replication complex binding sites (Fig. 7) in the fourth chromosome are predominantly localized in open aquamarine chromatin of the interbands. These genome elements demonstrate a distribution peak around the 5′UTRs of genes localized in aquamarine chromatin. This data correlate with the whole genome data (Zhimulev et al. 2014). In contrast, the *1360* element, characteristic of the fourth chromosome, tends to occupy band chromatin types: ruby, lazurite, and malachite (Fig. 13d, e).

Thus, the fourth chromosome of *D. melanogaster* differs from the rest of the genome in a large number of gray bands, and the absence of large black intercalary heterochromatin bands. Another difference is the presence of special epigenetic mechanisms of the gene expression regulation by POF, HP1a, and dSETDB1 proteins. At the same time, the results obtained show that in general, the band organization of the fourth chromosome coincides with the organization of the rest of the genome.

## Electronic supplementary material

ESM 1 (DOCX 15 kb)

ESM 2 (DOCX 45 kb)

ESM 3 (PDF 379 kb)

ESM 4 (PDF 296 kb)

ESM 5 (PDF 1010 kb)

ESM 6 (PDF 1050 kb)

ESM 7 (PDF 346 kb)

ESM 8 (PDF 188 kb)

ESM 9 (PDF 399 kb)

ESM 10 (PDF 155 kb)

ESM 11 (PDF 193 kb)

ESM 12 (PDF 179 kb)

ESM 13 (PDF 190 kb)
