# Supplementary material for: Molecular and genetic organization of bands and interbands in the dot chromosome of Drosophila melanogaster
Source: Chromosoma. 2019 Apr 30;128(2):97–117. doi: 10.1007/s00412-019-00703-x (PMC6536484; doi:10.1007/s00412-019-00703-x)
Supplement: Supplementary file 1 — (DOCX 15 kb) [file 412_2019_703_MOESM1_ESM.docx]

**Supplementary text 1**

**Comparison of different chromatin state models relative to the fourth chromosome bands and interbands**

In recent studies, various types of *Drosophila* chromatin have been determined using different input data and computer modeling (Filion et al. 2010; Kharchenko et al. 2011; Milon et al. 2014; Zhimulev et al. 2014; Boldyreva et al. 2017). The five chromatin types are based on the distribution of 53 proteins obtained in DamID experiments (Filion et al. 2010). The modENCODE consortium identified the prevalent combinatorial patterns of 18 histone modifications (CHIP-on-chip data) and determined nine combinatorial chromatin states (Kharchenko et al. 2011). The 3CM model created by Milon et al. (2014) is based on general chromatin sensitivity to DNase I. This model revealed three states: open, closed, and neutral (Milon et al. 2014). However, the correlation of these chromatin types (Filion et al. 2010; Kharchenko et al. 2011; Milon et al. 2014) with bands and interbands of polytene chromosomes remains a problem to be solved. In this work we used our four chromatin state model based on the distribution of interband-specific proteins (Zhimulev et al. 2014; Boldyreva et al. 2017; current investigation) since it showed a good correspondence of distinguished chromatin types to a number of bands and interbands with already determined position on the molecular map. Using this model and FISH, we determined the genomic coordinates of the cytological structures of the fourth chromosome that allowed us to study the molecular characteristics of these structures using whole genome data.

Here we compared these chromatin state models (Filion et al. 2010; Kharchenko et al. 2011; Milon et al. 2014; Zhimulev et al. 2014; Boldyreva et al. 2017) relative to our data on the genomic localization of bands and interbands of the fourth chromosome (Supplementary Fig. S2).

In terms of the four chromatin state model (Zhimulev et al. 2014; Boldyreva et al. 2017; current investigation), the interbands of the fourth chromosome mostly correspond to open aquamarine chromatin. Gray bands predominantly contain lazurite chromatin of intermediate compaction. Almost all compact ruby chromatin is embedded in dense black bands. Both gray and black bands are depleted in open aquamarine chromatin and contain some transitional malachite chromatin (Fig. S2a). Thus, according to the four chromatin states, all the varieties of the fourth chromosome morphological structures differ well from each other.

According to the five chromatin states (Filion et al. 2010), GREEN chromatin is overrepresented in all classes of morphological structures of the fourth chromosome, which would complicate mapping. The interbands are enriched in active YELLOW chromatin (Fig. S2b), but there are only 19 loci of this chromatin type, which doesn`t correspond to the number of interbands (26 items) on the cytological map of the fourth polytene chromosome by C. Bridges (1935). Surprisingly, active RED chromatin is comprised in dense black bands (Fig. S2b).

The fourth chromosome is characterized by high levels of H3K9me2/me3 specific to state 7, dark blue (Kharchenko et al. 2011). State 7 occupies the bulk of both interbands and bands of the fourth chromosome, which would complicate mapping as in the case with five chromatin types (Fig. S2c, d). The interbands are rather different from other structures since they contain a lot of state 1, red, identified by prominent enrichment in H3K4me3/me2 and H3K9ac (Fig. S2c, d). The problem is that gray and black bands have no qualitative differences and they cannot be distinguished from each other while mapping.

The approach of determining the overall chromatin sensitivity to DNaseI revealed the enrichment of the dot chromosome with neutral chromatin (Milon et al. 2014). This chromatin state is represented in all types of structures of the fourth chromosome (Fig. S2e). Over 80% of the total interband length contains open chromatin, but again there are only 19 loci of this chromatin state in conditions of 26 interbands as in case of five chromatin states (Filion et al. 2010), and not all of them are located in the interbands (Fig. S2e). The gray and black bands have no differences at a qualitative level and it is impossible to differentiate them (Fig. S2e).

To sum up, our four chromatin state model (Zhimulev et al. 2014; Boldyreva et al. 2017) is a convenient tool for matching polytene chromosome bands and interbands with the molecular map of the *Drosophila* genome since these structures correspond to specific chromatin types and differ from each other (Fig. S2a). The model shows good correspondence to the cytological structures with genomic coordinates determined earlier (Zhimulev et al. 2014). This is due to the fact that the model is based on the distribution of interband-specific proteins and reveals a special "interband" aquamarine chromatin type (Zhimulev et al. 2014; Zykova et al. 2018). The interbands are the boundaries between physical (topologically associated) domains, and other models based on other input data reveal epigenetic rather than physical domains, and the boundaries of this domain types do not always coincide (Hou et al. 2012; Stadler et al. 2017). Thus, our model of four chromatin types is the most suitable model for solving the problem of superposing the bands and interbands of polytene chromosomes with the physical map of the *Drosophila* genome.
