# Supplementary material for: Molecular and genetic organization of bands and interbands in the dot chromosome of Drosophila melanogaster
Source: Chromosoma. 2019 Apr 30;128(2):97–117. doi: 10.1007/s00412-019-00703-x (PMC6536484; doi:10.1007/s00412-019-00703-x)
Supplement: Supplementary file 2 — (DOCX 45 kb) [file 412_2019_703_MOESM2_ESM.docx]

**Supplementary text 2**

**Functional map of the fourth chromosome of *Drosophila melanogaster***

As a result of overlaying of the fourth polytene chromosome bands and interbands (Fig. S1) on the physical map, a functional map of the chromosome studied was constructed. Figures S8-S16 consistently show its fragments.

In the fourth polytene chromosome of the $\hat{XY}\hat{XY}$/$\hat{XY}$; *y w; SuUR^ES^, Su(var)3-9^06^* fly stock from the centromeric heterochromatin to the *PlexB* gene (Fig. S8c, e) one large 101E1-2 band is visible (Fig. S1a, b). On the molecular map of the *Drosophila* genome, this site begins with a large zone of uncertainty with sparse inclusions of ruby chromatin, followed by alternation of malachite and lazurite chromatin. Although lazurite chromatin basically corresponds to the coding parts of genes (Zhimulev et al. 2014), in the most proximal region of the fourth chromosome sequenced portion the lazurite chromatin blocks lack genes (Fig. S8c, d). The *PlexB* probe marks the distal edge of the 101E1-2 band on the FISH preparation (Fig. S8e-f, Fig. S3a, Fig. 4a). The thin loose gray band 101F1, surrounded by two interbands, is distal to the 101E1-2 band (Fig. S1). The proximal interband in this section, 101E1-2/F1, is represented by aquamarine chromatin, and is of ~ 3.8 kb in size; it contains the 5'UTR of the *PlexB* gene and the interband-specific proteins CHRIZ and WDS (Fig. S8c-e, g). Adjoined to this interband is the 101F1 fine loose gray band, which can only be detected at the electron microscopy level (Fig. S1b). On the molecular map it is represented by the lazurite and malachite chromatin of ~ 10 kb in size (Fig. S8d, e). This structure is occupied by the coding part of the ci gene. The probe from this gene marks the entire space between the 101E1-2 and 102A1-2 bands (Fig. S8c, e, f, Fig. S3b, Fig. 4a). The distal part of the section under consideration, namely the 101F1/102A1-2 interband, is occupied by a large block of open aquamarine chromatin (~ 15 kb) and has several sites of CHRIZ and WDS protein localization (Fig. S8d, e, g). The large size of this interband can be explained by the fact that it contains the 5'UTRs of the ci and pan genes, and it also contains the ubiquitously active (according to FlyBase) gene of the ribosomal protein RpS3A (Fig. S8c, e). The first visible at the level of light microscopy gray 102A1-2 band is located distal to the 101F1/102A1-2 interband (Fig. 1c-e, Fig. S1). The *pan* probe marks this band on the FISH preparation (Fig. S8c, e, f, Fig. S3c, Fig. 4a). On the molecular map it basically consists of the lazurite chromatin and has a size of about 57 kb. It contains the aquamarine chromatin inclusion of 0.8 kb, which corresponds to the *pan* gene alternative promoter. The band is occupied by structural parts of the *pan* and *Ank* genes, which begin in the interbands bordering it (Fig. S8c-e).

The electron microscopy does not detect the 102A3 band between the 102A1-2 and 102A4-5 bands. Thereby, the 102A1-2/A4-5 interband is located between them (Fig. S1). On the molecular map, the interband corresponds to the 8 kb aquamarine chromatin domain containing the CHRIZ and WDS proteins and the 5'UTRs of the *Ank* and *CG32000* genes (Fig. S9c-e, g). The 102A4-5 band (Fig. S1), according to the model of four chromatin types (Boldyreva et al. 2017; Zhimulev et al. 2014), is ~ 41 kb in size and contains compact ruby chromatin and malachite chromatin, and also there are domains of lazurite chromatin at the edges of this band, containing structural parts of ubiquitously active *CG32000* and *Arl4* genes (Fig. S9c-e). Such structures have been described more detailed in the article by Khoroshko et al. (2018). Also this band has a small inclusion of aquamarine chromatin, which is associated with the CHRIZ protein. However, it does not contain 5`UTRs of genes (Fig. S9c-e, g). It has been shown that similar inclusions of aquamarine chromatin in ruby-comprising large compact bands of intercalary heterochromatin contain enhancers and insulators (Khoroshko et al. 2016). The composition of the 102A4-5 band includes five genes, and two of them have 5'UTRs in flanking interbands (Fig. S9c, e). The CG32006 probe marks this band on FISH preparations (Fig. S9c, e, f, Fig. S3d, Fig. 4a). The distal part of the 102A4-5 band on the molecular map is limited by the aquamarine chromatin domain of ~ 2.5 kb, which contains the 5'UTR of the Arl4 gene and the interband-specific proteins CHRIZ and WDS (Fig. S9c-e, g). On the cytological map of the fourth polytene chromosome, this domain corresponds to the 102A4-5/A6 interband (Fig. S1). After the 102A4-5 band at the maximum resolution of electron microscopy, three series of condensed chromosome material can be seen, with the left one corresponding to the thin gray 102A6 band depicted on the chromosome map by C. Bridges. There are hints of two more bands existence; we designated them 102A6' and 102A6'' (Fig. S1b). On the molecular map, these three bands are mainly composed of lazurite chromatin, with small fragments of malachite chromatin, and they are ~ 8.5 kb, ~ 8.7 kb and ~ 14.7 kb in size, respectively. According to our data, they contain structural parts of housekeeping genes (Fig. S9c-e). Each of the three bands described is surrounded by interbands, represented by small-size aquamarine domains on the molecular map(~ 2.5 kb, ~ 4 kb, approximately 6 kb, and ~ 2.3 kb), containing either one 5'UTR of a gene (102A4-5/A6 and 102A6/A6' interbands), or 5'UTRs of two genes located "head-to-head" (interbands 102A6'/A6''and 102A6''/B1-2) (Fig. S9c-e). The first three interbands in the interval described contain CHRIZ and WDS proteins, the latter does not contain this proteins (Fig. S9e, g). Perhaps this is related to the fact that the most distal band in this interval is extremely rarely seen. After the series of the finest gray bands described, there is a large densely packed black 102B1-2 band (Fig. S1) of ~ 77.5 kb in size. It is represented by ruby and malachite chromatin types, characteristic of tightly packed bands, and contains six genes, two of which have 5'UTRs in flanking interbands (Fig. S9c-e). The probe from the *NfI* gene marks this band on the FISH preparations (Fig. S9c, e, f, Fig. S3d-f, Fig. 4a). The distal part of the 102B1-2 band is flanked by ~12.3 kb open chromatin area, represented equally by two fragments of aquamarine chromatin surrounding the lazurite fragment. Aquamarine fragments contain CHRIZ and WDS proteins and 5'UTRs of the *Syt7*, *Rad23*, *Zip102B* and *CG32850* genes, located "head-to-head" pairwise (Fig. S9c-e, g). The size of all three fragments is so small that at the cytological level the entire area is perceived as a single 102B1-2/B3-4 interband (Fig. S1). The size of the lazurite chromatin inclusion in the interband is ~ 4 kb, while the size of the smallest fragment that can form a band detectable at the electron microscopy level is ~ 5 kb (Semeshin *et al*., 1989). The probe from the *Zip102B* gene (lazurite chromatin) marks this interband on FISH preparations (Fig. S9c-f, Fig. S5a, Fig. 4b).

The gray 102B3-4 band (Fig. S1) is mainly represented by lazurite chromatin. It has a small inclusion of malachite chromatin in the region of one of the *PMCA* gene introns. Its total size is ~40 kb. It is completely occupied by the structural parts of the *CG32850* and *PMCA* genes, with 5'UTRs located in flanking interbands (Fig. S10c-e). After the 102B3-4 band, there is the 102B3-4/B5-6 interband (Fig. S1), which is labeled with a probe from the *Hcf* gene on FISH preparations (Fig. S10c, e, f, Fig. S5b, Fig. 4b). On the molecular map, this interband is represented by aquamarine chromatin domain of ~ 4 kb in size, which contains the CHRIZ and WDS proteins, and the 5'UTRs of the *PMCA* and *Hcf* genes located "head-to-head" (Fig. S10c-e, g). The large 102B5-6 band (Fig. S1) is labeled with a probe from the *Lin29* (*dati*) gene on FISH preparations (Fig. S10c, e, f, Fig. S3e, Fig. 4a). This band has a size of ~ 81 kb. It is mainly represented by dense malachite and ruby chromatin. It contains small inclusions of aquamarine chromatin of 0.6 and 1.6 kb that do not contain genes or their parts and clear signals of CHRIZ and WDS proteins (Fig. S10c-e, g). It has been shown that similar inclusions of aquamarine chromatin in ruby-comprising large compact intercalary heterochromatin bands contain enhancers and insulators (Khoroshko et al. 2016). The band contains three genes, two of them start in adjacent interbands (Fig. S10c, e). Lazurite chromatin domains, containing structural parts of ubiquitously active *Hcf* and *Igs* genes, are joined to the edges of the band (Fig. S10c-e). The band size significantly increases because of combining the middle dense area of ruby and malachite chromatin with intermediate lazurite chromatin, which usually corresponds to gray bands. Such bands are described in more detail in the article by Khoroshko et al. (2018).

Between the 102B5-6 band and the next black band there are three interbands and two bands between them, 102B7 and 102B8-9 (Fig. S1). All interbands correspond to the localization of open chromatin proteins, CHRIZ and WDS. The first interband, 102B5-6/B7, is an aquamarine chromatin domain of ~ 6 kb, containing the 5'UTRs of the *Igs* and *CaMKI* genes located "head-to-head". The second and the third interbands have a complex structure. The 102B7/B8-9 interband contains the ~5.2 kb inclusion of lazurite chromatin between two small areas of aquamarine chromatin of ~ 2.5 and 2.6 kb in size. This inclusion corresponds to the coding part of the bip2 gene, with the promoter located in the proximal aquamarine section of this interband. The 102B8-9/С1-2 interband is ~6.1 kb in size, and it contains the ~1.7 kb inclusion of malachite chromatin corresponding to the *Asator* intron. This inclusion is located between two small aquamarine domains giving rise to two alternative *Asator* promoters (Fig. S11c-e, g). The size of the lazurite and malachite chromatin inclusions in these interbands is small to form individual bands (Semeshin et al. 1989). *Asator* FISH probe in the proximal aquamarine domain is located in the 102B8-9/С1-2 interband (Fig. S11c-f, Fig. S5c, Fig. 4b). The 102B7 and 102B8-9 bands are entirely composed of lazurite chromatin and contain the structural parts of the genes that begin in the proximal and distal interbands in this region (Fig. S11c-e). The dense black 102C1-2 band (Fig. S1) on the molecular map is represented by a ~ 68 kb fragment, mainly consisting of ruby and malachite chromatin. The distal border is represented by lazurite chromatin, which contains the structural part of the ubiquitously active *Thd1* gene. The 5’UTR of this gene is located in the distal aquamarine fragment. This lazurite border domain is to correspond to a gray band, but without an interband between them, it attached to ruby-containing 102С1-2 band (Fig. S11c-e), as described by Khoroshko *et al.* (2018). The 102C1-2 band contains an inclusion of aquamarine chromatin without the CHRIZ and WDS proteins, wherein the promoter of the weakly expressed (according to FlyBase) *zfh2* gene is located (Fig. S11c-e, g). It has been shown that similar inclusions of aquamarine chromatin in ruby-containing large compact intercalary heterochromatin bands contain enhancers and insulators (Khoroshko et al. 2016). The *zfh2* probe, taken from the middle of this section, precisely marks the 102C1-2 band on squashed polytene chromosome preparations (Fig. S11c, e, f, Fig. S3f, Fig. 4a). After the 102C1-2 band, a thin gray dashed 102C3 band can be identified using electron microscopy; this band is also drawn on the map by C. Bridges (Fig. S1). This band of ~ 11 kb in size consists mainly of lazurite chromatin and contains the coding parts of the *Pur-alpha* and *ND-49* genes, with their 5'UTRs located in flanking interbands. Small malachite inclusions correspond to the introns of the *Pur-alpha* gene (Fig. S11c-e). Aquamarine chromatin domains with CHRIZ and WDS proteins correspond to the interbands surrounding this thin gray band. The 102С1-2/С3 interband of ~3,6 kb in size contains bidirectional promoters of the *Thd1* and *Pur-alpha* genes. The 102С3/С4-5 interband of ~3 kb in size contains bidirectional promoters of the *ND-49* and *Ephrin* genes (Fig. S11c-e).

The **102С4-5** band (Fig. S1) of intermediate size (~37 kb) contains malachite chromatin with ruby chromatin amidst, and also it contains lazurite chromatin adjacent to the proximal part of the band, which corresponds to the second type of band borders, described in the work by Khoroshko and coworkers (Khoroshko et al. 2018). Three genes are located in the band. The 5`UTR of the first of them (ubiquitously active *Ephrin* gene) is situated in the proximal interband, and the coding part of this gene is in lazurite chromatin (Fig. S12c-e). The 102С4-5 band is clearly marked with the *CG1909* FISH probe from ruby chromatin (Fig. S12c-f, Fig. S3g, Fig. 4a). The *Eph* FISH probe paints the distal 102С4-5/С6-7 interband (Fig. S12c, e, f, Fig. S5d, Fig. 4b) of ~2,6 kb in size, consisting of aquamarine chromatin and containing the 5` end of the *Eph* gene, and CHRIZ and WDS proteins (Fig. S12c-e, g). The **102C6-7** band (Fig. S1) has a complex structure in the context of four chromatin states. It includes all four types of chromatin, and has a size of ~ 45.5 kb. Tightly packed ruby chromatin is in the middle of the band, small fragments of malachite adhere to both sides of it, and then through the fragments of aquamarine chromatin not being the interbands, lazurite chromatin is attached to both ends of this domain (Fig. S12d, e). It has been shown that similar inclusions of aquamarine chromatin in ruby-containing large compact intercalary heterochromatin bands contain enhancers and insulators (Khoroshko et al. 2016). The *mav* and *Ekar* FISH probes from inner aquamarine inclusions containing weak signals of CHRIZ and WDS proteins are localized in the 102С6-7 band on polytene chromosome squashes (Fig. S12c-g, Fig. S5e, f, Fig. 4b). This band contains five genes, and 5`UTRs of two of them are located in the flanking interbands (Fig. S12c, e). Such features of the four chromatin states map of this band might be caused by the genome plasticity and the differences in gene expression in diploid cell cultures used to construct the model, and the expression in salivary glands comprising polytene chromosomes. On the cytological map of polytene chromosomes the 102С6-7/С8 interband is a broad light structure (Fig. S1). On the molecular map it corresponds to a fragment of ~13 kb and it has a complex structure. This interband contains a ~ 4.7 kb inclusion of lazurite chromatin, which apparently does not form a separate band (Semeshin et al. 1989). This inclusion is surrounded by two aquamarine fragments containing 5’ ends of genes (*gw* gene in the proximal aquamarine, and *Slip1* and *CG11360* genes located “head-to-head” in the distal aquamarine), and CHRIZ and WDS proteins (Fig. S12c-e, g). The *Slip1* probe from the lazurite inclusion accurately marks this interband (Fig. S12c-f, Fig. S5g, Fig. 4b). Proximal to this interband there is a thin gray 102C8 band clearly visible at the level of light and electron microscopy (Fig. 1c-e, Fig. S1). On the physical map this band coincides with coding parts of the *CG11360* and *myo* genes, coming from the flanking interbands. Basically, this band is represented by lazurite chromatin; a small inclusion of malachite chromatin corresponds to the intergenic spacer, and the micro inclusions of aquamarine contain neither the 5` ends of genes nor CHRIZ and WDS proteins (Fig. S12c-e, g). It has been shown earlier that similar aquamarine inclusions in large ruby intercalary heterochromatin bands contain enhancers and insulators (Khoroshko et al. 2016). Also this band contains large sites where the chromatin state is not determined. These sites correspond to the *CG11360* introns (Fig. S12c-e).

After the 102С8 band there are the 102С8/D1-2 interband and **102D1-2**, the biggest black band of the fourth chromosome (Fig. S1). On the molecular map, the 102С8/D1-2 interband is a ~4 kb aquamarine domain containing *myo* promoter directed towards the proximal band, and CHRIZ and WDS proteins (Fig. S13c-e, g). The probe near the *myo* gene promoter marks this interband on the FISH preparations (Fig. S13c, e, f, Fig. S5g, h, Fig. 4b). The 102D1-2 band of ~89 kb in size is mainly represented by ruby chromatin; small malachite inclusions correspond to the intergenic spacer, and the aquamarine inclusion does not contain parts of genes and has only a very weak signal of CHRIZ binding. It has been shown that similar inclusions of aquamarine chromatin in ruby-containing large compact intercalary heterochromatin bands contain enhancers and insulators (Khoroshko et al. 2016). The 102D1-2 band contains structural parts of *ey* and *MED26* genes and a long *bt* gene between them (Fig. S13c-e, g). The *bt* FISH probe paints this band on polytene chromosome squashes (Fig. S13c, e, f, Fig. S4b, Fig. 4a). The distal boundary of the 102D1-2 band is limited by lazurite chromatin domain, which corresponds to connection of a gray band to a large black band (Khoroshko et al. 2018). The 102D1-2/D4-5 interband is represented by ~2 kb of aquamarine chromatin and contains the 5’UTR of the *MED26* gene, and CHRIZ and WDS proteins (Fig. S13c-e, g). Since the loose discontinuous 102D3 band (Fig. S1a), shown on the map by C. Bridges, is not visualized on chromosome preparations, the **102D4-5** band is located distal to the 102D1-2 band. These bands are separated by the 102D1-2/D4-5 interband described above (Fig. S1). The large dense black 102D4-5 band on the molecular map of the *Drosophila* genome has a size of ~76 kb and is mainly represented by ruby chromatin surrounded by small fragments of malachite chromatin. Small fragments of malachite chromatin are also located inside ruby chromatin (Fig. S13d, e). The Sox102F probe (ruby chromatin) clearly marks this band (Fig. S13c-f, Fig. S4b, Fig. 4a). This band has two small aquamarine chromatin inclusions of ~ 1.4 kb and ~ 2.1 kb in size. The first of them corresponds to the intron of the *Sox102F* gene and has CHRIZ binding sites only in a few cell cultures. The second one contains a clear signal of CHRIZ and WDS binding in different cell cultures and the *Sox102F* 5' end (Fig. S13c-e, g). However, according to FlyBase, this gene encodes a transcription factor, mainly responsible for the development and functioning of heart and wing veins, and it is not expressed in larval salivary glands. Due to the lability of the genome such aquamarine fragment does not form an interband in salivary gland polytene chromosomes. The probe from this aquamarine fragment, designated as *1_Sox102F*, also marks the 102D4-5 band on FISH preparations (Fig. S13d-f, Fig. S6a, Fig. 4b). It has been shown that similar inclusions of aquamarine chromatin in ruby-containing large compact intercalary heterochromatin bands comprise enhancers and insulators (Khoroshko et al. 2016). The distal edge of the 102D4-5 band on the molecular map is represented by lazurite chromatin and it contains the structural part of the ubiquitously active *CG11148* (*Gyf*) gene, having its 5' end in the distal interband (Fig. S13c-e). This corresponds to the second type band edge formed when joining the gray band to a large black band (Khoroshko et al. 2018). In total, the band contains four genes: *CG11231*, *Sox102F*, *fd102C* and *CG11148*. The distal edge of the 102D4-5 band is restricted to the 102D4-5/D6-7 interband (Fig. S1). According to the model of four chromatin types, this interband occupies ~ 15 kb and has a complex structure. The 102D4-5/D6-7 interband is represented by two aquamarine chromatin domains surrounding a ~7 kb fragment of lazurite chromatin that does not form a separate band, though in some cases a band has been formed by a structure of ~ 5 kb in size (Semeshin et al. 1989). Aquamarine domains contain CHRIZ and WDS proteins. The proximal aquamarine fragment contains the 5'UTR of an ubiquitously active *CG11148* gene, the structural part of which is located in the 102D4-5 band. The distal aquamarine fragment does not contain 5' ends of protein-coding genes, but it contains the CR44029 transcription start site (Fig. S13c-e, g). Probes from both aquamarine domains, namely the *CG11148* probe (Fig. S13d-f, Fig. S6b, Fig. 4b), and the *unc-13* probe (Fig. S13d-f, Fig. S6c, Fig. 4b), mark the 102D4-5/D6-7 interband on FISH preparations.

A rather large band of the fourth chromosome, **102D6-7** (Fig. S1), is represented mainly by lazurite chromatin and has a size of ~ 49 kb. It contains parts of two genes: *unc-13* and *eIF4G*, with the ends of several isoforms of the unc-13 gene located in the internal inclusions of aquamarine chromatin. The first aquamarine chromatin fragment contains CHRIZ and WDS binding sites (Fig. S14c-e, g). However, according to FlyBase, the *unc-13* gene is not expressed in salivary glands, and, due to the genome lability, this aquamarine fragment does not form an interband in salivary gland polytene chromosomes. The *1_unc-13* FISH probe from the aquamarine fragment being considered marks the 102D6-7 band on polytene chromosome squashed preparations (Fig. S14d-f, Fig. S6d, Fig. 4b). The distal edge of this band is restricted to the 102D6-7/D8 interband (Fig. S1), which is represented by a small ~1.7 kb aquamarine fragment and contains the 5'end of a highly active *eIF4G* gene with ubiquitous expression that encodes the translation initiation factor (according to the FlyBase data). The *eIF4G* probe marks this interband on the FISH preparations (Fig. S14c-f, Fig. S6e, Fig. 4b). The coding part of this gene is located in lazurite chromatin of the 102D6-7 band described above (Fig. S14). After the large 102D6-7 band, a thin gray **102D8** band is localized on the cytological map by C. Bridges. This band can be found on some preparations of polytene chromosomes (Fig. 1c-e, Fig. S1). On the molecular map, this thin band is represented mainly by malachite chromatin. It has a size of ~ 13 kb and is fully occupied by the *mGluR* gene active at a low level, mainly in the nervous system (according to FlyBase). Interestingly, this band has a microscopic inclusion of ruby chromatin (Fig. S14c-e). The 102D8/E1-2 interband is distal to the 102D8 band (Fig. S1), and it has a complex structure in the context of four chromatin types. According to the model of four chromatin types, the total size of the interband is ~17 kb, and aquamarine chromatin on both sides is surrounding a lazurite chromatin fragment of ~ 6.5 kb in size (Fig. S14d, e). This inclusion of lazurite chromatin could form a thin gray band, as has been shown, for example, for the 10th region of the X chromosome or when fragments of five kb in length were inserted into the chromosome (Semeshin et al. 1989; Zhimulev et al. 2014). However, the small size of this fragment containing an active housekeeping *4E-T* gene, associated with translation regulation and mRNA stability (according to FlyBase data), leads to the fact that the whole area on polytene chromosome preparations looks as one continuous interband. Two probes from the aquamarine fragments of this interval, *mGluR* and *4E-T*, label the 102D8/E1-2 interband on FISH preparations (Fig. S14d-f, Fig. S6f, g, Fig. 4b). After a thin gray 102D8 band, on the map by C. Bridges and polytene chromosome preparations there is a notable black **102E1-2** band (Fig. S1). According to the four chromatin state model, this band has a size of ~ 59 kb and is represented mainly by dense ruby and malachite chromatin. The band contains three genes: a low-active *fuss* gene, *toy* gene, involved in the development of the eye and nervous system, and the ubiquitously active *PlexA* gene (according to FlyBase). The coding part of the *PlexA* gene is situated in lazurite chromatin (Fig. S14c-e), corresponding to the joining of a gray band to a dense black band, or to the second type band boundary, as described in Khoroshko et al. (2018). In addition, the 102E1-2 band contains several micro inclusions of aquamarine chromatin, which do not contain genes and CHRIZ and WDS proteins (Fig. S14c-e, g). It has been shown that inclusions of aquamarine chromatin in ruby chromatin-containing large black intercalary heterochromatin bands contain enhancers and insulators (Khoroshko et al. 2016). The FISH probe from the *toy* gene clearly marks the 102E1-2 band on polytene chromosome squashes (Fig. S14c, e, f, Fig. S4c, Fig. 4a). The distal boundary of the 102E1-2 band neighbors with the 102E1-2/E3 interband (Fig. S1), represented by aquamarine chromatin domain of ~ 8.6 kb. This aquamarine domain contains CHRIZ and WDS proteins and 5'ends of ubiquitously active (according to the FlyBase) genes *PlexA* and *ATPsynbeta*, and two ubiquitously active (according to the FlyBase) genes, *CG11077* and *CG11076*, entirely (Fig. S14c-e, g). The *CG11076* probe marks the 102E1-2/E3 interband on FISH preparations (Fig. S14e, f, Fig. S6h, Fig. 4b).

After the 102E1-2 band, the map by C. Bridges indicates a thin dotted **102E3** band (Fig. S1a), which is visible in electron microscopy (Fig. S1b) and, in some cases, in light microscopy (Fig. 1c-e). According to the four chromatin states, this band of ~ 17 kb size corresponds to lazurite chromatin and structural parts of a highly expressed *ATPsynbeta* gene and a moderately expressed *CaMKII* gene with ubiquitous activity (according to FlyBase data). The 5`UTRs of these genes are located in the flanking interbands (Fig. S15c-e). The distal part of this band is restricted to the 102E3/E4-5 interband, which is ~ 13.2 kb in size and has a complex structure in the context of four chromatin states. The bulk of the interband is represented by aquamarine chromatin, containing the binding signals of the interband-specific CHRIZ protein and the 5` ends of three housekeeping genes (according to FlyBase): *CaMKII*, *Zyx*, and *Rfabg* (*apolpp*). The middle of the interband contains inclusion of malachite chromatin of ~ 1.8 kb, and lazurite chromatin inclusion of ~ 28 kb (Fig. S15c-e, g), which are not visible as independent structures of chromosomes, because their size is beyond the electron microscopy resolution (Semeshin et al. 1989). Two FISH probes, *CaMKII* (Fig. S15e, f, Fig. S7a, Fig. 4b), and *Zyx* (Fig. S15e, f, Fig. S7b, Fig. 4b), mark this interband on polytene chromosome preparations. A large black **102E4-5** band is distal to the 102E3/E4-5 interband (Fig. S1). On the molecular map it is represented mainly by dense ruby chromatin with small domains of malachite chromatin inside ruby chromatin and on its edges. In the middle of the band there is an aquamarine chromatin micro inclusion of ~ 0.5 kb in size, containing the 5 'end of the *Actbeta* gene active in the neural tissue of larva and fly (according to FlyBase). This aquamarine inclusion apparently does not form an interband in salivary gland polytene chromosomes. In total, the 102E4-5 band contains three genes: *Rfabg* (*apolpp*), *Actbeta*, and *sv*, with the 5`UTRs of *Rfabg* and *sv* lying in the flanking interbands (Fig. S15c-e). The *Rfabg* probe from dense ruby chromatin clearly marks this band on FISH preparations (Fig. S15d-f, Fig. S4d, Fig. 4a). The distal edge of this band is neighboring with the 102E4-5/F1-2 interband (Fig. S1), which on the molecular map has a size of ~ 12.7 kb and is represented by two fragments of aquamarine chromatin surrounding ~7.3 kb lazurite fragment. Aquamarine fragments encompass CHRIZ and WDS localization sites. The proximal aquamarine fragment contains the 3'end of the *sv* gene, the distal aquamarine fragment contains the 5' end of the *Cals* gene and the entire *Arf102F* gene (Fig. S15c-e, g). The coding part of the *Cals* gene is located in the central lazurite fragment, which could have formed a separate thin gray band, but its small size (Semeshin et al. 1989) and the location of a housekeeping *Cals* gene (according to FlyBase) leads to the fact that the entire region is perceived as a continuous light interband. The *Cals* probe from the central lazurite fragment marks this interband on the FISH preparations (Fig. S15d-f, Fig. S7c, Fig. 4b). After the 102E4-5 band there is the **102F1-2** band clearly visible on the map by C. Bridges and squashed polytene chromosome preparations (Fig. S1). On the molecular map, it has a size of ~ 57 kb and contains four genes: *CG11155*, *CG32017*, *Kif3C*, and *pho*, and the latter's end is situated in the distal interband. The 102F1-2 band is mainly represented by malachite chromatin; the center of this band contains dense ruby chromatin, and both edges contain lazurite fragments. In addition, this band contains two inclusions of aquamarine chromatin of ~ 2.3 kb and ~ 1 kb in size. The former contains CHRIZ and WDS binding sites and 5'ends of *CG32017* and *Kif3C* genes, and, according to FlyBase, the first of them is expressed mainly in the nervous tissue of the larvae and flies, and the second one has the same pattern of expression, but at very low level, therefore, probably, this aquamarine fragment does not form an interband in polytene chromosomes of salivary glands. The latter has a small size, a very weak signal of CHRIZ binding, it does not contain 5' ends of genes, corresponds to an intron of the *Kif3C* gene and does not form an interband in salivary gland polytene chromosomes (Fig. S15c-e, g). It has been shown that similar inclusions of aquamarine chromatin in ruby-containing large compact intercalary heterochromatin bands contain enhancers and insulators (Khoroshko et al. 2016). The *CG11155* (Fig. S15e, f, Fig. S4e, Fig. 4a) and *CG32017* (Fig. S15e, f, Fig. S7d, Fig. 4b) FISH probes paint the 102F1-2 band on polytene chromosome squashes. The distal edge of the 102F1-2 band is restricted to the 102F1-2/F3 interband, which, according to the four chromatin states, corresponds to aquamarine chromatin domain of ~ 2.6 kb, containing bright signals of the interband-specific CHRIZ and WDS proteins binding, and the 5` end of an ubiquitously active (according to FlyBase) *pho* gene, the probe from which marks the interband on the FISH preparations (Fig. S15c-g, Fig. S7e, Fig. 4b).

After a clearly visible 102F1-2 band on the chromosome map by C. Bridges (Fig. S1a), at the level of electron microscopy (Fig. S1b) and, in some cases, at the level of light microscopy (Fig. 1c-e) you can see a thin gray **102F3** band. On the molecular map of the genome, this band probably corresponds to a ~30 kb domain containing the *CG33521*, *PIP4K*, *Mitf*, and *Dyrk3* genes, with the last gene's 5'UTR located in the distal interband. According to the model of four chromatin types, the 102F3 band corresponds to malachite and lazurite chromatin; in the middle, it contains an aquamarine chromatin fragment of ~ 4 kb in size with CHRIZ and WDS localization sites and 5' ends of *PIP4K* gene with ubiquitous activity and *Mitf* gene (Fig. S16c-e, g), which is expressed mainly in the ovary (according to FlyBase data). This fragment of aquamarine chromatin could have formed an interband and divide the 102F3 band, but there is only one band on the cytological map by C. Bridges and on squashed polytene chromosome preparations in this interval. The *CG33521* (Fig. S16e, f, Fig. S4f, Fig. 4a) and *PIP4K* (Fig. S16e, f, Fig. S7f, Fig. 4b) FISH probes paint the interval between 102F1-2 and 102F4,5 bands on polytene chromosome squashes. The distal edge of this band is neighboring with the 102F3/F4,5 interband, which, according to the four chromatin states, is an aquamarine chromatin domain of ~ 5.6 kb in size. This aquamarine domain contains bright signals of the interband-specific CHRIZ and WDS proteins and the 5' ends of an ubiquitously active *Dyrk3* gene and active mainly in the nervous tissue *Cadps* gene (according to FlyBase), located "head-to-head" (Fig. S16c-e, g). The *1_Cadps* FISH probe from the aquamarine fragment under consideration marks the 102F3/F4,5 interband (Fig. S16d-f, Fig. S7g, Fig. 4b). The last clearly visible **102F4,5** band, which corresponds to 102F4 and 102F5 bands on the map by C. Bridges (Fig. S1), is composed of ruby and malachite chromatin. If we consider the end of normally defined chromatin types as the distal boundary of the band, then its size is ~35.7 kb, and the entire length of this band is occupied by the coding part of the *Cadps* gene (Fig. S16c-e). The *Cadps* FISH probe marks the 102F4,5 band on polytene chromosome squashes (Fig. S16e, f, Fig. S4g, Fig. 4a). After the band described above, two more subtle bands can be seen on the map by C. Bridges (Fig. S1), which were not found on chromosome preparations of the fly strain used in our study. However, in this fly stock carrying mutations in *SuUR* and *Su(var)3-9* genes, the distal end of the fourth chromosome has a special form. It has the appearance of a long dark gray disordered structure. After the last annotated gene, the DNA sequence is known for extra 76,259 kb. The chromatin types are almost not determined there (Fig. S16e).
