# Supplementary material for: Molecular and genetic organization of bands and interbands in the dot chromosome of Drosophila melanogaster
Source: Chromosoma. 2019 Apr 30;128(2):97–117. doi: 10.1007/s00412-019-00703-x (PMC6536484; doi:10.1007/s00412-019-00703-x)
Supplement: Supplementary file 4 — (PDF 296 kb) [file 412_2019_703_MOESM4_ESM.pdf]

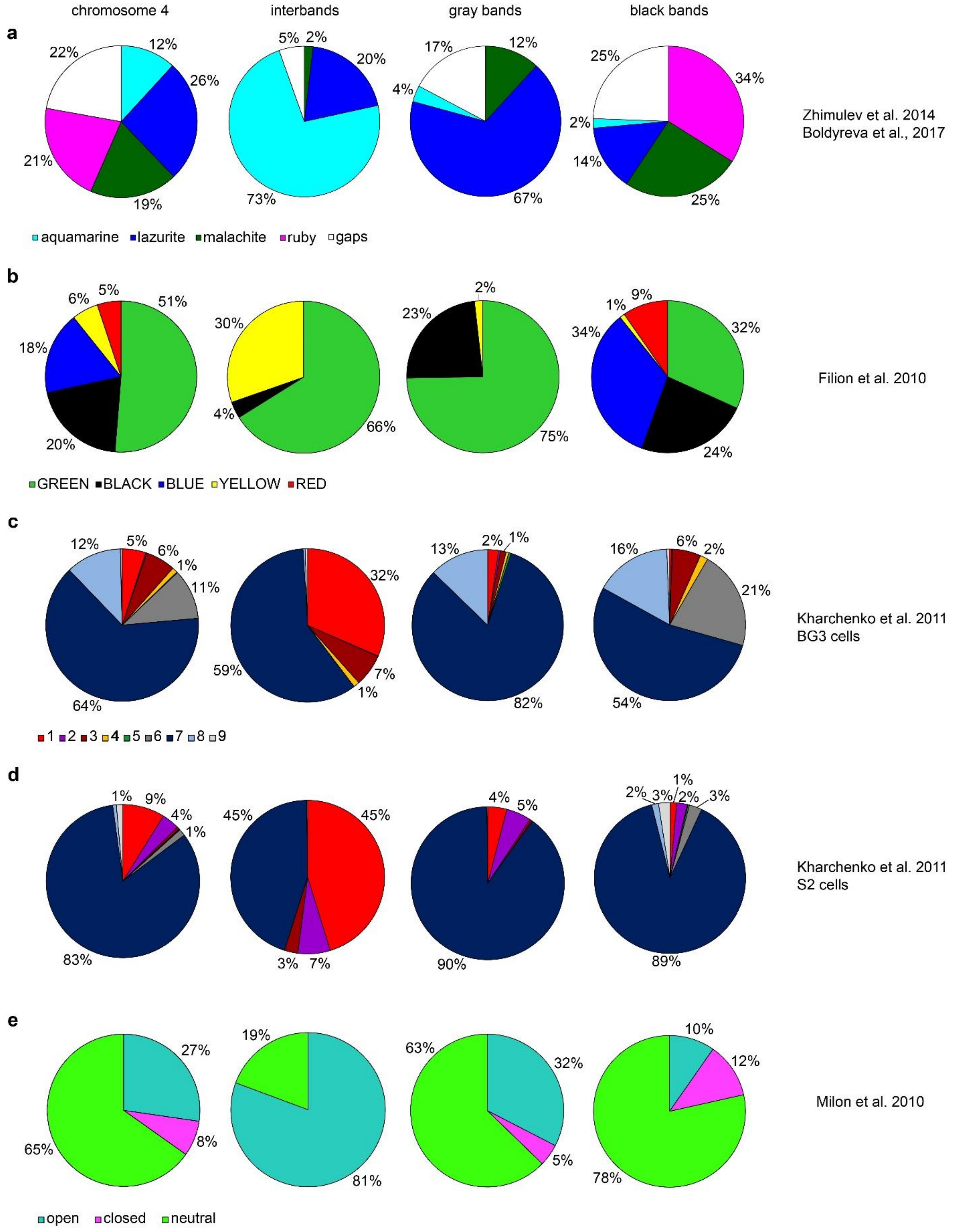

Fig. S2 Different chromatin state models in the composition of the fourth chromosome cytological structures with genomic coordinates determined in current investigation. The diagrams in all lines are arranged in the following order: the composition of the fourth chromosome, its interbands, gray bands, and black bands. The color code is itemized below each line. **a** Four chromatin state model (Zhimulev et al. 2014; Boldyreva et al. 2017). **b** Five chromatin state model (Filion et al. 2010). **c** Nine chromatin states in BG3 cells (Kharchenko et al. 2011). **d** Nine chromatin states in S2 cells (Kharchenko et al. 2011). The color code is the same as in Fig. S2c. **e** Three chromatin states (Milon et al. 2014). Explanations are provided in the Supplementary text 1.
