# Supplementary material for: Molecular and genetic organization of bands and interbands in the dot chromosome of Drosophila melanogaster
Source: Chromosoma. 2019 Apr 30;128(2):97–117. doi: 10.1007/s00412-019-00703-x (PMC6536484; doi:10.1007/s00412-019-00703-x)
Supplement: Supplementary file 5 — (PDF 1010 kb) [file 412_2019_703_MOESM5_ESM.pdf]

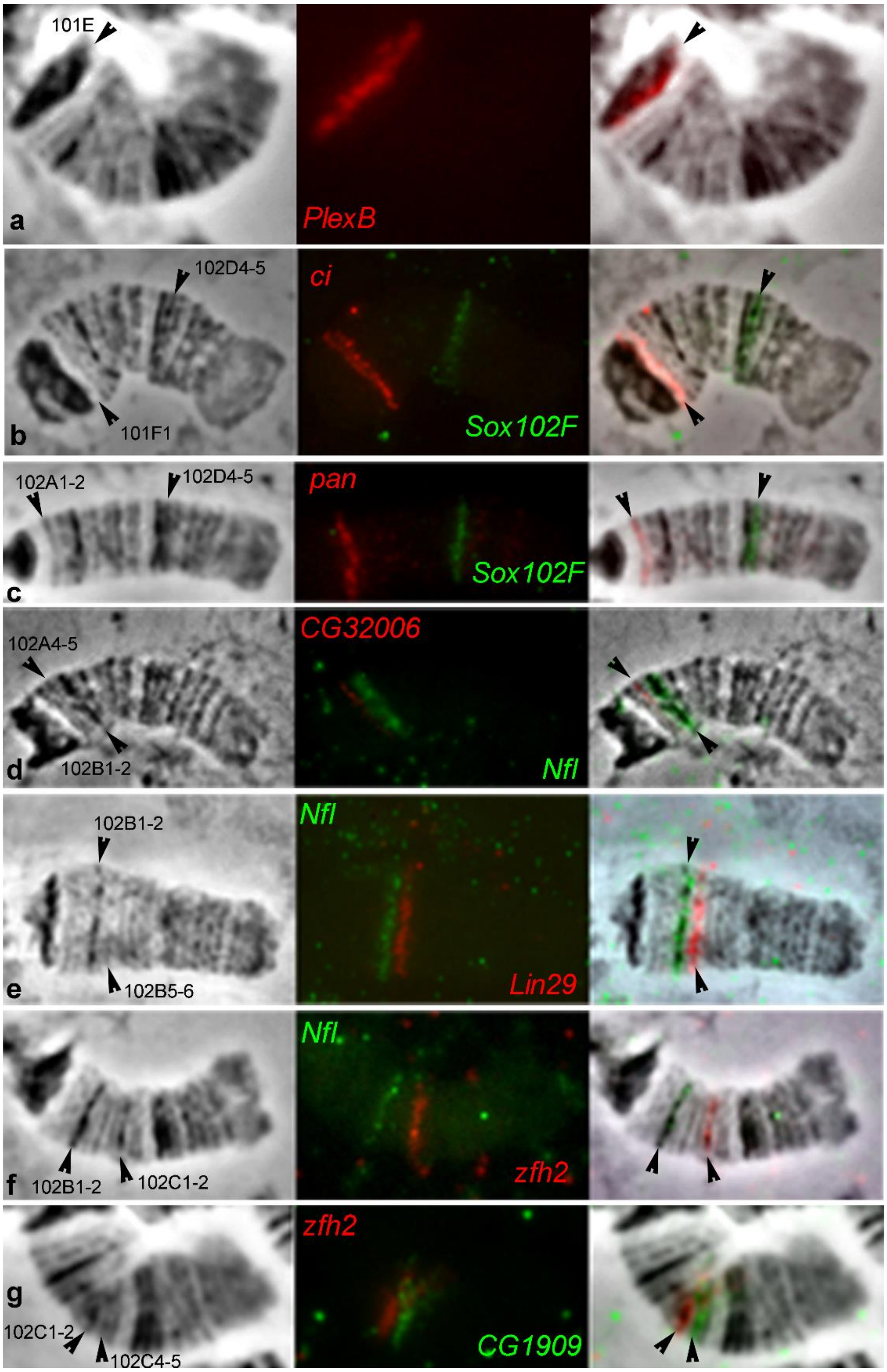

Fig. S3 FISH on the polytene fourth chromosome. Part 1. The probes from the bands predicted by the four chromatin state model (Zhimulev et al. 2014). From left to right: the phase-contrast microphotograph of the fourth chromosome, combined FISH signals, and their superposition. The red signals correspond to probes labeled with TAMRA fluorochrome, the green ones correspond to probes labeled with Fluorescein. The arrowheads indicate: **a** *PlexB* probe in the 101E band; **b** *ci* probe in the 101F1 band, *Sox102F* probe in the 102D4-5 band; **c** *pan* probe in the 102A1-2 band, *Sox102F* probe in the 102D4-5 band; **d** *CG32006* probe in the 102A4-5 band, *NfI* probe in the 102B1-2 band; **e** *NfI* probe in the 102B1-2 band, *Lin29 (Dati)* probe in the 102B5-6 band; **f** *NfI* probe in the 102B1-2 band, *zfh2* probe in the 102C1-2 band; **g** *zfh2* probe in the 102C1-2 band, *CG1909* probe in the 102C4-5 band.

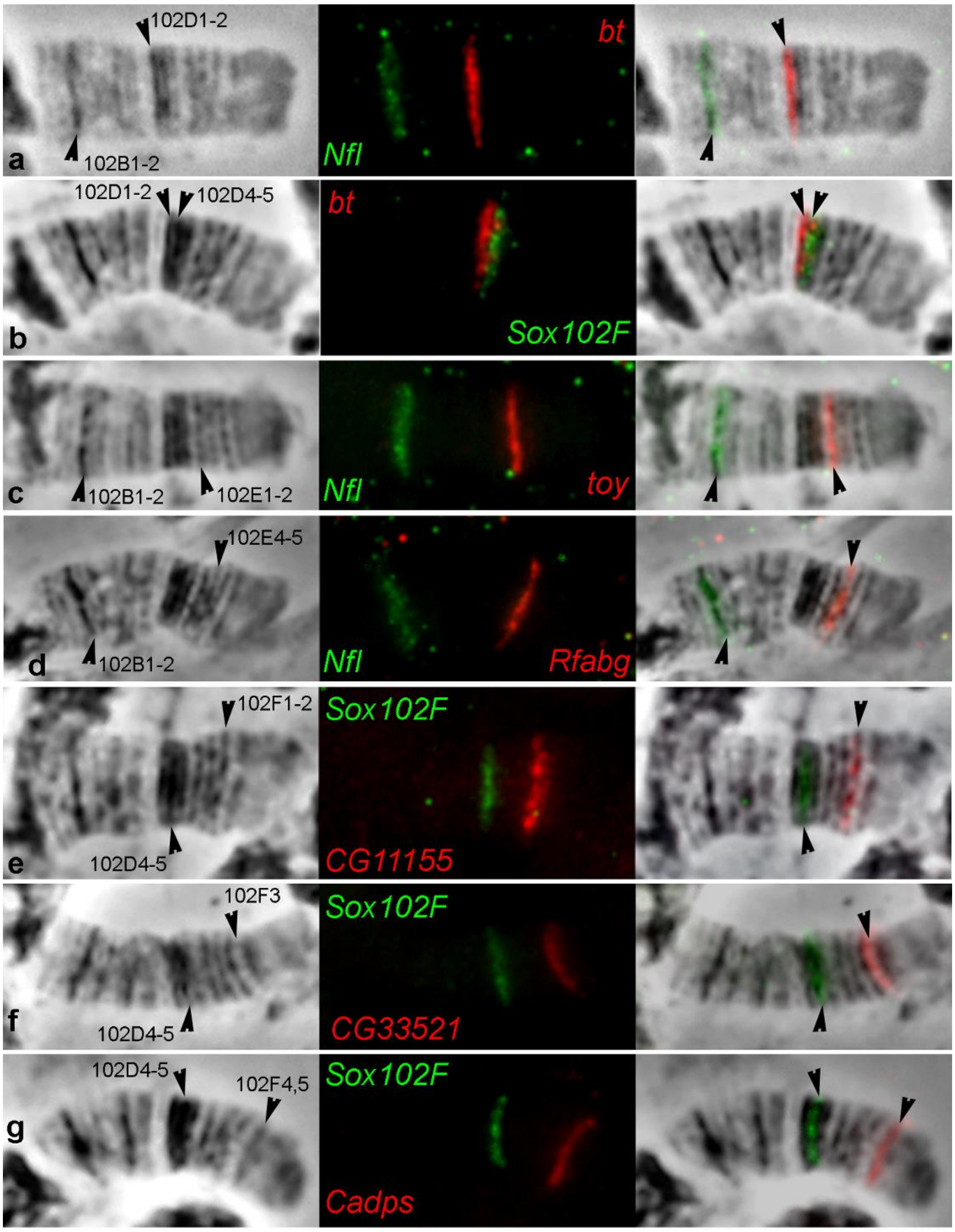

Fig. S4 FISH on the polytene fourth chromosome. Part 2. The probes from the bands predicted by the four chromatin state model (Zhimulev et al. 2014). From left to right: the phase-contrast microphotograph of the fourth chromosome, combined FISH signals, and their superposition. The red signals correspond to probes labeled with TAMRA fluorochrome, the green ones correspond to probes labeled with Fluorescein. The arrowheads indicate: **a** *NfI* probe in the 102B1-2 band, *bt* probe in the 102D1-2 band; **b** *bt* probe in the 102D1-2 band, *Sox102F* probe in the 102D4-5 band; **c** *NfI* probe in the 102B1-2 band, *toy* probe in the 102E1-2 band; **d** *NfI* probe in the 102B1-2 band, *Rfabg* probe in the 102E4-5 band; **e** *Sox102F* probe in the 102D4-5 band, *CG11155* probe in the 102F1-2 band; **f** *Sox102F* probe in the 102D4-5 band, *CG33521* probe in the 102F3 band; **g** *Sox102F* probe in the 102D4-5 band, *Cadps* probe in the 102F4,5 band.

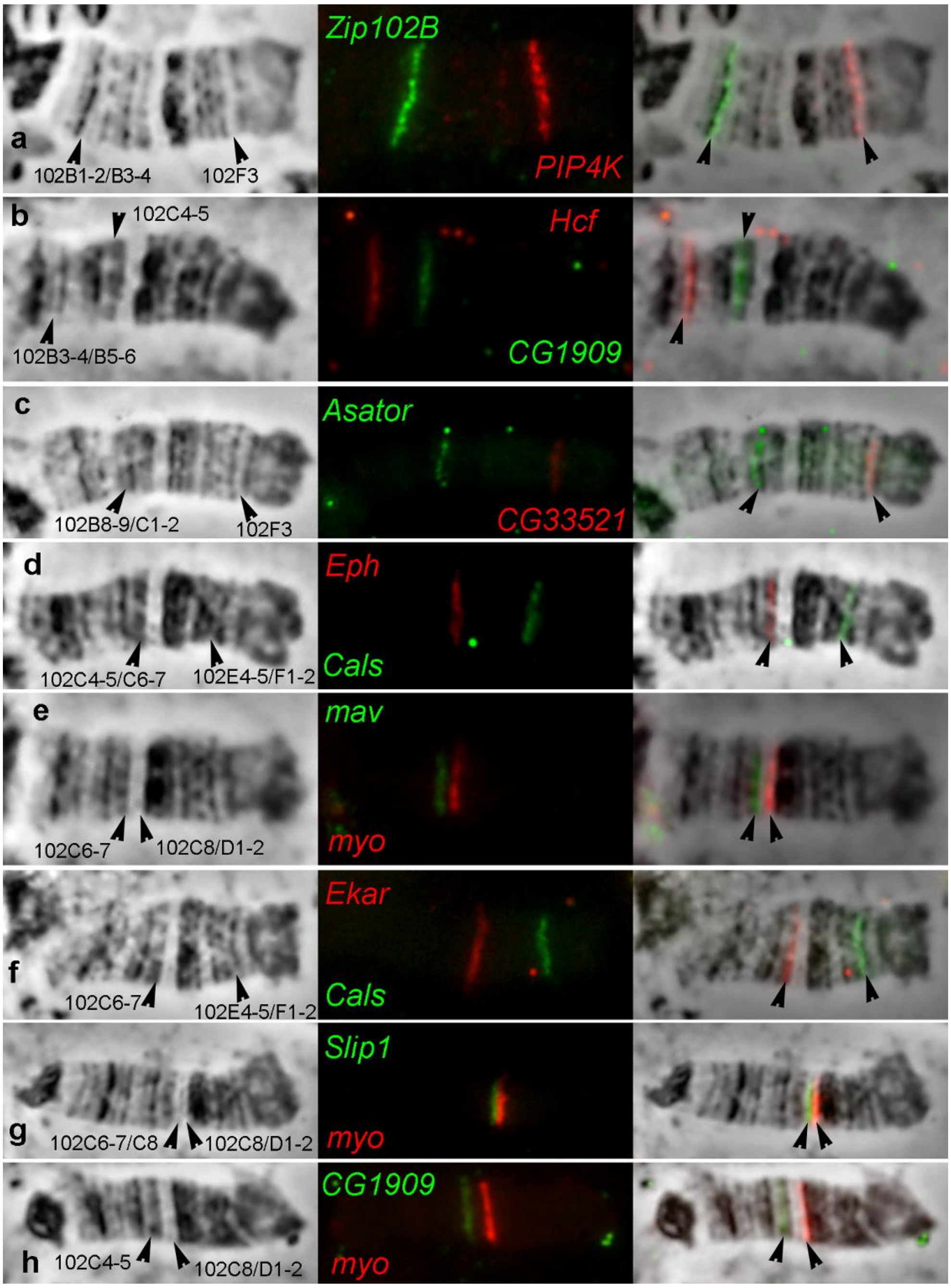

Fig. S5 FISH on the polytene fourth chromosome. Part 3. The probes from the interbands predicted by the four chromatin state model (Zhimulev et al. 2014). From left to right: the phase-contrast microphotograph of the fourth chromosome, combined FISH signals, and their superposition. The red signals correspond to probes labeled with TAMRA fluorochrome, the green ones correspond to probes labeled with Fluorescein. The arrowheads indicate: **a** *Zip102B* probe in the 102B1-2/B3-4 interband, *PIP4K* probe in the 102F3 band; **b** *Hcf* probe in the 102B3-4/B5-6 interband, *CG1909* probe in the 102C4-5 band; **c** *Asator* probe in the 102B8-9/C1-2 interband, *CG33521* probe in the 102F3 band; **d** *Eph* probe in the 102C4-5/C6-7 interband, *Cals* probe in the 102EE4-5/F1-2 interband; **e** *mav* probe in the 102C6-7 band, *myo* probe in the 102C8/D1-2 interband; **f** *Ekar* probe in the 102C6-7 band, *Cals* probe in the 102E4-5/F1-2 interband; **g** *Slip1* probe in the 102C6-7/C8 interband, *myo* probe in the 102C8/D1-2 interband; **h** *CG1909* probe in the 102C4-5 band, *myo* probe in the 102C8/D1-2 interband.

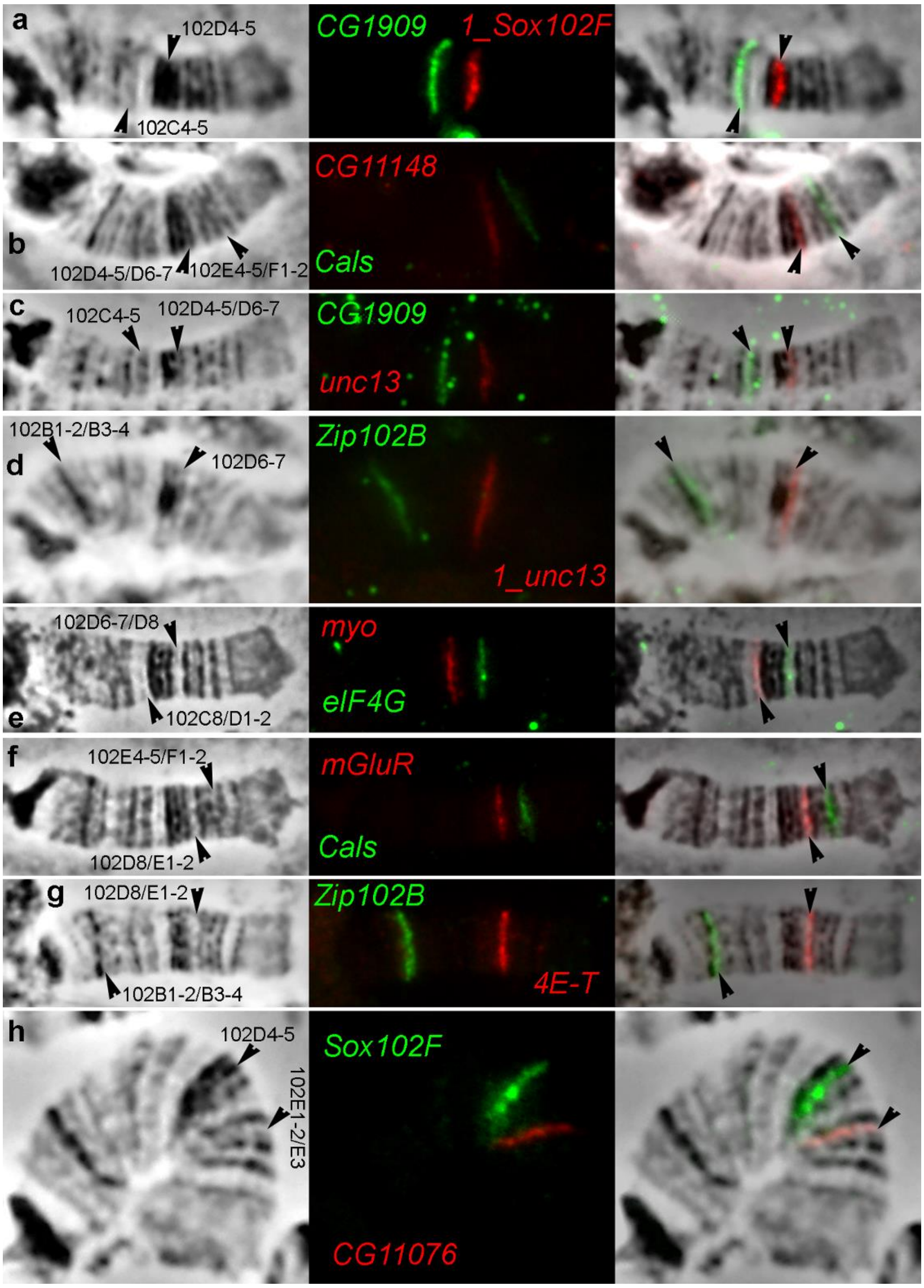

Fig. S6 FISH on the polytene fourth chromosome. Part 4. The probes from the interbands predicted by the four chromatin state model (Zhimulev et al. 2014). From left to right: the phase-contrast microphotograph of the fourth chromosome, combined FISH signals, and their superposition. The red signals correspond to probes labeled with TAMRA fluorochrome, the green ones correspond to probes labeled with Fluorescein. The arrowheads indicate: **a** *CG1909* probe in the 102C4-5 band, *1_Sox102F* probe in the 102D4-5 band; **b** *CG11148* probe in the 102D4-5/D6-7 interband, *Cals* probe in the 102E4-5/F1-2 interband; **c** *CG1909* probe in the 102C4-5 band, *unc13* probe in the 102D4-5/D6-7 interband; **d** *Zip102B* probe in the 102B1-2/B3-4 interband, *1_unc13* probe in the 102D6-7 band; **e** *myo* probe in the 102C8/D1-2 interband, *eIF4G* probe in the 102D6-7/D8 interband; **f** *mGluR* probe in the 102D8/E1-2 interband, *Cals* probe in the 102E3/E4-5 interband; **g** *Zip102B* probe in the 102B1-2/B3-4 interband, *4E-T* probe in the 102D8/E1-2 interband; **h** *Sox102F* probe in the 102D4-5 band, CG11076 probe in the 102E1-2/E3 interband.

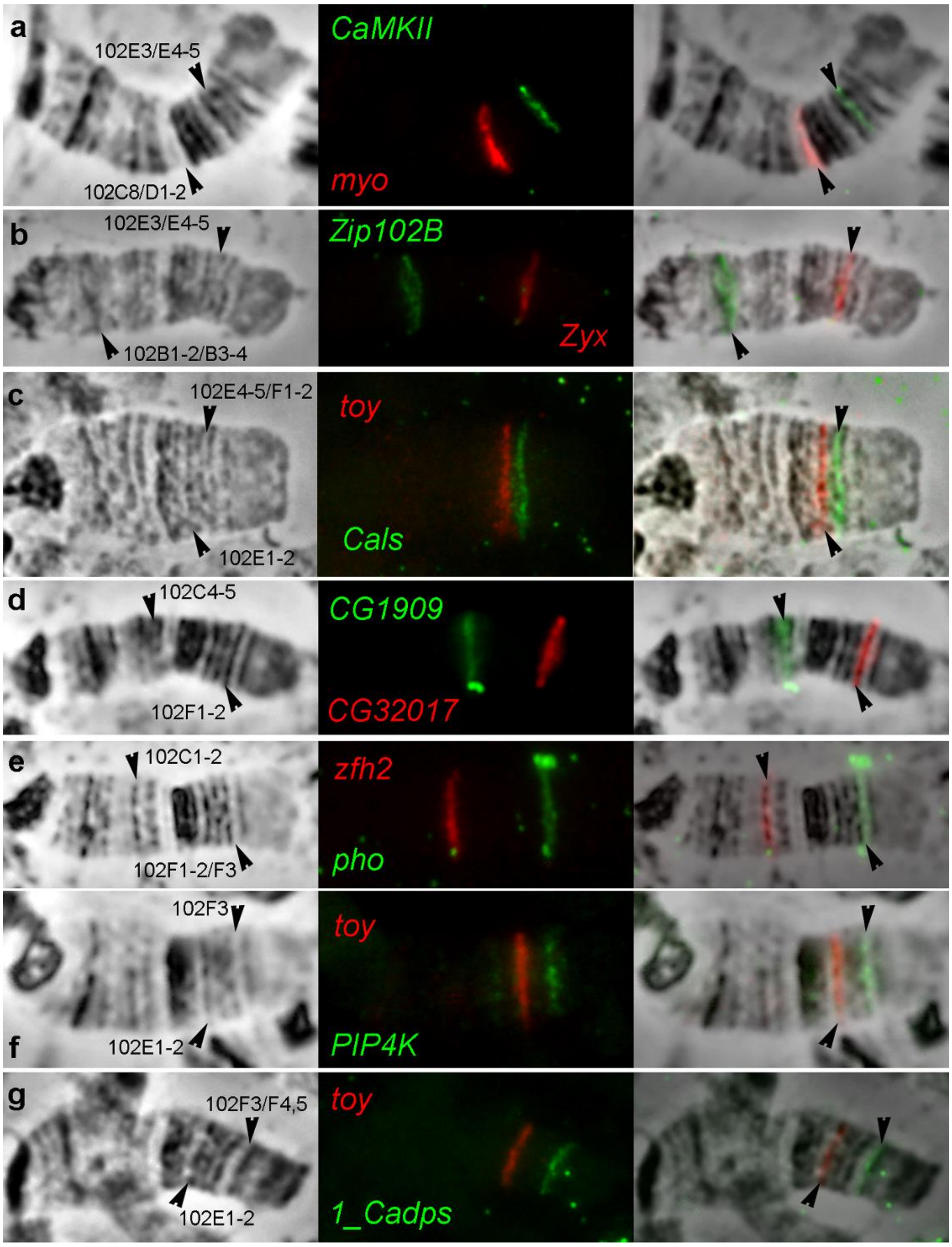

Fig. S7 FISH on the polytene fourth chromosome. Part 5. The probes from the interbands predicted by the four chromatin state model (Zhimulev et al. 2014). From left to right: the phase-contrast microphotograph of the fourth chromosome, combined FISH signals, and their superposition. The red signals correspond to probes labeled with TAMRA fluorochrome, the green ones correspond to probes labeled with Fluorescein. The arrowheads indicate: **a** *myo* probe in the 102C8/D1-2 interband, *CaMKII* probe in the 102E3/E4-5 interband; **b** *Zip102B* probe in the 102B1-2/B3-4 interband, *Zyx* probe in the 102E3/E4-5 interband; **c** *toy* probe in the 102E1-2 band, *Cals* probe in the 102E4-5/F1-2 interband; **d** *CG1909* probe in the 102C4-5 band, *CG32017* probe in the 102F1-2 band; **e** *zfh2* probe in the 102C1-2 band, *pho* probe in the 102F1-2/F3 interband; **f** *toy* probe in the 102E1-2 band, *PIP4K* probe in the 102F3 band; **g** *toy* probe in the 102E1-2 band, *Cadps* probe in the 102F3/F4,5 interband.
