# Supplementary material for: Molecular and genetic organization of bands and interbands in the dot chromosome of Drosophila melanogaster
Source: Chromosoma. 2019 Apr 30;128(2):97–117. doi: 10.1007/s00412-019-00703-x (PMC6536484; doi:10.1007/s00412-019-00703-x)
Supplement: Supplementary file 6 — (PDF 1050 kb) [file 412_2019_703_MOESM6_ESM.pdf]

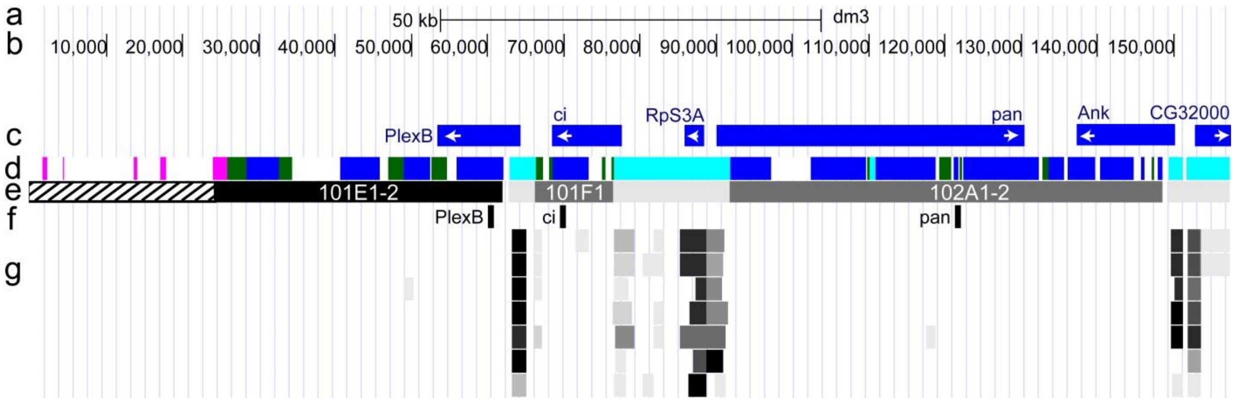

Fig. S8 The 101E1-2 – 102A1-2 section of the fourth chromosome. **a** the scale (kb) **b** the genomic coordinates (bp) **c** the genes (the white arrow indicates the direction) **d** the model of four chromatin types, the colors correspond to the type names: light blue rectangles – aquamarine chromatin, dark blue rectangles – lazurite chromatin, green rectangles – malachite chromatin, pink rectangles – ruby chromatin (Zhimulev *et al.*, 2014; Boldyreva *et al.*, 2017). White gaps correspond to regions where the model failed to determine a specific chromatin type. **e** The scheme of band and interband localization relative to the genomic coordinates. The band names are denoted. Black ruby-containing bands are marked with black rectangles, gray bands - with dark gray rectangles, and interbands - with light gray rectangles. White intervals correspond to model gaps which were not included in the bands or interbands. Shaded rectangle marks the proximal part of the fourth chromosome where chromatin types are not carefully defined. **f** FISH probes. **g** CHRIZ and WDS proteins localization in different cell cultures.

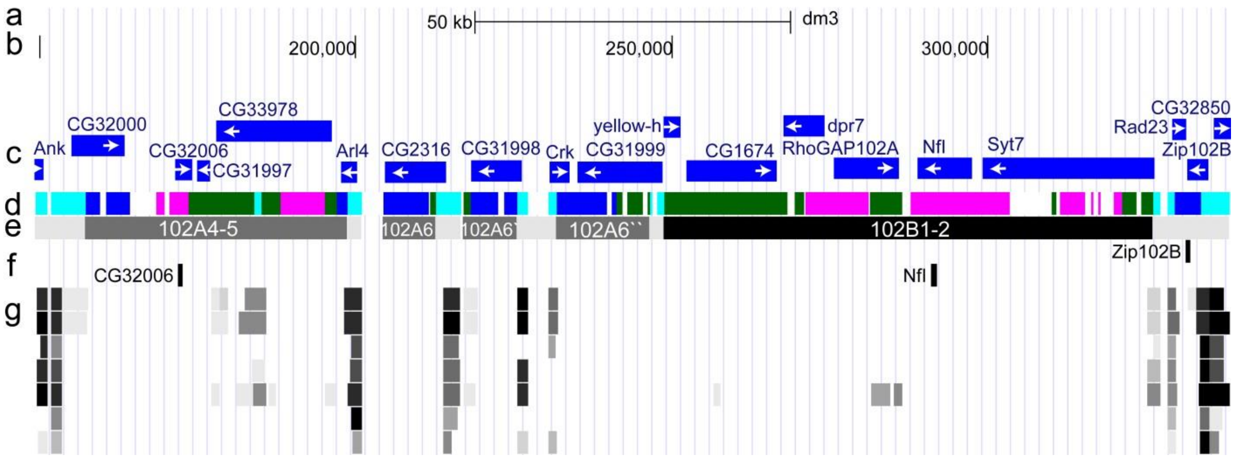

Fig. S9 The 102A4-5 – 102B1-2 section of the fourth chromosome. **a** the scale (kb) **b** the genomic coordinates (bp) **c** the genes (the white arrow indicates the direction) **d** the model of four chromatin types, the colors correspond to the type names: light blue rectangles – aquamarine chromatin, dark blue rectangles – lazurite chromatin, green rectangles – malachite chromatin, pink rectangles – ruby chromatin (Zhimulev *et al.*, 2014; Boldyreva *et al.*, 2017). White gaps correspond to regions where the model failed to determine a specific chromatin type. **e** The scheme of band and interband localization relative to the genomic coordinates. The band names are denoted. Black ruby-containing bands are marked with black rectangles, gray bands - with dark gray rectangles, and interbands - with light gray rectangles. White intervals correspond to model gaps which were not included in the bands or interbands. **f** FISH probes. **g** CHRIZ and WDS proteins localization in different cell cultures.

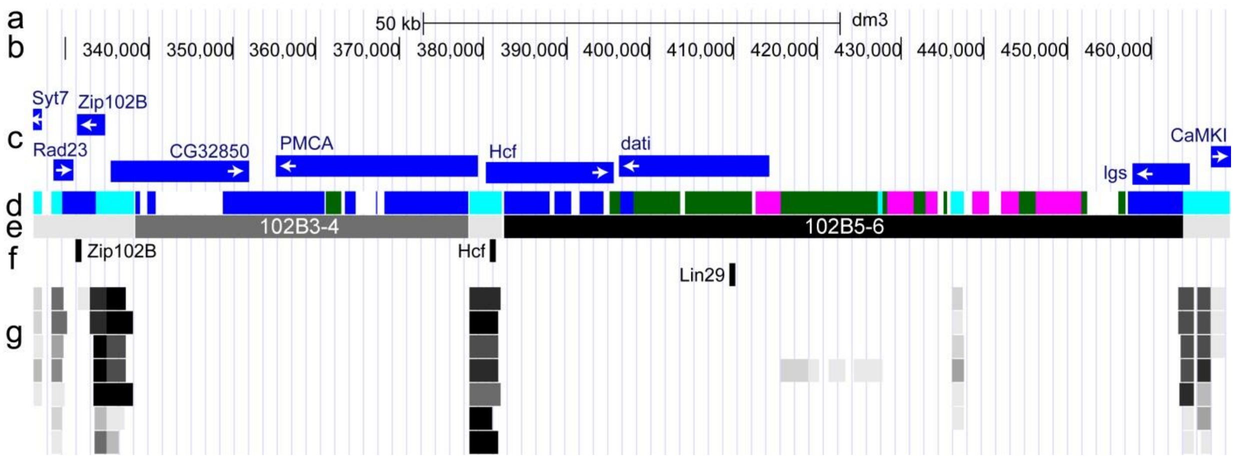

Fig. S10 The 102B3-4 – 102B5-6 section of the fourth chromosome. **a** the scale (kb) **b** the genomic coordinates (bp) **c** the genes (the white arrow indicates the direction) **d** the model of four chromatin types, the colors correspond to the type names: light blue rectangles – aquamarine chromatin, dark blue rectangles – lazurite chromatin, green rectangles – malachite chromatin, pink rectangles – ruby chromatin (Zhimulev *et al*., 2014; Boldyreva *et al*., 2017). White gaps correspond to regions where the model failed to determine a specific chromatin type. **e** The scheme of band and interband localization relative to the genomic coordinates. The band names are denoted. Black ruby-containing bands are marked with black rectangles, gray bands - with dark gray rectangles, and interbands - with light gray rectangles. White intervals correspond to model gaps which were not included in the bands or interbands. **f** FISH probes. **g** CHRIZ and WDS proteins localization in different cell cultures.

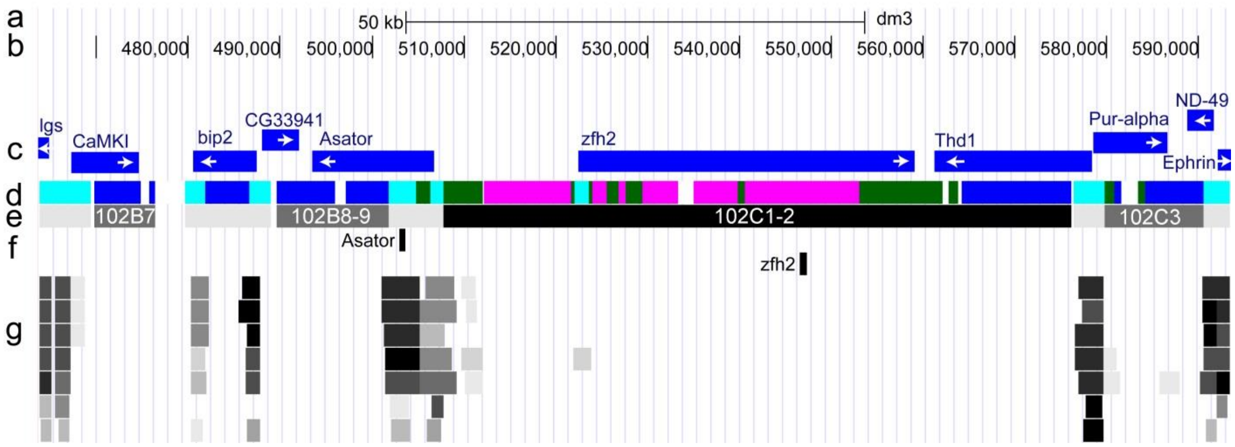

Fig. S11 The 102B7 – 102C3 section of the fourth chromosome. **a** the scale (kb) **b** the genomic coordinates (bp) **c** the genes (the white arrow indicates the direction) **d** the model of four chromatin types, the colors correspond to the type names: light blue rectangles – aquamarine chromatin, dark blue rectangles – lazurite chromatin, green rectangles – malachite chromatin, pink rectangles – ruby chromatin (Zhimulev *et al*., 2014; Boldyreva *et al*., 2017). White gaps correspond to regions where the model failed to determine a specific chromatin type. **e** The scheme of band and interband localization relative to the genomic coordinates. The band names are denoted. Black ruby-containing bands are marked with black rectangles, gray bands - with dark gray rectangles, and interbands - with light gray rectangles. White intervals correspond to model gaps which were not included in the bands or interbands. **f** FISH probes. **g** CHRIZ and WDS proteins localization in different cell cultures.

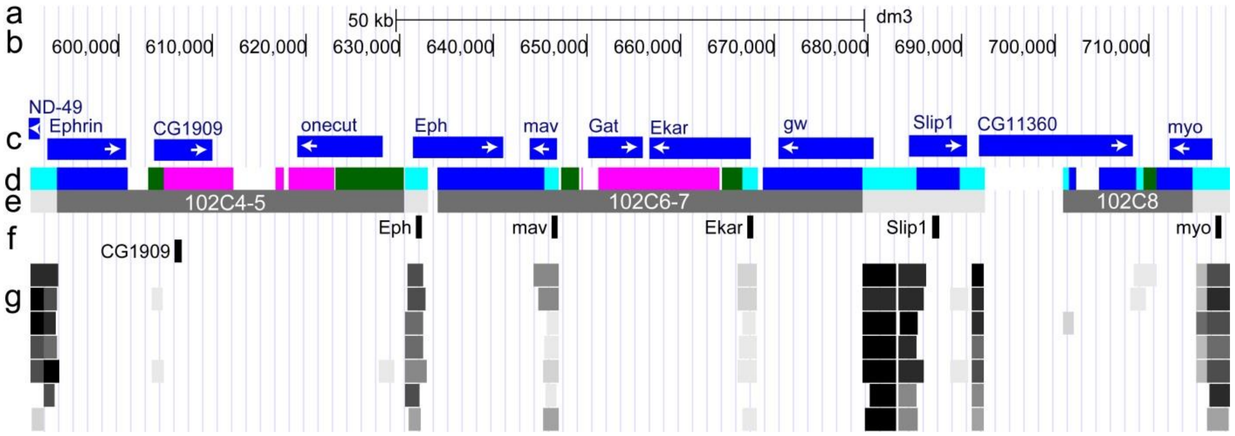

Fig. S12 The 102C4-5 – 102C8 section of the fourth chromosome. **a** the scale (kb) **b** the genomic coordinates (bp) **c** the genes (the white arrow indicates the direction) **d** the model of four chromatin types, the colors correspond to the type names: light blue rectangles – aquamarine chromatin, dark blue rectangles – lazurite chromatin, green rectangles – malachite chromatin, pink rectangles – ruby chromatin (Zhimulev *et al*., 2014; Boldyreva *et al*., 2017). White gaps correspond to regions where the model failed to determine a specific chromatin type. **e** The scheme of band and interband localization relative to the genomic coordinates. The band names are denoted. Black ruby-containing bands are marked with black rectangles, gray bands - with dark gray rectangles, and interbands - with light gray rectangles. White intervals correspond to model gaps which were not included in the bands or interbands. **f** FISH probes. **g** CHRIZ and WDS proteins localization in different cell cultures.

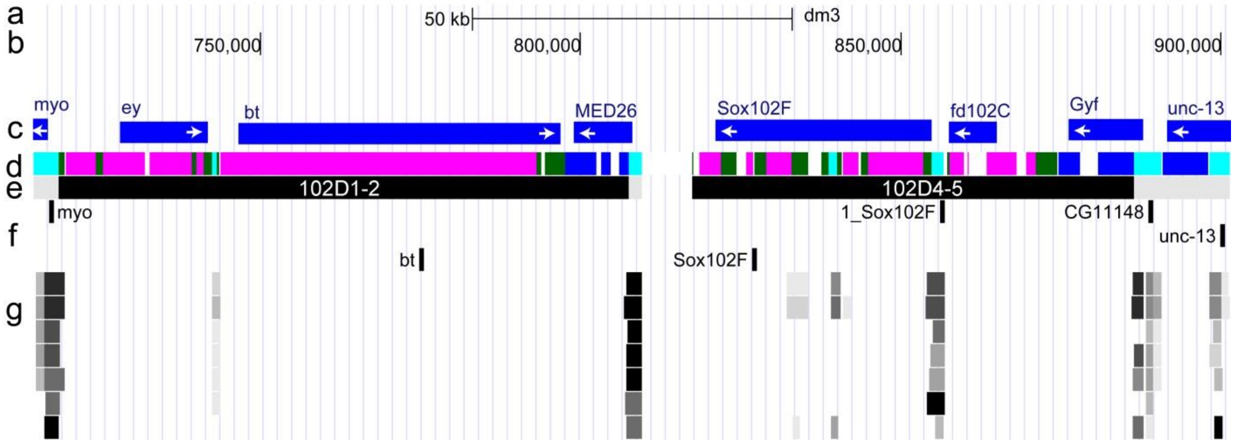

Fig. S13 The 102D1-2 – 102D4-5 section of the fourth chromosome. **a** the scale (kb) **b** the genomic coordinates (bp) **c** the genes (the white arrow indicates the direction) **d** the model of four chromatin types, the colors correspond to the type names: light blue rectangles – aquamarine chromatin, dark blue rectangles – lazurite chromatin, green rectangles – malachite chromatin, pink rectangles – ruby chromatin (Zhimulev *et al*., 2014; Boldyreva *et al*., 2017). White gaps correspond to regions where the model failed to determine a specific chromatin type. **e** The scheme of band and interband localization relative to the genomic coordinates. The band names are denoted. Black ruby-containing bands are marked with black rectangles, gray bands - with dark gray rectangles, and interbands - with light gray rectangles. White intervals correspond to model gaps which were not included in the bands or interbands. **f** FISH probes. **g** CHRIZ and WDS proteins localization in different cell cultures.

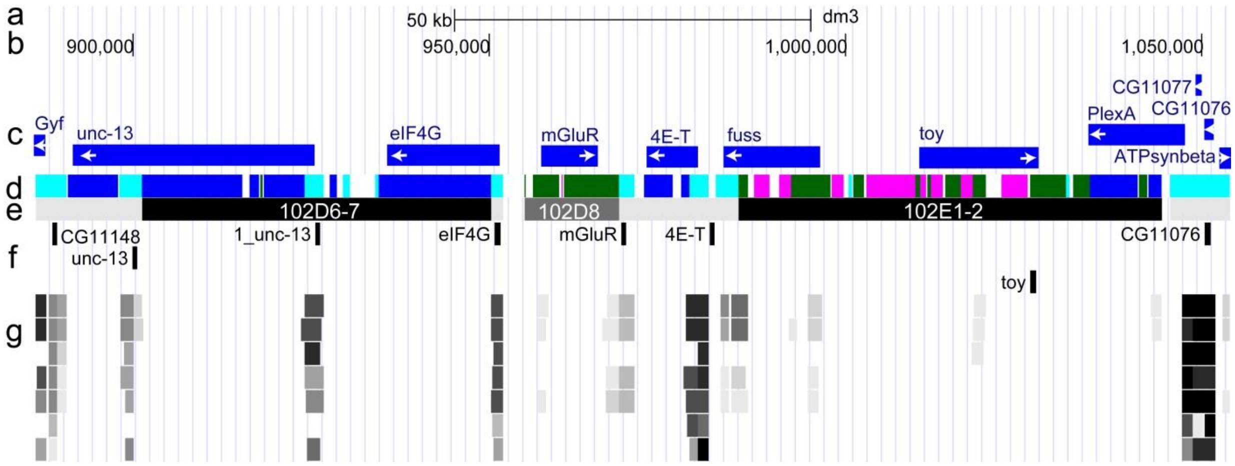

Fig. S14 The 102D6-7 – 102E1-2 section of the fourth chromosome. **a** the scale (kb) **b** the genomic coordinates (bp) **c** the genes (the white arrow indicates the direction) **d** the model of four chromatin types, the colors correspond to the type names: light blue rectangles – aquamarine chromatin, dark blue rectangles – lazurite chromatin, green rectangles – malachite chromatin, pink rectangles – ruby chromatin (Zhimulev *et al*., 2014; Boldyreva *et al*., 2017). White gaps correspond to regions where the model failed to determine a specific chromatin type. **e** The scheme of band and interband localization relative to the genomic coordinates. The band names are denoted. Black ruby-containing bands are marked with black rectangles, gray bands - with dark gray rectangles, and interbands - with light gray rectangles. White intervals correspond to model gaps which were not included in the bands or interbands. **f** FISH probes. **g** CHRIZ and WDS proteins localization in different cell cultures.

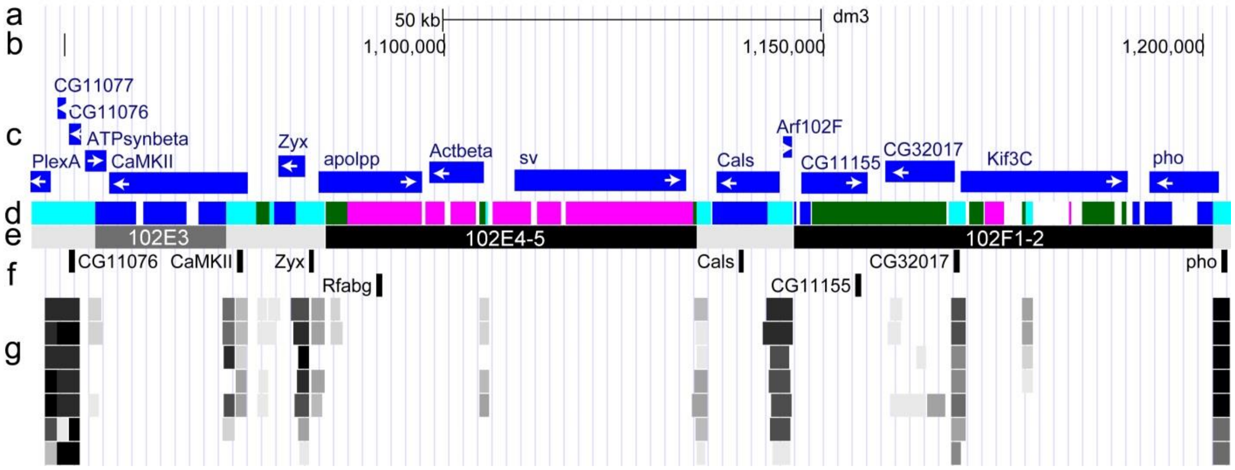

Fig. S15 The 102E3 – 102F1-2 section of the fourth chromosome. **a** the scale (kb) **b** the genomic coordinates (bp) **c** the genes (the white arrow indicates the direction) **d** the model of four chromatin types, the colors correspond to the type names: light blue rectangles – aquamarine chromatin, dark blue rectangles – lazurite chromatin, green rectangles – malachite chromatin, pink rectangles – ruby chromatin (Zhimulev *et al*., 2014; Boldyreva *et al*., 2017). White gaps correspond to regions where the model failed to determine a specific chromatin type. **e** The scheme of band and interband localization relative to the genomic coordinates. The band names are denoted. Black ruby-containing bands are marked with black rectangles, gray bands - with dark gray rectangles, and interbands - with light gray rectangles. White intervals correspond to model gaps which were not included in the bands or interbands. **f** FISH probes. **g** CHRIZ and WDS proteins localization in different cell cultures.

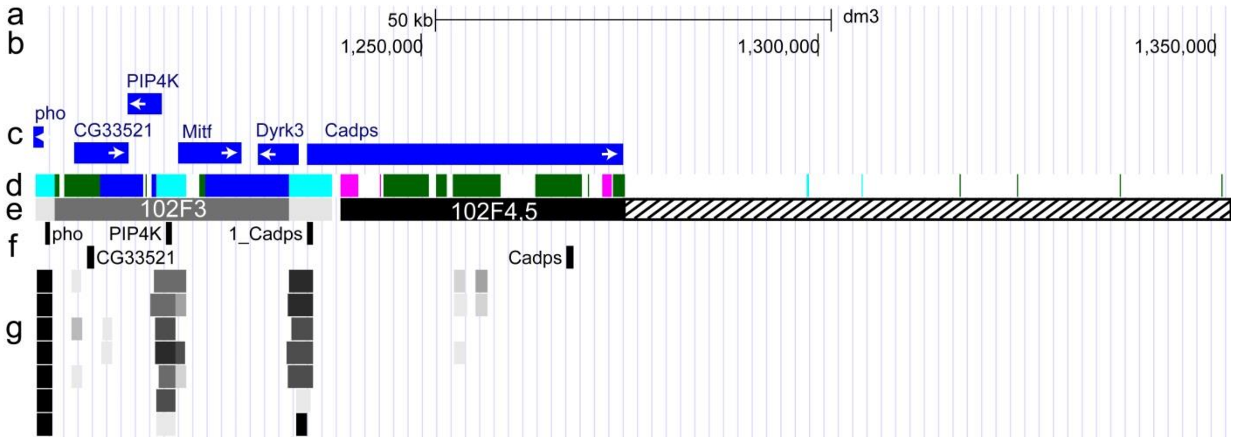

Fig. S16 The 102F3 – 102F4,5 section of the fourth chromosome. **a** the scale (kb) **b** the genomic coordinates (bp) **c** the genes (the white arrow indicates the direction) **d** the model of four chromatin types, the colors correspond to the type names: light blue rectangles – aquamarine chromatin, dark blue rectangles – lazurite chromatin, green rectangles – malachite chromatin, pink rectangles – ruby chromatin (Zhimulev *et al*., 2014; Boldyreva *et al*., 2017). White gaps correspond to regions where the model failed to determine a specific chromatin type. **e** The scheme of band and interband localization relative to the genomic coordinates. The band names are denoted. Black ruby-containing bands are marked with black rectangles, gray bands - with dark gray rectangles, and interbands - with light gray rectangles. White intervals correspond to model gaps which were not included in the bands or interbands. Shaded rectangle marks the end of the fourth chromosome where chromatin types are not carefully defined. **f** FISH probes. **g** CHRIZ and WDS proteins localization in different cell cultures.
