# Supplementary material for: Molecular and genetic organization of bands and interbands in the dot chromosome of Drosophila melanogaster
Source: Chromosoma. 2019 Apr 30;128(2):97–117. doi: 10.1007/s00412-019-00703-x (PMC6536484; doi:10.1007/s00412-019-00703-x)
Supplement: Supplementary file 7 — (PDF 346 kb) [file 412_2019_703_MOESM7_ESM.pdf]

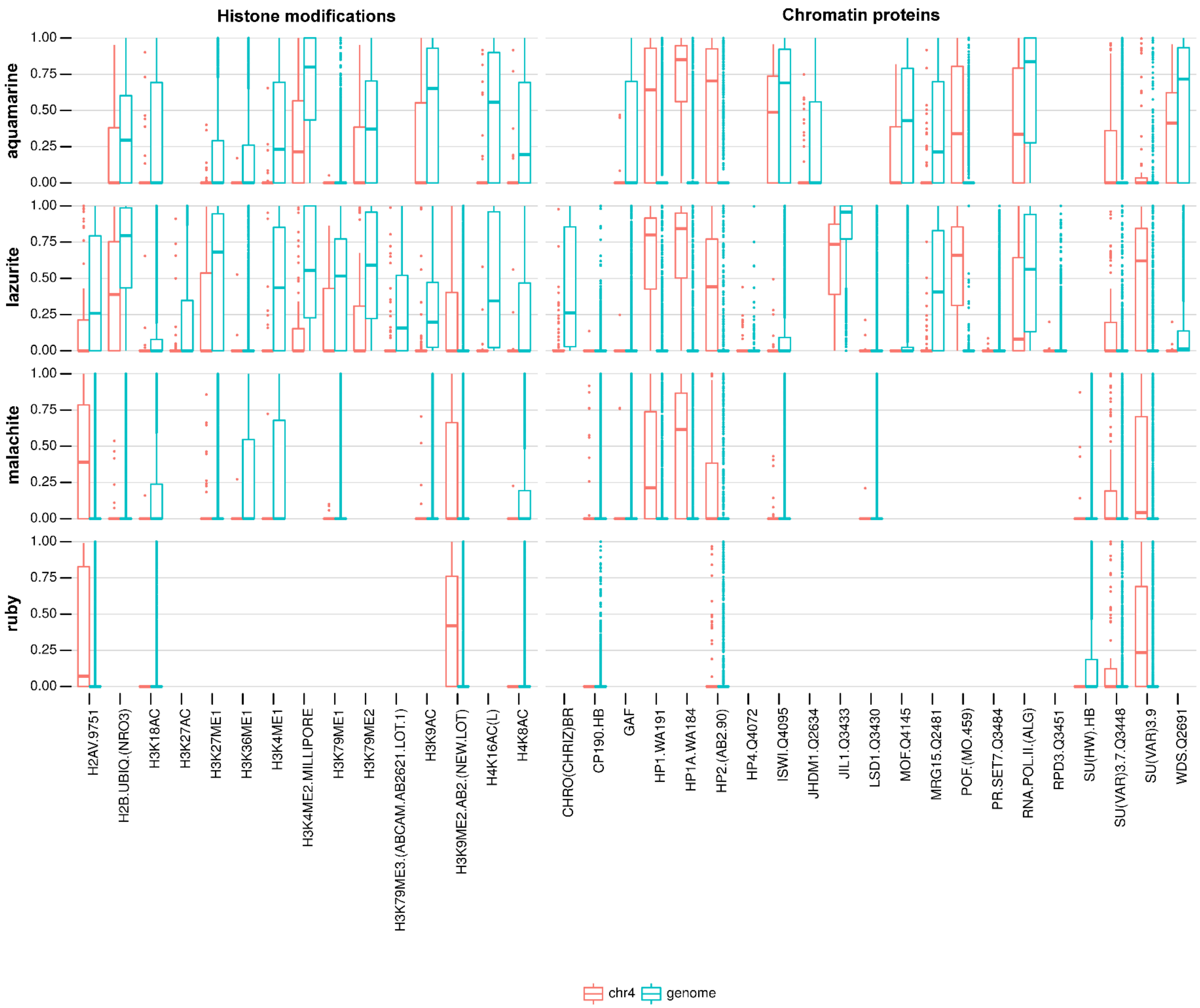

Fig. S17 The differences in the distribution of proteins in the four chromatin types of the fourth chromosome and the rest of the *D. melanogaster* genome. Statistical analysis was performed using the Mann-Whitney U test. On the horizontal axis, all histone modifications (the left part of the graphs) and chromatin proteins (the right part) are noted, the proportion of which in the determined chromatin state of the fourth chromosome is significantly different from that for the rest of the genome. The vertical axis shows the portion occupied by the protein in a particular chromatin state. Shown are boxplot diagrams for proteins that have significant differences (p<0,001).
