# Supplementary material for: Molecular and genetic organization of bands and interbands in the dot chromosome of Drosophila melanogaster
Source: Chromosoma. 2019 Apr 30;128(2):97–117. doi: 10.1007/s00412-019-00703-x (PMC6536484; doi:10.1007/s00412-019-00703-x)
Supplement: Supplementary file 8 — (PDF 188 kb) [file 412_2019_703_MOESM8_ESM.pdf]

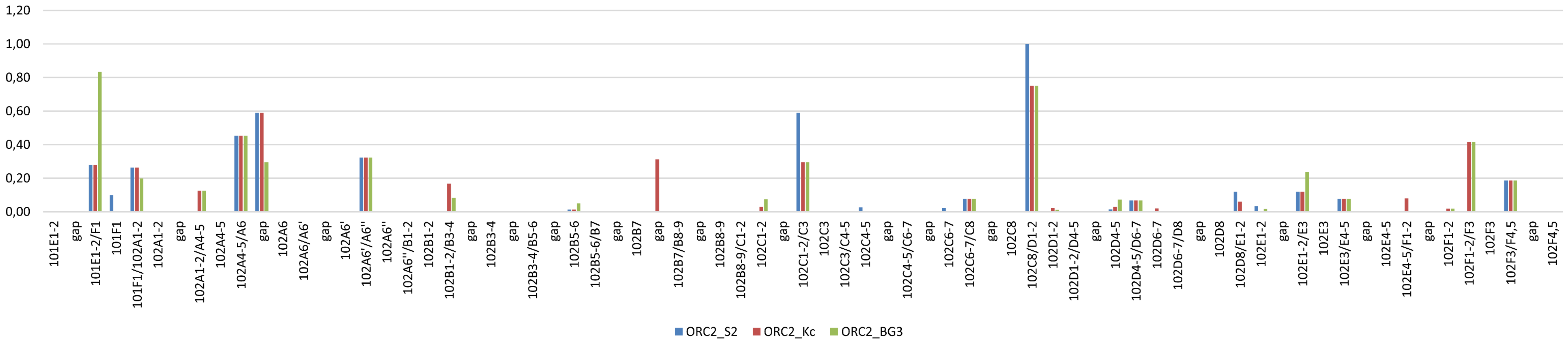

Fig. S18 The density of the ORC2 protein distribution in the structures of the fourth chromosome. The horizontal axis shows the bands and interbands of the fourth chromosome. The vertical axis shows the density of ORC2 binding sites (pcs/kb of the structure under consideration). Chart legend indicates the color code of cell line. Gaps correspond to model gaps which were not included in the bands or interbands.
