# Supplementary material for: Molecular and genetic organization of bands and interbands in the dot chromosome of Drosophila melanogaster
Source: Chromosoma. 2019 Apr 30;128(2):97–117. doi: 10.1007/s00412-019-00703-x (PMC6536484; doi:10.1007/s00412-019-00703-x)
Supplement: Supplementary file 9 — (PDF 399 kb) [file 412_2019_703_MOESM9_ESM.pdf]

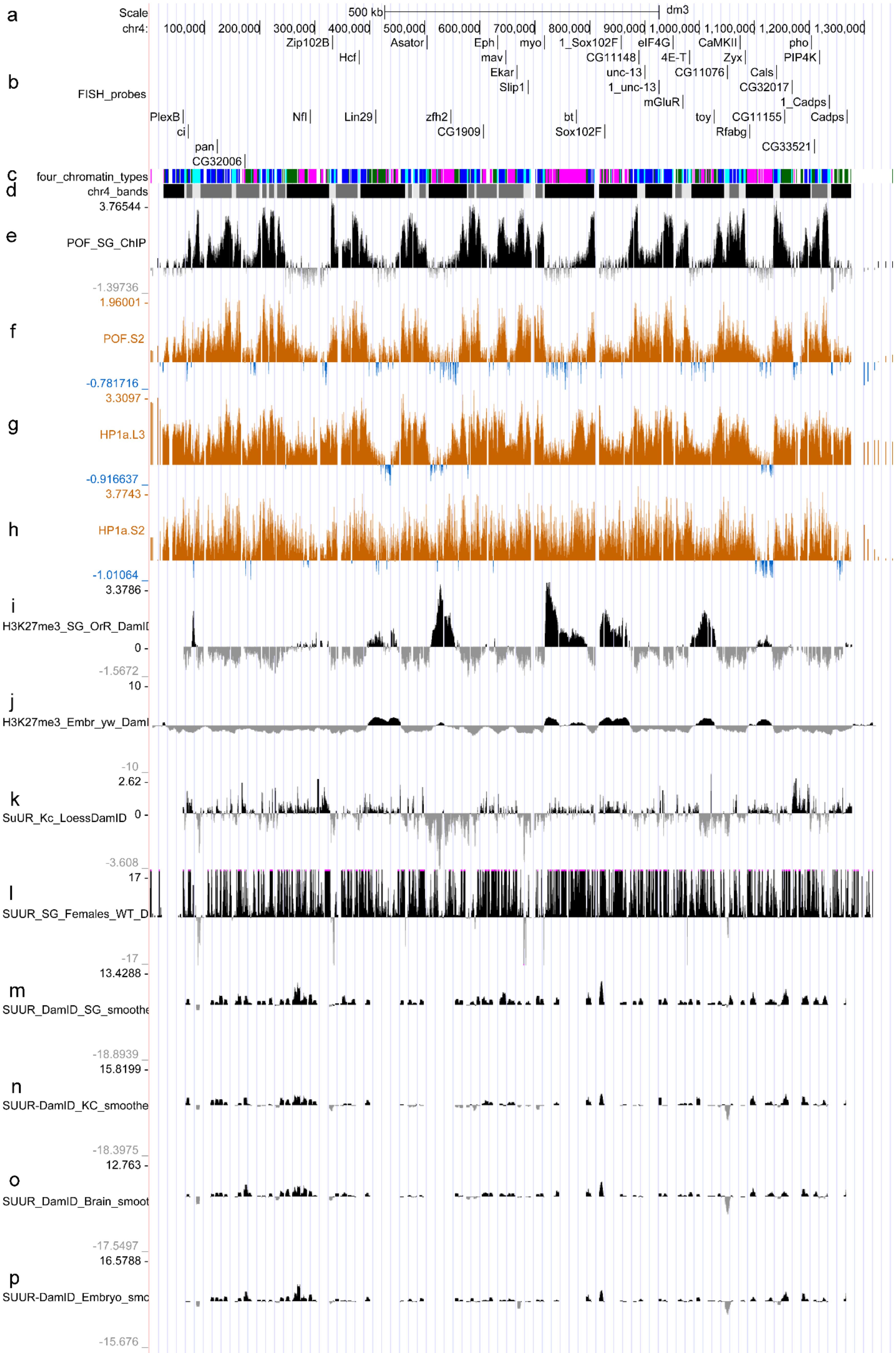

Fig. S19 The passport of protein distribution in the chromatin of the fourth chromosome. **a** the scale and genomic coordinates **b** FISH probes **c** four chromatin types (see materials and methods) **d** scheme of bands and interbands **e** POF distribution in salivary gland chromosomes obtained by ChIP method (Lundberg et al., 2013, Johansson and Larsson, 2014) **f** POF distribution in S2 cells (modENCODE data) **g** HP1a distribution in third instar larvae (modENCODE data) **h** HP1a distribution in S2 cells (modENCODE data) **i** H3K27 distribution in salivary gland chromosomes obtained by ChIP method (Sher et al., 2012) **j** H3K27 distribution in embryos obtained by ChIP method (Posukh et al., 2017) **k** SUUR distribution in Kc cells obtained by DamID method (Filion et al., 2010) **l** SUUR distribution in salivary glands obtained by DamID method (Posukh et al., 2017) **m** SUUR distribution in salivary glands obtained by DamID method (Maksimov et al., 2014) **n** SUUR distribution in Kc cells obtained by DamID method (Maksimov et al., 2014) **o** SUUR distribution in brain obtained by DamID method (Maksimov et al., 2014) **p** SUUR distribution in embryos obtained by DamID method (Maksimov et al., 2014).
