# Supplementary material for: Molecular and genetic organization of bands and interbands in the dot chromosome of Drosophila melanogaster
Source: Chromosoma. 2019 Apr 30;128(2):97–117. doi: 10.1007/s00412-019-00703-x (PMC6536484; doi:10.1007/s00412-019-00703-x)
Supplement: Supplementary file 10 — (PDF 155 kb) [file 412_2019_703_MOESM10_ESM.pdf]

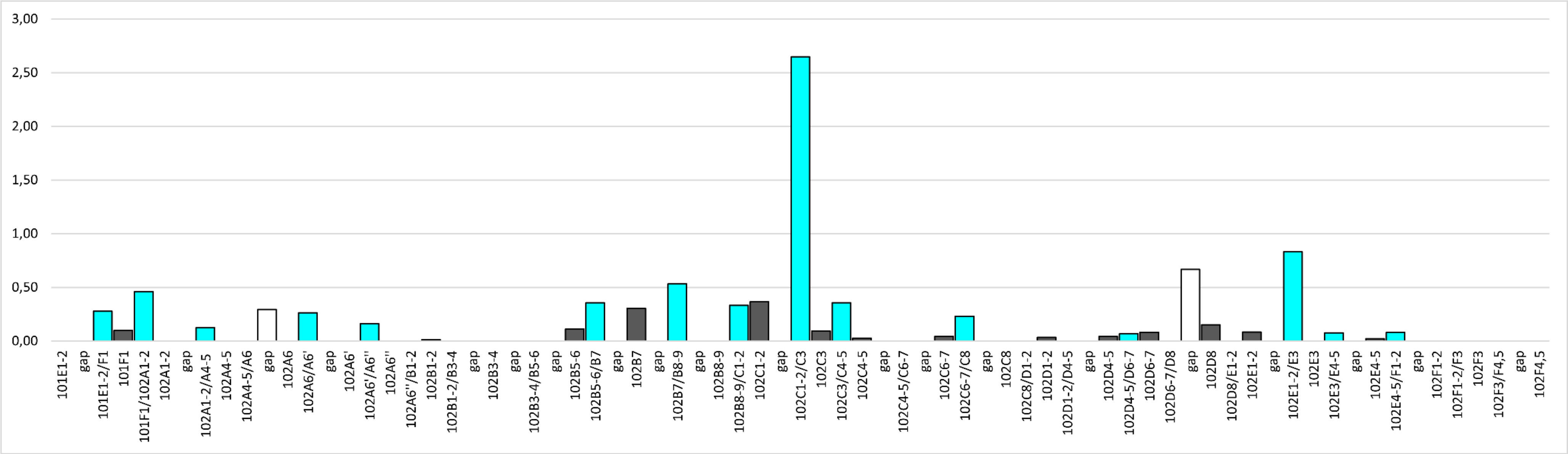

Fig. S20 The density of *P*-element insertions in the morphological structures of the fourth chromosome. The horizontal axis shows the bands and interbands of the fourth chromosome. The vertical axis shows the density of *P*-element insertions (pcs/kb of the structure under consideration). The blue bars correspond to the interbands, the gray ones to the bands, the white ones to the band gaps (model gaps which were not included in the bands or interbands).
