# Supplementary material for: Molecular and genetic organization of bands and interbands in the dot chromosome of Drosophila melanogaster
Source: Chromosoma. 2019 Apr 30;128(2):97–117. doi: 10.1007/s00412-019-00703-x (PMC6536484; doi:10.1007/s00412-019-00703-x)
Supplement: Supplementary file 11 — (PDF 193 kb) [file 412_2019_703_MOESM11_ESM.pdf]

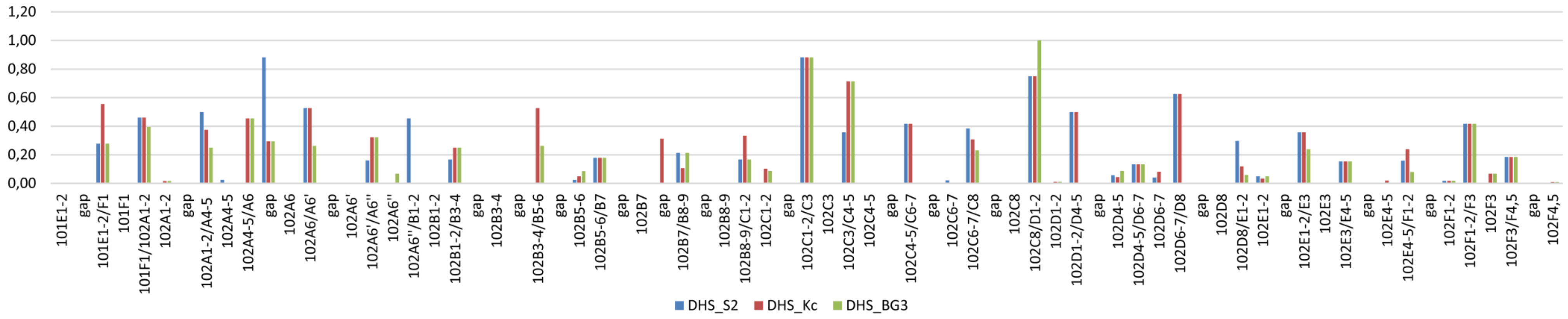

Fig. S21 The density of DNaseI hypersensitive sites (DHS) in the morphological structures of the fourth chromosome. The horizontal axis shows the bands and interbands of the fourth chromosome. The vertical axis shows the density of DHS (pcs/kb of the structure under consideration). Chart legend indicates the color code of cell line. Gaps correspond to model gaps which were not included in the bands or interbands.
