# Supplementary material for: Molecular and genetic organization of bands and interbands in the dot chromosome of Drosophila melanogaster
Source: Chromosoma. 2019 Apr 30;128(2):97–117. doi: 10.1007/s00412-019-00703-x (PMC6536484; doi:10.1007/s00412-019-00703-x)
Supplement: Supplementary file 3 — (PDF 379 kb) [file 412_2019_703_MOESM3_ESM.pdf]

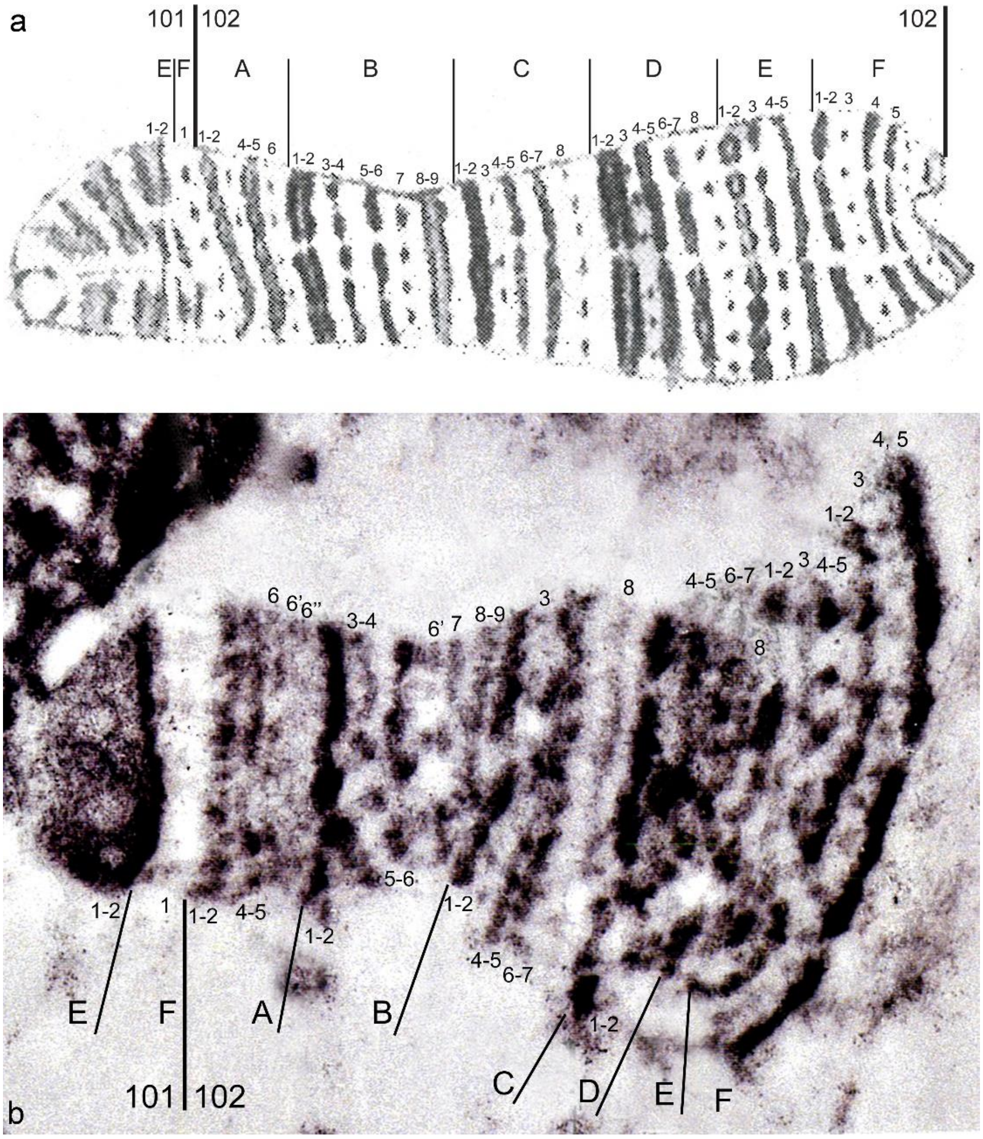

Fig. S1 Images of the *D. melanogaster* fourth chromosome **a** The map by C. Bridges with the bands numbered in accordance with Saura *et al.* (2002). **b** Electron microscopic image of the fourth polytene chromosome of *Drosophila*. The image shows the band numbers as in Fig. S1 (a). The picture was kindly provided by V. F. Semeshin.
