# Supplementary material for: Molecular and genetic organization of bands and interbands in the dot chromosome of Drosophila melanogaster
Source: Chromosoma. 2019 Apr 30;128(2):97–117. doi: 10.1007/s00412-019-00703-x (PMC6536484; doi:10.1007/s00412-019-00703-x)
Supplement: Supplementary file 13 — (PDF 190 kb) [file 412_2019_703_MOESM13_ESM.pdf]

Table S3 Localization of bands and interbands of the fourth chromosome on the molecular map of the Drosophila genome

Band/interband	Start (dm3)	End (dm3)	FISH probes	Genetic content
101E1-2	24000	61999	PlexB	Coding part (*PlexB*)
101E1-2/F1	62800	66399		5’UTR (*PlexB*)
101F1	66400	76599	ci	Coding part (*ci*)
101F1/102A1-2	76600	91799		5’UTRs of two genes (*ci*, *pan*) and one whole gene (*RpS3A*)
102A1-2	91800	148599	pan	Coding parts of two genes (*pan*, *Ank*)
102A1-2/A4-5	149400	157399		5’UTRs head-to-head (*Ank*, *CG32000*)
102A4-5	157400	198799	CG32006	Polygenic (*CG32000*, *CG32006*, *CG31997*, *CG33978*, *Arl4*)
102A4-5/A6	198800	200999		5’UTR (*Arl4*)
102A6	204400	212799		Coding part (*CG2316*)
102A6/A6'	212800	216599		5’UTR (*CG2316*)
102A6'	217000	225599		Coding part (*CG31998*)
102A6'/A6"	225600	231799		5’UTRs head-to-head (*CG31998*, *Crk*)
102A6"	231800	246599		Coding parts of two genes (*Crk*, *CG31999*)
102A6"/B1-2	246600	248799		5’UTRs head-to-head (*CG31999*, *yellow-h*)
102B1-2	248800	326199	NfI	Polygenic (*yellow-h*, *CG1674*, *dpr7*, *RhoGAP102A*, *NfI*, *Syt7*)
102B1-2/B3-4	326200	338199	Zip102B	5’UTRs of two genes (*Syt7*, *CG32850*) and two whole genes (*Rad23*, *Zip102B*)
102B3-4	338400	378199		Coding parts of two genes (*CG32850*, *PMCA*)
102B3-4/B5-6	378400	382199	Hcf	5’UTRs head-to-head (*PMCA*, *Hcf*)
102B5-6	382600	463799	Lin29	Polygenic (*Hcf*, *dati*, *Igs*)
102B5-6/B7	463800	469399		5’UTRs head-to-head (*Igs*, *CaMKI*)
102B7	469800	476399		Coding part (*CaMKI*)
102B7/B8-9	479600	488999		5’UTRs head-to-head (*bip2*, *CG33941*)
102B8-9	489600	501799		Coding parts of two genes (*CG33941*, *Asator*)
102B8-9/C1-2	501800	507799	Asator	5’UTR (*Asator*)
102C1-2	507800	576199	zfh2	Two genes (*zfh2*, *Thd1*)
102C1-2/C3	576400	579799		5’UTRs head-to-head (*Thd1*, *Pur-alpha*)
102C3	579800	590599		Coding parts of two genes (*Pur-alpha*, *ND-49*)
102C3/C4-5	590600	593399		5’UTRs head-to-head (*ND-49*, *Ephrin*)
102C4-5	593400	630399	CG1909	Polygenic (*Ephrin*, *CG1909*, *onecut*)
102C4-5/C6-7	630600	632999	Eph	5’UTR (*Eph*)
102C6-7	634000	679399	mav, Ekar	Polygenic (*Eph*, *mav*, *Gat*, *Ekar*, *gw*)
102C6-7/C8	679400	692399	Slip1	5’UTRs of two genes (*gw*, *CG11360*)and one whole gene (*Slip1*)
102C8	700800	714599		Coding parts of two genes (*CG11360*, *myo*)
102C8/D1-2	714600	718599	myo	5’UTRs head-to-head (*myo*, *ey*)
102D1-2	718600	807599	bt	Polygenic (*ey*, *bt*, *MED26*)
102D1-2/D4-5	807600	809599		5’UTR (*MED26*)
102D4-5	817400	886399	Sox102F, 1_Sox102F	Polygenic (*Sox102F*, *fd102C*, *Gyf*)
102D4-5/D6-7	886400	901399	CG11148, unc13	5’UTR (*Gyf*) and coding part of another gene (*unc-13*)
102D6-7	901400	950399	1_unc13	Coding parts of two genes (*unc-13*, *eIF4G*)
102D6-7/D8	950400	951999	eIF4G	5’UTR (*eIF4G*)
102D8	955000	968199		One whole gene (*mGluR*)
102D8/E1-2	968200	984999	mGluR, 4E-T	One whole gene (*4E-T*) and one 3’UTR (*fuss*)
102E1-2	985000	1044399	toy	Polygenic (*fuss*, *toy*, *PlexA*)
102E1-2/E3	1045600	1053999	CG11076	5’UTRs of two genes (*PlexA*, *ATPsynbeta*) and two whole genes (*CG11077*, *CG11076*)
102E3	1054000	1071199		Coding parts of two genes (*ATPsynbeta*, *CaMKII*)
102E3/E4-5	1071200	1084199	CaMKII, Zyx	5’UTRs of two genes (*CaMKII*, *apolpp*) and one whole gene (*Zyx*)
102E4-5	1084400	1133399	Rfabg	Polygenic (*apolpp*, *Actbeta*, *sv*)
102E4-5/F1-2	1133400	1145999	Cals	Two whole genes (*Cals*, *Arf102F*)
102F1-2	1146200	1201399	CG11155, CG32017	Polygenic (*CG11155*, *CG32017*, *Kif3C*, *pho*)
102F1-2/F3	1201400	1203799	pho	5’UTR (*pho*)
102F3	1203800	1233399	CG33521, PIP4K	Polygenic (*CG33521*, *PIP4K*, *Mitf*, *Dyrk3*)
102F3/F4,5	1233400	1238799	1_Cadps	5’UTRs head-to-head (*Dyrk3*, *Cadps*)
102F4,5	1239800	1275599	Cadps	Coding part (*Cadps*)
