# Supplementary material for: Molecular and genetic organization of bands and interbands in the dot chromosome of Drosophila melanogaster
Source: Chromosoma. 2019 Apr 30;128(2):97–117. doi: 10.1007/s00412-019-00703-x (PMC6536484; doi:10.1007/s00412-019-00703-x)
Supplement: Supplementary file 12 — (PDF 179 kb) [file 412_2019_703_MOESM12_ESM.pdf]

Table S1. Oligonucleotides used to obtain FISH probes from bands predicted by the four chromatin state model

Probe	Forward primer	Reverse primer	Probe coordinates (dm3)
PlexB	5`-ACAACCTTCGCCTGATATT-3`	5`-TGTTCCGCTTGCTTAGAG-3`	chr4:59994-60799
ci	5'-CATCCCAGGTGTCTTCTATAC-3'	5'-GTTCTCGGTAGTGGTTTGA-3'	chr4:69435-70241
pan	5`-CCAAGTCGCAGCTCTATT-3`	5`-GCCTAAGAGTATCCACATCC-3`	chr4:121380-122168
CG32006	5'-TCCACCACACGCATTATT-3'	5'-GAGTTGGTTCTGTGTTCCT-3'	chr4:171797-172593
NfI	5'-TCTCACACACCGACATTAG-3'	5'-CCCAAGCCGAAGAGTTTA-3'	chr4:291032-291853
Lin29	5'-CATACGGACGGAAAGACTAA-3'	5'-CCACACATAGGAGGAACAA-3'	chr4:409421-410217
zfh2	5'-GCGCATCCTCCGAAATA-3'	5'-GGTTGAGAGTAAGCGTAGAG-3'	chr4:546526-547326
CG1909	5'-GAGCCGAGACGTACTTAAA-3'	5'-TCCCTCCGACTATCCTATC-3'	chr4:605956-606771
bt	5'-TGTAAGGGCGTCGTAAAG-3'	5'-CCTGGTCTATCTGGTTCATC-3'	chr4:774728-775516
Sox102F	5'-GAGGTGAACCACCTGATAAA-3'	5'-GACCCTGGCAACGATAAT-3'	chr4:826731-827536
toy	5'-GTCAGAAACGCTTCCTACT-3'	5'-CTTCCCGTTAGGTCAGATAC-3'	chr4:1025961-1026761
Rfabg	5'-CATCGACGGCAGTATCAA-3'	5'-TACTTTCTCGGCTCCATAAC-3'	chr4:1091079-1091901
CG11155	5`-CGCAGTTCCCGAGATATT-3`	5`-AGAGCGACGCGAATAAA-3`	chr4:1154175-1154991
CG33521	5`-GCTTTGTGGCGATAGTTTAC-3`	5`-CTAGACCTCGGCGTTTATAG-3`	chr4:1207907-1208708
Cadps*	5’-TAGCTTATGTGGTGTTACACG-3’	5’-AGATGCAACTTGAGCATAGTG-3’	chr4:1268218-1269117

*This probe from the end of the fourth chromosome was obtained by lowering the PCR elongation temperature to 60°C

Table S2. Oligonucleotides used to obtain FISH probes from open chromatin predicted by the four chromatin state model

Probe	Forward primer	Reverse primer	Probe coordinates (dm3)
Zip102B	5` – GGAGTCCACAATTAAGTCA – 3`	5` – GGGTATGCCCATGTTTATA – 3`	chr4:331263-331983
Hcf	5` – TATAGGGAAGCGTCTAGTT – 3`	5` – AAGACGACCATTAGCTCTT – 3`	chr4:380790-381582
Asator	5` – AGTTTCGTTGAACTTCCTAT – 3`	5` – CAACTGGTGTCCTCTATG – 3`	chr4:502957-503631
Eph	5` – CGTCCCTTAACAAGAATAC – 3`	5` – GCTTCTCGAGTTGTATCC – 3`	chr4:631665-632380
mav	5` – CCATTAGGGTTCCTTTGC – 3`	5` – CCGAATCCGATCTGGTTA – 3`	chr4:646226-646906
Ekar	5` – CCAATTCTGACCAAGGGT – 3`	5` – CCCTGCAGTTACATTCAC – 3`	chr4:667070-667779
Slip1	5` – CTACCAAGTCCTGTAGTAA – 3`	5` – TTGGAAGAGCAGAGACCA – 3`	chr4:686812-687535
myo	5` – CAAATAGACCCGAAGAAAC – 3`	5` – GCTTACAATCCATCCAATAA – 3`	chr4:717011-717731
1_Sox102F	5` – TGTTACCATCCCTATTATAC – 3`	5` – TCAACTCGGCTATTTATCC – 3`	chr4:856150-856850
CG11148	5` – CTTCGTAACGAGACCTAAA – 3`	5` – TGTGAAGGAGACAGTAATC – 3`	chr4:888766-889498
unc-13	5` – CAAGGGAGAAAGAAGAAGA – 3`	5` – ATTGCCCATGCAGTAATAAA – 3`	chr4:899961-900686
1_unc-13	5` – CAAGGGAGGGTTAAGTATA – 3`	5` – CTAAGCAACAGTAGCGAAA – 3`	chr4:925604-926283
eIF4G	5` – CTGGAGCCTTACTTATTTG – 3`	5` – CGGAAAGGGCTCATTATC – 3`	chr4:950896-951616
mGluR	5` – GCCTATATCCTAACCAATAA – 3`	5` – GTAGAATTTGAATCTCGAAG – 3`	chr4:968580-969277
4E-T	5` – TCTACGCATACAGTGATAG – 3`	5` – GTCCGATTTCGTAGCAATA – 3`	chr4:980957-981641
CG11076	5` – CTTCGTCCAGGTCATTAG – 3`	5` – GATCCACATCTGCTAGTC – 3`	chr4:1050543-1051253
CaMKII	5` – GGCATGGGATAAAGCATG – 3`	5` – CGCATGTAACCTGCAAAG – 3`	chr4:1072731-1073408
Zyx	5` – CTGTATGACCTTCCCTGT – 3`	5` – GTCGAGTGAGCGAATTTAT – 3`	chr4:1082117-1082864
Cals	5` – CGGCTTTGGAGCTAATAAA – 3`	5` – GGGCAGAATGGACTAAAG – 3`	chr4:1138891-1139561
CG32017	5` – AGAGCATTCCCATACATTC – 3`	5` – CAATAAGTCTTTCCCAACC – 3`	chr4:1167296-1167995
pho	5` – GATACGGTCCACAAATACA – 3`	5` – GGATTAGCAAAGGAGAAGT – 3`	chr4:1202556-1203255
PIP4K	5` – GAAATTCTCTGCCATATTAG – 3`	5` – TCGTACATATGACCTCTTG – 3`	chr4:1217808-1218513
1_Cadps	5` – CCACCATCGAAGTTATCG – 3`	5` – CGTGGAAACATGAGTATGA – 3`	chr4:1235568-1236287
